# Integration of nociceptive activity from orofacial, cranial and cervical regions in the trigeminocervical nucleus: a scoping review with clinical implications

**DOI:** 10.22514/jofph.2025.042

**Published:** 2025-09-12

**Authors:** Felix Pankrath, Elisa Bizetti Pelai, Ana Izabela Sobral de Oliveira-Souza, Fatemeh Baghbaninaghadehi, Liz Dennett, Peter Svensson, Harry von Piekartz, Susan Armijo-Olivo

**Affiliations:** ^1^Faculty of Economics and Social Sciences, University of Applied Sciences Osnabrück, 49076 Osnabrück, NDS, Germany; ^2^Post-Graduation Program in Oral and Maxillofacial Surgery, Piracicaba Dental School, State University of Campinas, UNICAMP, 13083-857 Piracicaba, SP, Brazil; ^3^Obstetrics and Gynaecology, IWK Health Center, Halifax, NS B3K 6R8, Canada; ^4^Geoffrey and Robyn Sperber Health Sciences Library, University of Alberta, Edmonton, AB T6G 1C9, Canada; ^5^Faculty of Dentistry, National University of Singapore, 119077, Singapore, Singapore; ^6^Department of Physical Therapy, Faculties of Rehabilitation Medicine and Medicine and Dentistry, University of Alberta, Edmonton, AB T6G 2R3, Canada

**Keywords:** Spinal trigeminal nucleus, Trigeminocervical nucleus, Nociception, Pain perception, Neck pain, Facial pain, Headache, Cervicogenic pain, Temporomandibular disorders

## Abstract

A connection between orofacial, cranial and cervical regions has been documented through the trigeminocervical nucleus (TCN), but the predominant direction (orofacial/cranial-to-cervical or cervical-to-orofacial/cranio) of this connection still remains unclear. This scoping review was designed to determine the dominant direction of the connection between these two areas. Searches were conducted by combining these concepts: TCN, cervical and face/jaw/head region. Eighty-three studies were included. The most predominant direction was reported to be from the orofacial/cranial to the cervical region, with most of the studies conducted in mammals. The clinical implications of these findings for the spread and referral of pain between these regions are examined and discussed.

## 1. Introduction

Painful disorders affecting the orofacial, cranial or cervical regions are very prevalent and disabling conditions [1]. These disorders encompass a range of conditions, such as orofacial pain (OFP), including odontogenic pains, temporomandibular disorders (TMD), headaches and neck pain. Many of these disorders often coexist and exhibit substantial overlap [2].

The close anatomical, biomechanical and neurophysiological connections among the orofacial, cranial and cervical structures may result in overlapping painful symptoms in patients with orofacial pain, headaches or cervical disorders. Often, patients do not present pure orofacial pain, headache or neck disorders, but mostly, they present a combination of them [3]. The anatomical connection among these areas at the trigeminocervical nucleus (TCN) seems crucial to understanding the complexity and overlap of symptoms and has been addressed previously [3, 4, 5, 6, 7, 8]. Because of the neural convergence between the orofacial, cranial and cervical regions within the TCN, a so-called referred-pain phenomenon occurs between these regions, meaning that nociceptive activity from one region can lead to the sensation of pain in another region, *e.g.*, referred pain [9]. This would mean that pain from cervical joints and muscles—innervated by cervical spinal nerves—can be perceived in regions innervated by the trigeminal nerve (*e.g.*, orofacial region). Conversely, pain transmitted by existing nociception in orofacial areas innervated by the trigeminal nerve could also be perceived in cervical regions [9]. For example, the neuro-anatomical connection of ophthalmic and cervical nociceptive afferents on second-order neurons at the caudal pars of the TCN seems pivotal to understanding the occurrence of headache and neck pain [10].

Studies in humans and animals have described the anatomy, structure and organization of the TCN [11, 12, 13], which extends from the medulla (pars oralis and pars interpolaris) to the first three segments of the cervical spine (pars caudalis) [11, 12, 13]. This connection is responsible for relaying various somatosensory modalities, including temperature, touch and nociception from the ipsilateral portion of the face, as well as nociceptive inputs from the supratentorial dura mater [14]. The TCN is considered the primary region where somatosensory information from the first three cervical spinal nerves converges along with trigeminal afferents [15]. The spinal trigeminal nucleus is subdivided anatomically into the pars oralis, pars interporalis and pars caudalis [16]. The pars oralis is the termination of afferent connections from the oral and nasal regions and contains circuits involved in brain stem reflexes. The pars interpolaris receives inputs from the ipsilateral face and forms the ascending anterior trigeminothalamic tract with the pars oralis. The pars caudalis also receives input from the face, forehead, cheek and jaw, as well as being involved in transmitting temperature sensation from these areas (see Fig. 1). The topographical representation of the inputs to the spinal trigeminal nucleus is inverted, such that the area encoding the forehead is represented ventrally (distally), and the area encoding the oral region is represented dorsally (proximally) [11, 17]. An extracranial origin of meningeal nociception in the form of collateral trigeminal afferents from functional connections between intra-and extracranial tissues in rats and humans has also been suggested [4, 18, 19].

**Fig. 1.** An overview of the trigeminal cervical nucleus (TCN) and their (inter)connections. Colored lines: green ophthalmic N, purple maxillary N, blue mandibular N, red craniocervical complex, yellow ascending pathways anterior trigeminothalamic tract (Source: own illustration, Harry von Piekartz).

Many studies have explored the connection between orofacial, cranial and cervical areas through the TCN for a long time, but until now, just five reviews have looked specifically into the relationship between the orofacial region and the cervical spine, especially regarding the convergence mechanism in the TCN. For example, Armijo-Olivo *et al*. [20] (2006) evaluated the anatomical, biomechanical and neurophysiological associations between the stomatognathic system and the cervical spine. The review supported the notion that cranial and cervical structures influence each other, and that craniofacial pain can be caused by all structures that are innervated by the upper three cervical nerves. Browne *et al*. [21] (1998) examined the association between craniofacial and craniocervical pain. The authors stated that craniofacial and craniocervical pain often occurs concomitantly. The reasoning behind a connection between the craniofacial and craniocervical regions was explained through a pain projection mechanism rooted in the convergence theory within the TCN. The authors concluded that pain in one region could cause pain in the other region, but an external or central-driven factor eliciting pain in both areas could not be excluded.

Haldeman and Dagenais (2001) [22] focused their review on the literature for cervicogenic headaches. It was suggested that an inflammatory process in any cervical structure could be a potential source of headaches. In another review, Ashkenazi and Levin (2007) [23] evaluated the effect of greater occipital nerve blocks (GON block) on migraine, tension-type headaches (TTH), cervicogenic headaches, and cluster headaches to support the association between cervical and cranial regions [23]. Piovesan *et al*. [24] (2003) aimed to follow up on the convergence mechanism itself. The convergence of trigeminal and upper cervical spinal nerves in the TCN was held responsible for the nociceptive overlap between the orofacial and cervical regions regarding immunohistochemical detections, metabolic changes, electrophysiological nerve stimulation, clinical studies and pharmacologic studies.

Even though there exists a substantial amount of evidence regarding the connection between the orofacial, cranial and cervical regions through the TCN, it is unclear whether a predominant direction (orofacial-cranial to cervical or cervical to orofacial-cranial direction) of this connection exists since this question has not been thoroughly explored in any previous review in a systematic way. Also, as mentioned above, none of the existing reviews has mapped the structures involved and the paths followed between these regions. The information emanated from this more granular analysis can directly impact decision-making in everyday clinical practice for clinicians working in this area; it would help clarify the structures involved in pain in these regions and the most common paths through the TCN. Exploring these connections in more detail could help focus the management toward the most appropriate region(s) when treating patients with these conditions.

Therefore, the objectives of this scoping review are:

(1) To determine whether a predominant direction (orofacial-cranial to cervical or cervical to orofacial-cranial direction) exists when analyzing the connection between orofacial, cranial and cervical regions through the TCN.

(2) To determine and describe methods, techniques and outcomes used to identify whether a connection between orofacial, cranial and cervical regions through the TCN exists, as well as its direction.

## 2. Methods

### 2.1 Study design

A scoping review was conducted following the methodology outlined by Arksey and O’Malley [25] and refined by the Joanna Briggs Institute. This scoping review was reported following the Preferred Reporting Items for Systematic Reviews and Meta-Analyses (PRISMA) extension for Scoping Reviews Checklist [26] and followed five stages: (1) identifying the research question; (2) identifying relevant studies; (3) selecting studies for analysis; (4) charting the data; and (5) collating, summarizing and reporting results [25]. Each step will be illustrated below.

### 2.2 Research question

Is there a dominant direction (*e.g.*, from orofacial-cranial to the cervical region or cervical region to orofacial-cranial region) when looking at the connections among cranial, orofacial and cervical regions through the TCN. Or are both directions predominant?

### 2.3 Eligibility criteria

No time or language restriction was applied. The eligibility criteria were defined in Table 1 and organized based on the domains of interest.

**Table 1.** Inclusion and exclusion criteria.

	Inclusion Criteria	Exclusion Criteria
Population	All kinds of mammals (*i.e.*, mice, cats, dogs, among others). Human beings of both sexes, older than 18 years.	Animals other than mammals and people younger than 18 years (children). Studies that investigate cell cultures or plants.
Concept	The concept of interest focused on the relationship between the cranial, orofacial and cervical regions through the TCN. Then, any study examining the mutual connection between the cervical, cranial and orofacial regions and considering the TCN as its relay station was included. Variables from at least two of these regions and the TCN had to be presented in the same study.	Any study that did not examine the interconnection between regions (cervical, cranial and orofacial regions) via the TCN.
Outcome	Any outcome that demonstrates a connection between the cervical, cranial and orofacial regions, involving the TCN. This includes various aspects such as anatomical, histological, biochemical, electrophysiological, neurophysiological, radiological, or clinical measures that provide evidence of the interrelationship between two regions through the TCN.	Any outcome that does not involve both regions and the investigation of the TCN was excluded.
Design	Observational studies (cross-sectional, cohort, case-control), experimental studies (animal studies, *in-vitro* studies) and clinical studies (RCT, CT).	Reviews, systematic reviews, qualitative studies, book chapters, abstracts, brief communications, commentaries, conference proceedings and editors’ letters.

*TCN: trigeminocervical nucleus; RCT: randomized clinical trial; CT: clinical trial.*

### 2.4 Data sources and searches

The search strategy was developed by an experienced health sciences librarian. An electronic search of the literature in Medline (Ovid MEDLINE(R) ALL), Embase (Ovid interface), American Psychological Association (APA) Psycinfo (Ovid interface), Cumulative Index to Nursing and Allied Health Literature (CINAHL) Plus with full text (EBSCOhost interface), Web of Science and Scopus databases was developed. The search included all relevant search terms from an earlier review and new terms suggested by the research team. The search combined subject headings and keyword terms for three concepts: TCN, cervical region and face/head/jaw region. No date, language, study design, or publication type limits were used. Reference lists of included articles were reviewed for additional studies. The full details of the search strategy can be found in the **Supplementary materials** (**Supplementary material 1**). The initial search was performed in April 2020, and the last update of the electronic searches was conducted on 23 March 2023. Additionally, reference lists of included studies and other published Scoping Reviews (SRs) were checked for additional studies on 23 March 2023.

### 2.5 Study selection

According to the Joanna Briggs Institute, the research question generated follows the “population, concept, context” (PCC) scheme [27].

Population of interest: Mammals or human participants (adults >18 years) with or without pain.

Concept: Determination of the most predominant direction of connection between the orofacial, cranial and cervical regions through the TCN (*i.e.*, from cranial, orofacial to the cervical region or *vice versa*). The following definitions were used to determine the regions of interest. “Orofacial region” similar to orofacial pain, is described to be in the region of the face anterior to the orbitomeatal line above the neck and up to the ear, including areas such as the temporomandibular joint (TMJ), teeth, the nose, nerves, the vascular system and any other structure between the chin, the ears and the forehead [28, 29]. “Cranial region” was defined as the anatomical area above the orbitomeatal line and/or nuchal ridge [30]. The “cervical/neck” region involves the anatomic region of the neck as the area between the superior nuchal line, the spines of the scapulae, and the superior border of the clavicles to the suprasternal notch [31]. In addition, the definition of Bogduk was used, which describes the following terms as belonging to the cervical region: intervertebral disc, zygapophysial joint, vertebral body, meninges (all layers and their corresponding supply), blood vessels, nerve sheaths, nerves, muscles [32].

Context: Open.

### 2.6 Data screening

The search results were transferred to an EndNote database (Clarivate, https://endnote.com/) and then uploaded to Covidence (https://www.covidence.org/) (Veritas Health Innovation, Melbourne, Australia), which was used to organize, screen and select the collected data. Two independent and blinded reviewers were needed to vote for eligibility. Eligibility was assessed through the first screening (title and abstract) and the second screening (full text) process. In each screening stage, two reviewers were involved in the decision-making process. Discrepancies were resolved through consensus, if necessary, with a third reviewer. To guide the decision-making process during the second screening, an Excel table was created to facilitate the process and to specify the location of the stimulus/response and whether the TCN was investigated.

### 2.7 Data extraction

Data were extracted in an Excel file developed specifically for this review, which was pilot-tested (with at least 10 papers) by the team members before the data extraction started. The Excel sheet had drop-down menus to maintain the consistency of the extracted data. We extracted the following information: “study characteristics”, *i.e.*, authors, design, publication details, sample size calculation, “characteristics of the population”, *i.e.*, type of animal (mouse, Rat, Guinea pig, Cats), age, gender, diagnosis, “stimulus details”, *i.e.*, type of stimulus, duration, location, location of response and “additional information about the area under study”, *i.e.*, TCN area studied, cervical area, “results”, *i.e.*, the direction of the connection at the TCN: from cranial, orofacial to cervical or *vice versa*, among others. Upon completing the data extraction, the information extracted from the studies was compiled into a Word file. Data extraction was carried out independently by one reviewer, and a second reviewer double-checked all data to ensure accuracy. Any disagreements on data extraction were resolved by a consensus meeting. Both reviewers received formal training to keep the consistency of the data extraction process.

### 2.8 Data charting and synthesis

A narrative description of the results was performed. In addition, evidence tables were used to compare study details, summarize findings and present results. Figures were used to display the results.

To determine the most predominant direction of connection between the orofacial, cranial, and cervical regions through the TCN, two independent investigators examined each of the included studies according to (1) the area where the stimulus was located, (2) the area of the TCN investigated (see Fig. 1) area where the response was observed. The areas of location were analyzed, and the direction of the connection was established. In case of disagreement between the two reviewers, consensus was sought or a third investigator made the decision.

## 3. Results

The database search resulted in a total of 1658 published articles. Based on the titles and abstracts, 315 articles were selected for the second screening and were read in full. After the screening process, eighty-three [15, 33, 34, 35, 36, 37, 38, 39, 40, 41, 42, 43, 44, 45, 46, 47, 48, 49, 50, 51, 52, 53, 54, 55, 56, 57, 58, 59, 60, 61, 62, 63, 64, 65, 66, 67, 68, 69, 70, 71, 72, 73, 74, 75, 76, 77, 78, 79, 80, 81, 82, 83, 84, 85, 86, 87, 88, 89, 90, 91, 92, 93, 94, 95, 96, 97, 98, 99, 100, 101, 102, 103, 104, 105, 106, 107, 108, 109, 110, 111, 112, 113, 114] articles were included, as described in the PRISMA flowchart (Fig. 2). The excluded studies and reasons are available in **Supplementary material 2**.

**Fig. 2.** PRISMA flow chart of included and excluded studies. TCN: trigeminocervical nucleus.

General characteristics of the included studies are provided in Table 2 and the details of the included studies are provided in **Supplementary material 3**.

**Table 2.** General characteristics of included studies (n = 83).

Study characteristic		n
Country		
	Japan	31
	USA	30
	Canada	8
	Others	14
Language		
	English	83
	Others	0
Published date		
	Before 2000	29
	Between 2000 and 2010	29
	After 2010	25
Ethical approval		
	Yes	55
	No	7
	Not reported	21
Animal Care registered		
	Yes	5
	No	22
	Not reported	55
	Not applicable	1
Funding		
	Not reported	14
	No funding	0
	Government	49
	Academic	13
	Others	13
Areas of Research (Journals)		
	Pain	19
	Brain Research/Neuroscience	36
	Neurology	15
	Dental Research	4
	Others	9
Population mammals		
	Rats	68
	Mice	1
	Cats	8
	Others	5
Humans		1
Sex		
	Mixed	9
	Female	6
	Male	46
	Not reported	22
Type of stimulus*		
	Chemical	39
	Mechanical	46
	Electrical	23
	Others	6
TCN area investigated*		
	Upper cervical spinal cord (C1/C2)	17
	Transition zone interpolaris/caudalis (Vi/Vc)	52
	Transition zone oralis/interpolaris (Vo/Vi)	9
	Other	26
	Not reported	4
Direction		
	Cervical to orofacial/cranial	5
	Orofacial/cranial to cervical	71
	Both	5
	Unclear	2

*Numbers do not add up since some studies are counted in more than one category. TCN: trigeminocervical nucleus.

### 3.1 General characteristics

From the 83 included studies, 31 studies were conducted in Japan (37%), 30 in the USA (36%), eight in Canada (10%) and 14 in other countries (17%). All studies were available and published in English language. From the included articles, 29 (35%) were published before 2000, 29 (35%) articles were published between 2000 and 2010 and 25 (30%) articles after 2010.

The population of included animal studies included rats in 68 studies (82%), cats in eight (10%) and other animals in five studies (6%). Only one study focused on humans [49]. The sex in the human study was mixed. In animal studies, the sex of the examined animals was male (n = 46; 55%), mixed (n = 9; 11%) or female (n = 6; 7%), and it was not reported in a great number of studies (n = 22; 26%).

Most of the analyzed studies were published in brain research and neuroscience areas (n = 36; 43%). See Table 2 for other characteristics.

### 3.2 TCN area investigated

About the TCN area investigated, 62.6% (n = 52) of the studies looked at the transition zone interpolaris/caudalis (Vi/Vc) (Table 2), 20.5% (n = 17) looked at the upper cervical spinal cord (C1/C2), followed by the transition zone oralis/interpolaris (Vo/Vi) in 10.8% (n = 9) of them. Twenty-six studies (31.3%) investigated other TCN areas, and four (4.8%) studies did not report the specific area investigated. For a detailed description of included studies regarding the connection between the cranial, orofacial and cervical regions through the TCN, please see **Supplementary material 4**. Fig. 3 shows a visual representation of the connections found.

**Fig. 3.** Visual representation in an anatomical map of the connections found from the analyzed studies. TCN: trigeminocervical nucleus; TMJ: Temporomandibular joint; ION: Inferior Orbital Nerve (Source: own illustration. Concept, idea of picture shared by Susan Armijo-Olivo and Harry von Piekartz).

### 3.3 Type of stimulus

Regarding the type of stimulus, 55.4% of the studies (n = 46) used a mechanical stimulus, followed by chemical (47%, n = 39), electrical (27.7%, n = 23), among others (7.3%, n = 6). In 65% of the studies (n = 54) “tracers” were used to demonstrate immunohistochemistry changes in the examined regions of the individual studies. These tracers included horseradish peroxidase, c-fos or phosphor-extracellular-signal regulated kinases (pERK).

For example, in the study of Adachi *et al*. [46] (2010) pERK or also known as phosphorylated ERK was used to assess changes in the immunohistochemistry by measuring the numbers of pERK cells in different locations of the brainstem after injections of an Adenosine triphosphate (ATP)-binding substance and a placebo (saline-injection) to the molar tooth pulp of rats.

In the study of Bereiter *et al*. [48] (2001) c-fos was used to make neuronal activity in the lower brainstem visible after a vagotomy in rats. See **Supplementary material 4** for details of the type of stimulus.

### 3.4 Direction

Regarding the direction, the majority of the studies included (85%, n = 71) reported orofacial-cranial-to-cervical region as the main direction investigated, five studies (6%) reported that both directions of connections were present, and in five (6%) studies reported cervical-to-orofacial-cranial region as the main direction investigated. Furthermore, two (2%) studies had unclear results related to the direction of the connection. See **Supplementary material 4** for details of the direction of connections in the included studies. **Supplementary material 5** provides an overall summary of the connections between the orofacial, cranial and cervical regions through the TCN and directions.

## 4. Discussion

### 4.1 Main results

This review aimed to determine whether there was a dominant direction on the connection of nociceptive activity between the cranial, orofacial and cervical regions through the TCN, *i.e.*, from cranial-orofacial-to-cervical region or *vice versa* in terms of anatomical, histological, biochemical, electrophysiological, neurophysiological, radiological and clinical outcomes in mammals and humans. The main findings of the present study showed that the direction orofacial-cranial-to-cervical region through the TCN was dominant or more investigated among the included studies looking at the connections among these regions. It is important to highlight that the majority of studies have been carried out in animals, and only one was conducted in humans, which limits the generalizability of the results. In addition, it should be mentioned that the included studies usually examined only one direction, typically from the orofacial-cranial-to-cervical region, because the investigators did not intend to examine both directions. In addition, a great number of included studies were not designed to investigate any direction of connection between the orofacial, cranial and cervical regions; rather, these studies were planned with other objectives. Thus, the direction was only reported as a secondary outcome for these studies. Therefore, caution is needed to interpret these findings and transfer them to clinical settings.

### 4.2 Heterogeneity of studies

A substantial heterogeneity was observed in the studies included regarding the selection of animals, the stimuli used, responses to these stimuli, different designs, outcomes and their effects on the results, which will be discussed below. Although a large number of studies included rats (n = 68, 82%), cats (n = 8, 10%), and other animals (n = 5, 6%) were used, which could explain at least in part the heterogeneity of the results found. The use of different mammal species in studies used for a review can indeed affect the results, and this variability arises from several factors. It is essential to consider these factors when reviewing or interpreting the results of such studies. Different mammal species have distinct physiological, genetic and metabolic characteristics. These variations can lead to differences in how they respond to treatments, diseases, or experimental conditions. For example, how a medication affects a mouse may not be directly applicable to its effects on a human due to differences in drug metabolism, receptor expression, and other biological factors [115]. Mammals vary in how they absorb, distribute, metabolize and excrete drugs and other substances. These differences can lead to variations in drug efficacy and toxicity between species. This is a critical consideration when conducting preclinical drug testing or toxicology studies [116]. For example, Adachi *et al*. [46] (2010), investigated the effect of an ATP receptor agonist in rats, or in the study of Classey *et al*. [35] (2001), the effect of an amino acid blocker was investigated. This shows the complexity of the included studies and always calls for caution concerning an interpretation of a clinical population.

Regarding the type of stimulus, mechanical stimuli were used in more than half of the studies, followed by chemical and electrical stimuli. It should be noted that some studies used multiple stimuli. The type of stimulus could, of course, affect the results, as it is known that different input modalities can trigger distinct biological responses. For example, chemical stimuli may activate specific signaling pathways in cells, whereas electrical stimulation may affect ion channel activity, and mechanical forces can induce structural changes [117]. These unique responses can lead to different experimental outcomes [117]. Concerning the stimuli investigated, no differences were detected between the different stimuli used in terms of a positive or negative connection or the direction studied. Thus, although several stimuli were investigated, no pattern regarding the type of stimulus was observed in the results. It also has to be considered that different tissues and organs may respond differently to various input modalities [118] as well as the timing and duration of input modalities can vary, which can affect the observed effects. Chemical signals may have rapid or delayed responses, while electrical impulses can occur in milliseconds. Researchers must also consider the temporal dynamics when interpreting results [119].

Regarding the authors’ fields of work or the journals in which the studies were included, it appears that a large proportion (n = 36, 44%) were published in brain research or neuroscientific journals, followed by pain (n = 19, 23%) and neurological (n = 15, 18%) journals. This shows that most of the included studies were published in non-specialty journals and may partly explain why the authors did not link their results to clinical populations.

### 4.3 Comparison with other reviews

The work of Armijo-Olivo *et al*. [20] (2006) evaluated the anatomical, biomechanical, and neurophysiological relationship between the orofacial region (stomatognathic system) and the cervical spine. This review hypothesizes that cranial and cervical structures influence each other and that craniofacial pain can be caused by all structures innervated by the three superior cervical nerves [20], which is in line with the results of our present review. The present review also supports the hypothesis of Haldeman and Dagenais (2001) [22], who focused on the literature on cervicogenic headaches. The authors stated that any pathology of the cervical spine can project symptoms to the head due to the mechanism of convergence in the TCN. Two studies included [52, 93] in the present review also examined the TCN regarding headaches and brought similar conclusions. Browne* et al*. [21] (1998) investigated in their review the relationship between craniofacial and craniocervical pain. Evidence of a relationship was provided by clinical studies, suggesting a predominant cervical-to-orofacial sensitization (cervical to the orofacial region). The rationale for a relationship between the craniofacial and craniocervical regions was provided by a pain reference mechanism based on the convergence theory in the TCN [20]. A closer look at this review does not necessarily reveal a contradiction with our results. Browne *et al*. [21] (1998) obtained the underlying data from clinical reports of patients with TMD. From all reports included, only one supported a direction from orofacial to cervical. Their conclusion supporting a dominant cervical to orofacial direction is based on the little available literature on the different directions in humans. As these authors were only looking for human pain studies this does not contradict the findings of this work but explains the difference.

Piovesan *et al*. [24] (2003) investigated the convergence mechanism itself. The convergence of trigeminal and superior cervical spinal nerves in the TCN has been held responsible for the nociceptive overlap between the orofacial and cervical regions in terms of immunohistochemical evidence, metabolic changes, electrophysiological nerve stimulation, clinical studies and pharmacological studies. For example, they cited the study of Kaube *et al*. [62] (1993) where immunohistochemical examination revealed fos-positive cells in lamina I and II of the medullary dorsal horn of the upper cervical spinal cord after stimulation of the superior sagittal sinus (SSS). The results of the present work can be confirmed as well. No further dimension could be added to the findings of Ashkenazi and Levin (2007) [23], as they evaluated the effect of greater occipital nerve blocks (GON block) on migraine, TTH, cervicogenic and cluster headaches which was only of secondary importance in the broader topic of the present research.

### 4.4 Strengths and limitations of this review

This scoping review was performed following high standards and aimed to capture the breadth of this literature, as well as the relevance of this topic [120]. The strengths and limitations in the implementation of the work are complex. One major strength is the concreteness and simplicity of the question. The goal of this review was to focus on the direction of the connection between regions and precisely describe the regions investigated and the path of connection. This makes the present work stand out from other reviews that have dealt with the trigeminocervical nucleus since most of the previous reviews did not focus on a very specific question, did not map the structures involved, and did not identify the dominant direction. The strict methodological approach already described is also one of the great strengths of this review.

One of the important outcomes of this review was the creation of an anatomical map through the information provided by the studies. Clinicians could use this anatomical map (Fig. 3) to track structures and pathways involved in connecting these different regions (see implications for practice below).

Due to the great number, variability and complexity of the studies included, there is always the danger of losing granularity in a scoping review. However, this type of review, as the name indicates, is designed to provide a general overview of the existing literature and map studies, but it does not give details about specific studies. However, it provides areas where gaps are identified and can be further investigated in future studies (as indicated below).

### 4.5 Implications for research

The majority of the studies examined the orofacial-cranial to cervical connection through the TCN. In these cases, the nociceptive input is given in the orofacial-cranial region, and the output is measured in the lower brainstem and/or cervical spine. Due to this one-sided approach, no conclusive assessment can be given as to which sensory directions are used more frequently since both directions are not examined with equal frequency in the available literature. Thus, it is unclear whether the dominant direction found in this review was because researchers were more interested in studying it or because it was the dominant direction. Future scientific studies on this topic could now focus on more practical-based outcomes that can provide increased guidance for treatment settings. In this context, it would be interesting to look for studies that investigate both directions and whether it can be determined whether one of the two directions could have a more predominant role in the overlapping symptoms in patients with symptoms in these regions. Clinical studies on humans would also be interesting. However, they have to be carefully designed since the methodologies used in animals cannot be used with humans, especially for obvious ethical reasons. For example, the activation of the TCN could be investigated in humans by using functional magnetic resonance imaging (fMRI). Another approach could be to focus less on the anatomy of the TCN and more on the clinical implications of connections present at the TCN.

### 4.6 Implications for clinical practice

The anatomical connection between the orofacial, cranial and cervical regions has been extensively explored and has been shown as an important relationship to be considered when planning and implementing management for patients with orofacial pain, headaches and cervical pain complaints. However, over the past twenty years, there has been a focus on studying the neurophysiological interactions between these two regions, particularly those involving the TCN. This research has provided a compelling rationale for incorporating the management of neck dysfunction and pain into the treatment of cranial and orofacial pain and *vice versa*. But until nowadays, the nature of this connection, and especially the direction of this connection, has not been totally clear. So, this review brings further evidence that although there is no one direction that is superior to the other, the connection between the orofacial and cervical regions via the TCN exists and is bidirectional; that is, either stimuli from the neck or the orofacial regions can be integrated at the level of the TCN and provide responses in either region. Therefore, the TCN is a crucial piece in the puzzle of understanding the relationship between these areas. It is strongly recommended clinically that all regions be considered in the evaluation and management of individuals with orofacial, cranial, and/or cervical dysfunction and pain. Also, clinicians should be aware of the variety of musculoskeletal tissues to trigger referred pain sensations among these regions and be knowledgeable about the typical spreading and referral patterns. In addition, this anatomical map could help to identify which areas could be triggered and which areas could respond to noxious stimuli at different locations based on these connections and our results. This can help clinicians explore some anatomical areas in clinical populations (clinical anatomical map; see Fig. 3) based on the results obtained by this review.

## 5. Conclusion

The main findings of the present study showed that orofacial, cranial and cervical regions are connected through the TCN, and this connection is bidirectional (both directions were investigated). However, the orofacial-cranial to cervical direction was dominant or most investigated by the included studies. However, this could only be due to the interest of researchers and does not necessarily determine that this direction has more influence in the overlapping symptoms seen in patients. The results of this review align with previous clinical studies investigating the interrelation between the orofacial, cranial, and cervical regions, supporting the neurophysiological model of bidirectional connections between these areas through the TCN. This alignment may aid in advancing more targeted experimental and clinical research in this field. Also, the results of this review could help clinicians to identify possible structures involved in these connections as well as the pathways followed. This, in turn, could facilitate appropriate clinical reasoning when managing patients with orofacial pain, headaches and neck pain.

## Supplementary Material

Supplementary material associated with this article can be found, in the online version, at https://files.jofph.com/ files/article/1966334253703544832/attachment/ Supplementary%20material.zip.










