# Supplementary material for: Integration of nociceptive activity from orofacial, cranial and cervical regions in the trigeminocervical nucleus: a scoping review with clinical implications
Source: J Oral Facial Pain Headache. 2025 Sep 12;39(3):1–12. doi: 10.22514/jofph.2025.042 (PMC12531578; doi:10.22514/jofph.2025.042)
Supplement: Supplementary file 1 [file Supplementary-material-1.docx]

Supplementary material

Supplementary material 1. Search strategies.

**Ovid MEDLINE(R) ALL <1946 to 22 March 2023>**

Results: 331

Date: 23 March 2023

1. (trigemin* adj5 (nucleus or tract)).mp. 4885

2. pars caudalis.mp. 177

3. subnucleus caudalis.mp. 565

4. (nociceptive adj5 convergent adj5 second-order neurons).mp. 0

5. (dorsal adj3 column* adj3 “C1-C3”).mp. 1

6. medullary dorsal horn.mp. 348

7. or/1-6 5359

8. exp Cervical Vertebrae/ 43,621

9. Neck/ 33,104

10. (cervical* or craniocervical or neck or atlantoaxial or atlantooccipital or zygapophysial or suboccipital or prevertebral or cervicogenic).mp. 558,986

11. ((capitis or longus colli or rectus posterior or obliquus posterior or sternocleidomastoideus or trapezius or semispinalis cervicis or multifidus) adj5 (M or musc*)).mp. 5165

12. ((alar or transversum) adj5 (l or lig or ligament*)).mp. 268

13. or/8–12 562,393

14. exp Face/ or Head/ 194,951

15. exp Temporomandibular Joint Disorders/ or exp Temporomandibular Joint/ 26,571

16. (Craniofacial or orofacial or head or face or facial or orbicularis or forehead or occipital or occiput or viscerocranium or cranium or skull or masticatory muscles or temporomandibular or TMJ or jaw or tongue or mouth or tooth or teeth).mp. 1,417,320

17. exp Masticatory Muscles/ 13,679

18. exp Skull/ 212,482

19. ((Masseter or temporalis or pterygoidalis medialis or pterygoideus lateralis or mylohyoideus or digastricus anterior) adj5 (M or musc*)).mp. 9138

20. or/14–19 1,566,739

21. 7 and 13 and 20 331

**Embase <1974 to 22 March 2023> (OVID interfac**e**)**

Results: 443

Date searched: 23 March 2023

1. (trigemin* adj5 (nucleus or tract)).mp. 6933

2. pars caudalis.mp. 185

3. subnucleus caudalis.mp. 616

4. (nociceptive adj5 convergent adj5 second-order neurons).mp. 0

5. (dorsal adj3 column* adj3 “C1-C3”).mp. 0

6. medullary dorsal horn.mp. 398

7. exp trigeminal nucleus/ 4532

8. or/1–7 7386

9. exp cervical vertebra/ 6722

10. neck/ or neck muscle/ 78,095

11. (cervical* or craniocervical or neck or atlantoaxial or atlantooccipital or zygapophysial or zygapophyseal or apophyseal or Z-joints or suboccipital or prevertebral or cervicogenic).mp. 766,141

12. ((capitis or longus colli or rectus posterior or obliquus posterior or sternocleidomastoideus or trapezius or semispinalis cervicis or multifidus) adj5 (M or musc*)).mp. 9850

13. ((alar or transversum) adj5 (l or lig or ligament*)).mp. 350

14. or/9–13 771,922

15. exp face/ or head/ 156,443

16. temporomandibular joint disorder/ or exp temporomandibular joint/ 27,204

17. (Craniofacial or orofacial or head or face or facial or orbicularis or forehead or occipital or occiput or viscerocranium or cranium or skull or masticatory muscles or temporomandibular or TMJ or jaw or tongue or mouth or tooth or teeth).mp. 1,949,361

18. exp masticatory muscle/ or head muscle/ or jaw muscle/ 17,762

19. exp skull/ 243,425

20. ((Masseter or temporalis or pterygoidalis medialis or pterygoideus lateralis or mylohyoideus or digastricus anterior) adj5 (M or musc*)).mp. 12,841

21. or/15–20 2,056,350

22. 8 and 14 and 21 443

**APA PsycInfo <1806 to March Week 2 2023> (OVID interface)**

Results: 65

Date searched: 23 March 2023

1. (trigemin* adj5 (nucleus or tract)).mp. 898

2. pars caudalis.mp. 20

3. subnucleus caudalis.mp. 120

4. (nociceptive adj5 convergent adj5 second-order neurons).mp. 0

5. (dorsal adj3 column* adj3 “C1-C3”).mp. 0

6. medullary dorsal horn.mp. 88

7. or/1-6 1024

8. “neck (anatomy)”/ 1365

9. (cervical* or craniocervical or neck or atlantoaxial or atlantooccipital or zygapophysial or zygapophyseal or apophyseal or Z-joints or suboccipital or prevertebral or cervicogenic).mp. 16,923

10. ((capitis or longus colli or rectus posterior or obliquus posterior or sternocleidomastoideus or trapezius or semispinalis cervicis or multifidus) adj5 (M or musc*)).mp. 494

11. ((alar or transversum) adj5 (l or lig or ligament*)).mp. 3

12. or/8–11 17187

13. exp “face (anatomy)”/ 2211

14. “head (anatomy)”/ 2138

15. (Craniofacial or orofacial or head or face or facial or orbicularis or forehead or occipital or occiput or viscerocranium or cranium or skull or masticatory muscles or temporomandibular or TMJ or jaw or tongue or mouth or tooth or teeth).mp. 232,235

16. masticatory muscles/ 151

17. skull/ 452

18. ((Masseter or temporalis or pterygoidalis medialis or pterygoideus lateralis or mylohyoideus or digastricus anterior) adj5 (M or musc*)).mp. 603

19. or/13–18 232,404

20. 7 and 12 and 19 65

**CINAHL Plus with Full Text (EBSCOhost interface)**

Results: 59

Date searched: 23 March 2023

Search mode: boolean/phrase

Deselect: apply equivalent subjects

S1. ((trigemin* N5 (nucleus or tract)) OR pars-caudalis OR subnucleus-caudalis OR medullary-dorsal-horn)

S2. (MH “Cervical Vertebrae+”) OR (MH “Neck”) OR (MH “Neck Muscles+”) OR cervical* or craniocervical or neck or atlantoaxial or atlantooccipital or zygapophysial or zygapophyseal or apophyseal or Z-joints or suboccipital or prevertebral or cervicogenic OR ((capitis or longus colli or rectus posterior or obliquus posterior or sternocleidomastoideus or trapezius or semispinalis cervicis or multifidus) N5 (M or musc*)) OR ((alar or transversum) N5 (l or lig or ligament*))

S3. (MH “Masticatory Muscles+”) OR (MH “Skull+”) OR (MH “Face+”) OR (MH “Head”) OR (MH “Temporomandibular Joint Diseases+”) OR (MH “Temporomandibular Joint”) OR Craniofacial or orofacial or head or face or facial or orbicularis or forehead or occipital or occiput or viscerocranium or cranium or skull or masticatory muscles or temporomandibular or TMJ or jaw or tongue or mouth or tooth or teeth or ((Masseter or temporalis or pterygoidalis medialis or pterygoideus lateralis or mylohyoideus or digastricus anterior) N5 (M or musc*))

S4. S1 AND S2 AND S3.

**Web of Science** (Indexes= (Science Citation Index Expanded (SCI-EXPANDED), Social Sciences Citation Index (SSCI), Arts & Humanities Citation Index (A&HCI), Conference Proceedings Citation Index—Science (CPCI-S), Conference Proceedings Citation Index—Social Science & Humanities (CPCI-SSH), Emerging Sources Citation Index (ESCI)) Timespan = All years)

Results: 321

Date searched: 23 March 2023

#1 TS = ((trigemin* NEAR/5 (nucleus or tract)) OR pars-caudalis OR subnucleus-caudalis OR medullary-dorsal-horn)

#2 TS = (cervical* or craniocervical or neck or atlantoaxial or atlantooccipital or zygapophysial or zygapophyseal or apophyseal or Z-joints or suboccipital or prevertebral or cervicogenic OR ((capitis or longus-colli or rectus-posterior or obliquus-posterior or sternocleidomastoideus or trapezius or semispinalis-cervicis or multifidus) NEAR/5 (M or musc*)) OR ((alar or transversum) NEAR/5 (l or lig or ligament*)))

#3 TS = (Craniofacial or orofacial or head or face or facial or orbicularis or forehead or occipital or occiput or viscerocranium or cranium or skull or masticatory-muscle* or temporomandibular or TMJ or jaw or tongue or mouth or tooth or teeth or ((Masseter or temporalis or pterygoidalis-medialis or pterygoideus-lateralis or mylohyoideus or digastricus-anterior) NEAR/5 (M or musc*)))

#4 #1 AND #2 AND #3.

**Scopus (Advanced Search)**

Results: 439

Date searched: 23 March 2023

(TITLE-ABS-KEY ((trigemin* W/5 (nucleus OR tract)) OR pars-caudalis OR subnucleus-caudalis OR medullary-dorsal-horn)) AND ( TITLE-ABS-KEY (cervical* OR craniocervical OR neck OR atlantoaxial OR atlantooccipital OR zygapophysial OR zygapophyseal OR apophyseal OR z-joints OR suboccipital OR prevertebral OR cervicogenic OR ((capitis OR longus-colli OR rectus-posterior OR obliquus-posterior OR sternocleidomastoideus OR trapezius OR semispinalis-cervicis OR multifidus) W/5 (m OR musc*)) OR ((alar OR transversum) W/5 (l OR lig OR ligament*)))) AND (TITLE-ABS-KEY (craniofacial OR orofacial OR head OR face OR facial OR orbicularis OR forehead OR occipital OR occiput OR viscerocranium OR cranium OR skull OR masticatory-muscle* OR temporomandibular OR tmj OR jaw OR tongue OR mouth OR tooth OR teeth OR ((masseter OR temporalis OR pterygoidalis-medialis OR pterygoideus-lateralis OR mylohyoideus OR digastricus-anterior) W/5 (m OR musc*))))
