# Supplementary material for: Integration of nociceptive activity from orofacial, cranial and cervical regions in the trigeminocervical nucleus: a scoping review with clinical implications
Source: J Oral Facial Pain Headache. 2025 Sep 12;39(3):1–12. doi: 10.22514/jofph.2025.042 (PMC12531578; doi:10.22514/jofph.2025.042)
Supplement: Supplementary file 2 [file Supplementary-material-2.docx]

Supplementary material

Supplementary material 2. Excluded studies.

| Title | Author | Year | Journal | Exclusion Reason |
| --- | --- | --- | --- | --- |
| Antinociceptive reflex alteration in acute posttraumatic headache following whiplash injury | Keidel *et al.* | 2010 | Pain | trigeminocervical complex (TCN) not investigated |
| Acute effect of created muscle fatigue in cervical region on temporomandibular joint in healthy | Kilinc *et al.* | 2015 | Turkish Journal of Physiotherapy Rehabilitation-Fizyoterapi Rehabilitasyon | TCN not investigated |
| Occipital afferent activation of second order neurons in the trigeminocervical complex in rat | LeDoare *et al.* | 2006 | Neuroscience Letters | orofacial region not investigated |
| Systemic administration of lidocaine suppresses the excitability of rat cervical dorsal horn neurons and tooth-pulp-evoked jaw-opening reflex | Takeda *et al.* | 2009 | European Journal of Pain | TCN not investigated |
| Does treatment of cervical dystonia reduce migraine frequency? | Bobker *et al.* | 2019 | Headache | TCN not investigated |
| Resolution of primary cervicogenic headache with sub-perception active recharge spinal cord stimulation (10721) | Yearwood *et al.* | 2016 | Neuromodulation | Abstract |
| The trapezius - Clinical & conditioning controversies | Wallden | 2014 | Journal of Bodywork and Movement Therapies | Letter to editor |
| Functional somatotopic organisation of motoneurons supplying the rabbit masseter muscle | Weijs | 1996 | Journal of Comparative Neurology | Cervical region not investigated |
| Phenol injection into cisterna magna for relief of advanced intractable cancer pain in the faciocephalyc area | Bortoluzzi *et al.* | 1986 | Journal of Neurosurgical Sciences | TCN not investigated |
| Extracranial projections of meningeal afferents and their impact on meningeal nociception and headache | Schuler *et al.* | 2013 | Neurocephalgia | Conference abstract |
| Trigeminal nociceptive neurons in the subnucleus reticularis-ventralis .1. response properties and afferent connections | Yokota *et al.* | 1991 | Neuroscience Research | The connection between cervical complex (CC) and Orofacial region is not investigated |
| Stimulation of the greater occipital nerve increases metabolic activity in the trigeminal nucleus caudalis and cervical dorsal horn of the cat | Goadsby *et al.* | 1997 | Pain | Orofacial region not investigated |
| Red forehead dot syndrome and migraine | Sethi *et al.* | 2007 | The Journal of Headache and Pain | Letter to editor |
| Sensitization of trigeminal nociceptive neurons in response to prolonged neck muscle pain: implications for migraine pathology | Durham *et al.* | 2012 | Headache | Conference abstract |
| The use of an odorant trigger to induce nociception in animals with trigeminal sensitization elicited by neck muscle inflammation: a novel model of migraine pathology | Durham *et al.* | 2017 | Headache | Conference abstract |
| Facial sensory disturbance associated with cervical myelopathy | Hagiwara *et al.* | 2017 | Journal of Neurological Sciences | Conference abstract |
| Phosphorylation of extracellular signal-regulated kinase in trigeminal spinal subnucleus caudalis neurons during regeneration of the trigeminal nerve | Hitomi *et al.* | 2010 | European Journal of Pain Supplements | Conference abstract |
| The effect of the PostureJac on deep cervical flexor endurance: implications in the management of cervicogenic headache and mechanical neck pain | Makofsky *et al.* | 2011 | Cranio | Targets not interest of study |
| Effect of the dental implant surgery on the pain threshold of head and neck region | Ishigaki *et al.* | 2011 | European Journal of Pain Supplements | Full-text not available |
| The results of trigeminal nucleotractotomy in facial pain | Grigorianlu *et al.* | 2000 | Zhurnal Voprosy Neirokhirurgii Imeni N - N - Burdenko | Abstract |
| Ramsey hunt syndrome with dysphagia with MRI evidence of brain stem and upper cervical cord involvement | Satyan *et al.* | 2012 | Neurology Conference | Conference abstract |
| Contribution of P2Y12 receptors to animal model of NTG-induced migraine-associated pain | Goloncser *et al.* | 2019 | Anales de la Real Academia Nacional de Farmacia | Conference abstract |
| Bilateral mechanical-pain sensitivity over the trigeminal region in patients with chronic mechanical neck pain | LaTouche *et al.* | 2010 | Journal of Pain | TCN not investigated |
| Central projections of cervical primary afferent fibers in the guinea pig: an HRP and WGA/HRP tracer study | Prihoda *et al.* | 1991 | Journal of Comparative Neurology | Orofacial region not investigated |
| Effects of acupuncture on prevention of migraine attacks-relationship between decrease in the number of headaches days and improvement of head and neck muscles tenderness | Yamaguchi *et al.* | 2013 | Journal of the Japanese Society of Balneology, Climatology & Physical Medicine | TCN not investigated |
| The trigeminal tract and nucleus procedures in treatment of atypical facial pain | Kanpolat *et al.* | 2005 | Surgical Neurology | The connection between CC and Orofacial region is not investigated |
| Ultrastructure and synaptology of the paratrigeminal nucleus in the rat: Primary pharyngeal and laryngeal afferent projections | Saxon *et al.* | 2006 | Synapse | Targets not interest of study |
| Antinociceptive activity of botulinum toxin type A in the rat trigeminal region | Matak *et al.* | 2015 | Toxicon | Full-text not available |
| Convergence of afferents from superior sagittal sinus and tooth-pulp on cells in the upper cervical spinal-cord of the cat | Angusleppan *et al.* | 1994 | Neuroscience letters | Cervical region not investigated |
| Value of stereotaxic mesencephalic tractotomy in neoplastic cervicofacial pain | Cohadon *et al.* | 1985 | Revue de Laryngologie Otologie Rhinologie | Abstract |
| The central projections of the great auricular nerve primary afferent fibers—an HRP transganglionic tracing method | Liu *et al.* | 1988 | Brain Research | Orofacial region not investigated |
| Dental malocclusion stimulates neuromuscular circuits associated with temporomandibular disorders | Liu *et al.* | 2018 | European Journal of Oral Sciences | TCN not investigated |
| Role of neuron and non-neuronal cell communication in persistent orofacial pain | Iwata *et al.* | 2019 | Journal of Dental Anesthesia & Pain Medicine | Review |
| Erratum to: “Greater occipital nerve block in chronic migraine” | Saracco *et al.* | 2011 | Neurological Sciences | Erratum |
| Effect of upper cervical spine manipulation on mouth opening | MansillaFerragud *et al.* | 2008 | Osteopatia Cientifica | TCN not investigated |
| Therapeutic blockade of greater occipital and supraorbital nerves in migraine patients | Caputti *et al.* | 1997 | Headache | TCN not investigated |
| Ultrasound-guided C2 nerve block as a treatment for trigemeninal neuralgia: A case report | Shivazad *et al.* | 2016 | Regional Anesthesia and Pain Medicine | Full-text not available |
| The blink reflex in chronic tension-type headache, migraine, and cervicogenic headache | Sand *et al.* | 1994 | Cephalgia | TCN not investigated |
| ERK-GluR1 phosphorylation in trigeminal spinal subnucleus caudalis neurons is involved in pain associated with dry tongue | Makaya *et al.* | 2016 | Molecular pain | Cervical region not investigated |
| Cervical central cord syndrome involving the spinal trigeminal nucleus: a case report | Chang *et al.* | 1995 | Surgical Neurology | TCN not investigated |
| Paresthesia-free relief: Higher frequency stimulation of high cervical leads for patients with chronic facial pain | Patel *et al.* | 2018 | Neuromodulation | Conference abstract |
| Trigeminal Neuralgia Resulting from Delayed Cervical Cord Compression after Acute Traumatic Fracture of Odontoid Process | Shim *et al.* | 2019 | Korean Journal of Neurotrauma | TCN not investigated |
| Light microscopic localization of calcitonin gene-related peptide in the normal feline trigeminal system and following retrogasserian rhizotomy | Henry *et al.* | 1996 | Comparative Neurology | Cervical region not incestigated |
| Neuronal Nitric Oxide Synthase is Involved in the Induction of Nerve Growth Factor-Induced Neck Muscle Nociception | Isaak *et al.* | 2011 | Headache | TCN not investigated |
| Pulsed radiofrequency of superior cervical sympathetic ganglion for treatment of refractory migraine | Carcamo | 2017 | Pain medicine | Letter to editor |
| Masseter silent period evoked by stimulating the median nerve, cervical plexus and mental nerve | Urban *et al.* | 1992 | Eeg-Emg-Zeitschrift Für Elektroenzephalographie Elektromyographie Und Verwandte Gebiete | The connection between CC and Orofacial region is not investigated |
| Activation of 5-HT1B/(1D) receptor in the periaqueductal gray inhibits nociception | Bartsch *et al.* | 2004 | Annals of Neurology | The connection between CC and Orofacial region is not investigated |
| Trigeminal herpes zoster with a long-segmental enhanced lesion of the spinal trigeminal nucleus and tract on magnetic resonance imaging | Ozawa *et al.* | 2017 | Journal of the Neurological Sciences | Abstract |
| Efferents from the lateral frontal cortex to spinomedullary target areas, trigeminal nuclei, and spinally projecting brainstem regions in the hedgehog tenrec | Kunzle *et al.* | 1996 | Comparative Neurology | Orofacial region not investigated |
| Extending the phenotype of FOSMN syndrome: An important ALS mimic | Broad *et al.* | 2014 | Amyotrophic Lateral Sclerosis and Frontotemporal Degeneration | Poster |
| Responses of trigeminal brain stem neurons and the digastric muscle to tooth-pulp stimulation in awake cats | Boissonade *et al.* | 1993 | Journal of Neurophysiology | Cervical Region not investigated |
| Differential terminal distribution of single large cutaneous afferent fibers in the spinal trigeminal nucleus and in the cervical spinal dorsal horn | Hayashi | 1982 | Brain Research | The connection between CC and Orofacial region is not investigated |
| Supraspinal inhibition of trigeminal input into subnucleus caudalis by dorsal column stimulation | Atweh | 1985 | Brain Research | The connection between CC and Orofacial region is not investigated |
| Advance on reasearch of cervicogenic headache | Zhang *et al.* | 2003 | Chinese Journal of Clinical Rehabilitation | Abstract |
| Facial pain relieved by dorsal root entry zone lesions in the trigeminal nucleus caudalis: report of two cases | Chen | 1993 | Journal of the Formosan Medical Association | Abstract |
| Simultaneous modulation of the exteroceptive suppression periods in the trapezius and temporalis muscles by experimental muscle pain | Ge *et al.* | 2004 | Clinical Neurophysiology | TCN not investigated |
| Upper cervical afferents to the motor trigeminal nucleus and the subnucleus oralis of the spinal trigeminal nucleus in the rat: an anterograde and retrograde tracing study | Xiong *et al.* | 2000 | Neuroscience Letters | The connection between CC and Orofacial region is not investigated |
| Non-nociceptive upper limb afferents modulate masseter muscle EMG ativity in man | Deriu *et al.* | 2002 | Experimental Brain Research | Targets not interest of study |
| Effect of cortical spreading depression on basal and evoked traffic in the trigeminovascular sensory system | Lambert *et al.* | 2011 | Cephalgia | Cervical region not investigated |
| Myofascial trigger points, neck mobility and forward head posture in unilateral migraine | Fernandez-de-las-Penas *et al.* | 2006 | Cephalgia | TCN not investigated |
| Concomitant mandibular and head-neck movements during jaw opening-closing in man | Eriksson *et al.* | 1998 | Journal of Oral Rehabilitation | TCN not investigated |
| Glial cell activation in the spinal-trigeminal system in vivo: Implications for basic mechanisms of pain transmission | Magni *et al.* | 2011 | Glia | Abstract |
| Osteopathic management of an adult patient suffering from trigeminal neuralgia after a postoperative Arnold Chiari type I decompression: Case report | Zegarra-Parodi *et al.* | 2010 | European Journal of Pain Supplements | Conference abstract |
| Nucleus Caudalis Stimulation for Facial Pain | Lau | 2022 | Neuromodulation | The connection between CC and Orofacial region is not investigated |
| The composition and central projections of the internal auricular nerves of the dog. | Chien *et al.* | 1996 | Journal of anatomy | The connection between CC and Orofacial region is not investigated |
| Migraine pathophysiology | Goadsby *et al.* | 2005 | Headache | Review |
| GABAB receptors in the NTS mediate the inhibitory effect of trigeminal nociceptive inputs on parasympathetic reflex vasodilation in the rat masseter muscle | Ishii *et al.* | 2012 | American Journal of Physiology-Regulatory Integrative & Comparative Physiology | TCN not investigated |
| Symptomatic trigeminal autonomic cephalalgia associated with allodynia in a patient with multiple sclerosis | Liu *et al.* | 2008 | Journal of the Chinese Medical Association | Cervical region not investigated |
| Neurobiology of migraine | Goadsby *et al.* | 2009 | Neuroscience | Review |
| Stereotactic trigeminal nucleotomy for dysesthetic facial pain | Piedimonte at al. | 1997 | Stereotactic & Functional Neurosurgery | Conference Abstract |
| On whether there is a direct sympathetic influence on jaw muscle spindles | Passatore *et al.* | 1981 | Brain Research | TCN not investigated |
| Experimental masseter muscle pain alters jaw-neck motor strategy | Wiesinger *et al.* | 2013 | European Journal of Pain | TCN not investigated |
| A trigeminoreticular pathway: implications in pain | Panneton *et al.* | 2011 | PLoS One | Targets not interest of study |
| Spinal cheiro-oral syndrome: a common neurological entity in an unusual site | Lin *et al.* | 2011 | Neurologia i Neurochirurgia Polska | TCN not investigated |
| Vascular origin of cranial sensory disturbances caused by pathology of the lower cervical spine | Keuter | 1971 | Acta Neurochirurgica | TCN not investigated |
| Excitatory amino release within spinal trigeminal nucleus after mustard oil injection into the temporomandibular joint region of the rat | Bereiter *et al.* | 1996 | Pain | Cervical region not investigated |
| Involvement of phosphorylated extracellular signal-regulated kinase in facial neuropathic pain following cervical nerve injury in rats | Kobayashi *et al.* | 2010 | European Journal of Pain Supplements | Abstract |
| Headaches associated with diseases of skull and neck | Gobel *et al.* | 1999 | Schmerz | Review |
| Chiropractic management of cluster-tic syndrome: a case report | Swain *et al.* | 2007 | Chiropractic Journal of Australia | TCN not investigated |
| Localization of Met-enkephalin-like immunoreactivity within pain-related nuclei of cervical spinal cord, brainstem and midbrain in the cat | Conrath-Verrier *et al.* | 1983 | Brain Research Bulletin | The connection between CC and Orofacial region is not investigated |
| Herpes zoster ophthalmicus caught in the (Tr)act! | Mufti *et al.* | 2018 | Emergency Radiology | Cervical region not investigated |
| Anatomic landmarks of the glossopharyngeal nerve: A microsurgical anatomic study [1] (multiple letters) | Tubbs *et al.* | 2004 | Neurosurgery | Letter to editor |
| Percutaneous computed tomography-guided radiofrequency ablation of upper spinal cord pain pathways for cancer-related pain | Raslan | 2008 | Neurosurgery | TCN not investigated |
| Neck motor unit activities induced by inputs from periodontal mechanoreceptors in rats | Zeredo *et al.* | 2002 | Journal of Dental Research | TCN not investigated |
| Migraine without aura, arthrogenic and myofascial cervical afferents: Role of EBM physiotherapy | Mourad | 2017 | Journal of Headache and Pain | Conference abstract |
| Facial sensory disturbance associated with cervical compressive myelopathy | Kasahata | 2016 | Medical Science | TCN not investigated |
| technique: MYOFASCIAL TECHNIQUES. Uncoupling the Neck and Jaw | Til *et al.* | 2017 | Massage & Bodywork | TCN not investigated |
| Basic research and clinical investigations of the neural basis of orofacial pain | Matsuka *et al.* | 2015 | Journal of Oral Biosciences | Review |
| Central mechanisms of masticatory muscle pain induced by occlusal interference | Yu *et al.* | 2007 | Hua Xi Kou Qiang Yi Xue Za Zhi | Cervical region not investigated |
| Central distribution of synaptic contacts of primary and secondary jaw muscle spindle afferents in the trigeminal motor nucleus of the cat | Kishimoto *et al.* | 1998 | Comparative Neurology | Targets not interest of study |
| Enhancement of ERK phosphorylation and photic responses in Vc/C1 neurons of a migraine model | Hitomi *et al.* | 2017 | Neuroscience letters | Cervical region not investigated |
| Bilateral greater occipital nerve block: A symptomatic treatment of the postdural puncture headache. A prospective, randomised trial | Lopez-Vizcayno | 2009 | European Journal of Anaesthesiology | Conference abstract |
| Painful Conditioning Stimuli of the Craniofacial Region Evokes Diffuse Noxious Inhibitory Controls in Men and Women | Wang *et al.* | 2010 | Journal of Orofacial Pain | TCN not investigated |
| A translational in vivo model of trigeminal autonomic cephalalgias: therapeutic characterization | Akerman *et al.* | 2012 | Brain | Cervical region not investigated |
| Extratrigeminal cluster headache | Sanin *et al.* | 1993 | Headache | TCN not investigated |
| Prepulse modulation and recovery of trigemino-cervical reflex in normal subjects | Sirinocak *et al.* | 2019 | Neurological Sciences | TCN not investigated |
| On the centripetal pathways which transmit the radiations and reflex phenomena anteriorly in visceral afflictions | Lapinsky | 1926 |  | The connection between CC and Orofacial region is not investigated |
| Tractotomy and partial vertical nucleotomy--for treatment of special forms of trigeminal neuralgia and cancer pain of face and neck | Plangger *et al.* | 1987 | Acta Neurochirurgica | Full-text not available |
| Role of medullary astroglial glutamine synthesis in tooth pulp hypersensitivity associated with frequent masseter muscle contraction | Watase *et al.* | 2018 | Molecular Pain | TCN not investigated |
| Glial cell activation in the spinal-trigeminal system in vivo: Implications for basic mechanisms of pain transmission | Magni *et al.* | 2011 | Glia | Full-text not available |
| Organization of projections from the spinal trigeminal subnucleus oralis to the spinal cord in the rat: a neuroanatomical substrate for reciprocal orofacial-cervical interactions | Devoize *et al.* | 2010 | Brain Research | Cervical region not investigated |
| Post traumatic headache and allodynia | Baruah *et al.* | 2009 | Cephalgia | Abstract |
| The neck and headaches | Bogduk | 2004 | Neurologic Clinics | Review |
| Restoring pain modulation for chronic migraine; By mild stimuli of stretching tender points of the neck | Saka *et al.* | 2013 | Cephalgia | Conference abstract |
| Intractable Facial Pain Relieved by Percutaneous Trigeminal Tractotomy | Fox | 1971 | JAMA | Brief commentary |
| Mechanical hyperalgesia profile and clinical features in subgroups of patients with temporomandibular disorders and patients with chronic neck pain | Fernanez-Carnero *et al.* | 2011 | European Journal of Pain Supplements | Conference abstract |
| Sustained response of new daily persistent headache to radiofrequency ablation of pericranial nerves | Fermo *et al.* | 2017 | Pain Medicine | Conference abstract |
| A blessing in disguise: resolution of tardive dyskinesia with development of cervical myelitis | Lim *et al.* | 2006 | Movement Disorders | TCN not investigated |
| Differential activation of ascending noxious pathways associated with trigeminal nerve injury | Okada *et al.* | 2019 | Pain | Cervical region not investigated |
| Inhibition of temporomandibular joint input to medullary dorsal horn neurons by 5HT3 receptor antagonist in female rats | Okamoto *et al.* | 2015 | Neuroscience | Cervical region not investigated |
| Effect of trigeminal tractotomy on dental sensation in humans | Young | 1982 | Journal of Neurosurgery | TCN not investigated |
| Occipital nerve stimulation in primary headaches: influence of cervical sensory afferences on the caudal trigeminal nucleus? | Straube *et al.* | 2010 | Fortschritte der Neurologie-Psychiatrie | Brief commentary |
| A Case of Prethoracic Pain Radiating Upward and Initiating Nervus Intermedius Neuralgia and Migraine Headache: Could Epicrania Fugax Pain Start in the Upper Body? | Wang *et al.* | 2017 | Journal of Oral & Facial Pain and Headache | TCN not investigated |
| Understanding migraine: Pathophysiology and presentation | Unger *et al.* | 2003 | Emergency Medicine | Review |
| Interneurones of the supratrigeminal area mediating reflex inhibition of trigeminal and facial motoneurones in the rat | Minkels *et al.* | 1995 | Archives of Oral Biology | Cervical region not investigated |
| Tractotomy and partial nucleotomy as a form of therapy in refractory pain of the trigeminal nerve and cancer pain in the head and neck area | Kostron *et al.* | 1990 | Wiener klinische Wochenschrift | Full-text not available |
| Evaluation and interpretation of the therapeutic effectiveness of the epicranial nerves blocks in migraine patients | Caputti *et al.* | 1999 | Confinia Cephalalgica | TCN not investigated |
| Generalized somatic dysesthesias induced by stimulation of teeth with cold water: An unreported form of brainstem integrative synesthesia | deAssisAquinoGondim  *et al.* | 2014 | Journal of Neurological Sciences | Letter to editor |
| Understanding cervicogenic headache | Chua *et al.* | 2012 | Anesthesiology & Pain Medicine | Brief commentary |
| The outcomes of interventional pain management for treatment of intractable headaches refractory to conventional medical managements | Taimoorazy | 2013 | Cephalalgia | Conference Abstract |
| Spinocerebellar projections in the pigeon with special reference to the neck region of the body | Necker | 2001 | Comparative Neurology | No mammals |
| Anesthetic blockade of the greater occipital nerve in migraine prophylaxis | Piovesan *et al.* | 2001 | Arquivos De Neuro-Psiquiatria | The connection between CC and Orofacial region is not investigated |
| TRPA1 antagonism in a pre-clinical model of migraine: Modulation of the inflammatory pathway | DeMartini *et al.* | 2019 | Neurological Sciences | Conference abstract |
| Comparison of masseter muscle referred sensations after mechanical and glutamate stimulation: a randomized, double-blind, controlled, cross-over study | Exposto *et al.* | 2018 | Pain | TCN not investigated |
| Dorsal mesencephalic projections to pons, medulla, and spinal cord in the cat: limbic and non-limbic components | Cowie *et al.* | 1992 | Comparative Neurology | The connection between CC and Orofacial region is not investigated |
| Somatotopy in the Medullary Dorsal Horn As a Basis for Orofacial Reflex Behavior | Panneton *et al.* | 2017 | Frontiers in Neurology | Targets not interest of study |
| The organization and activity patterns of the anterior and posterior heads of the guinea pig digastric muscle | Lev-Tov *et al.* | 1987 | Journal of Neurophysiology | Targets not interest of study |
| Effect of trigeminal tractotomy on behavioral response to dental pulp stimulation in the monkey | Young *et al.* | 1981 | Journal of Neurosurgery | The connection between CC and Orofacial region is not investigated |
| Stomach-Sleepers’ Headache | Dalton | 2015 | Massage & Bodywork | Abstract |
| The cervicosenic headache | Dvorak *et al.* | 1997 | Therapeutische Umschau | Review |
| An anatomical review of pain sensation processing in the trigeminal nerve of the head and neck region | Takahashi | 2007 | Neuro-Ophtalmology | Review |
| Peripheral and central distribution of fibres of the mesencephalic trigeminal root in the rat | Matesz | 2001 | Neuroscience Letters | TCN not investigated |
| Effect of the dental implant surgery on the pain threshold of head and neck region | Ishigaki | 2011 | European Journal of Pain Supplements | Poster |
| Ultrasound-guided C2 nerve block as a treatment for trigemeninal neuralgia: A case report | Shivazad *et al.* | 2015 | Regional Anesthesia and Pain Medicine | Conference Abstract |
| A comparison of pain control between occipital nerve block and occipital nerve radiofrequency; A retrospective cohort study | Pathak *et al.* | 2019 | Pain Medicine | Poster |
| Convergence of nociceptive information from temporomandibular joint and tooth pulp afferents on C1 spinal neurons in the rat | Nishikawa *et al.* | 2004 | Life Sciences | TCN not investigated |
| H3-HRP analysis of the nerve supply to primate teeth | Chiego *et al.* | 1983 | Journal of Dental Research | TCN not investigated |
| Innervation of rat and human dura mater and pericranial tissues by meningeal afferents | Schueler *et al.* | 2014 | Headache | Targets not interest of study |
| Altered Brainstem Pain Modulating Circuitry Functional Connectivity in Chronic Painful Temporomandibular Disorder | Mills *et al.* | 2021 | Journal of Pain | Cervical region not investigated |
| Trigeminal pathways for hypertonic saline- and light-evoked corneal reflexes | Rahman *et al.* | 2014 | Neuroscience | Cervical region not investigated |
| Integration in trigeminal premotor interneurones in the cat. 2. Functional characteristics of neurones in the subnucleus-gamma of the oral nucleus of the spinal trigeminal tract with a projection to the digastric motoneurone subnucleus | Olsson *et al.* | 1991 | Experimental Brain Research | Cervical region not investigated |
| Cervical spinal-cord neurons receiving sensory input from the cranial vasculature | Lambert *et al.* | 1991 | Cephalgia | TCN not investigated |
| Trigemino-cervical reflex in patients with headache | Milanov *et al.* | 2003 | Cephalgia | TCN not investigated |
| Neck muscle length modulates nociceptive reflex evoked by noxious irritant application to rat neck tissues | Shin *et al.* | 2005 | Experimental Brain Research | TCN not investigated |
| On the functional neuroanatomy of neck pain - Introduction | Goadsby *et al.* | 2008 | Cephalgia | Review |
| Anterior and posterior neck muscle activation during a variety of biting tasks | Hellmann *et al.* | 2012 | European Journal of Oral Sciences | TCN not investigated |
| The neck: A pain generator for the head | Chua *et al.* | 2012 | Pain Management | Review |
| Facial injections of pruritogens and algogens excite partly overlapping populations of primary and second-order trigeminal neurons in mice | Akiyama *et al.* | 2010 | Journal of Neurophysiology | The connection between CC and Orofacial region is not investigated |
| Corticotrigeminal motor pathway in the rat--II. Anterio- and retrograde HRP labeling | Ohta *et al.* | 1989 | Comparative Biochemistry & Physiology A-Comparative Physiology | Targets not interest of study |
| A case with trigeminal herpes zoster manifesting a long lesion of the spinal trigeminal nucleus and tract on MR T2-weighted image | Nagane *et al.* | 2001 | Clinical Neurology | Abstract |
| Degeneration of the primary snout sensory afferents in the cervical spinal cords following the infraorbital nerve transection in some mammals | Chang *et al.* | 1988 | Anatomischer Anzeiger | Not neurophysiological study |
| Acute injury of the neck: anatomical and pathological basis of pain | Taylor *et al.* | 1983 | Annals of the Academy of Medicine | Conference abstract |
| A comparative study of changes operated by sympathetic nervous system activation on spindle afferent discharge and on tonic vibration reflex in rabbit jaw muscles | Passatore *et al.* | 1996 | Journal of the Autonomic Nervous System | TCN not investigated |
| Bilateral greater orbital nerve block: Efficacy in postdural puncture headache | Nair | 2018 | Saudi Journal of Anaesthesia | Letter to editor |
| Opioid involvement in electromyographic (EMG) responses induced by injection of inflammatory irritant into deep neck tissues | Hu *et al.* | 1996 | Somatosensory and Motor Research | TCN not investigated |
| Cervical plexus block helps in diagnosis of orofacial pain originating from cervical structures | Shinozaki *et al.* | 2006 | Tohoku Journal of Experimental Medicine | TCN not investigated |
| Neuroanatomical principles of the craniomandibular system. The basal ganglia | Schupp *et al.* | 2016 | Manuelle Medizin | Targets not interest of study |
| Effects of sympathetic nerve stimulation on intra-oral mechanoreceptor activity in the cat | Cash *et al.* | 1982 | Journal of Physiology | TCN not investigated |
| Suboccipital injection for migraine prevention | KameshArun | 2015 | Cephalalgia | Abstract |
| Somatotopic organization of raccoon dorsal column nuclei | Johnson *et al.* | 1986 | Comparative Neurology | orofacial region not investigated |
| The trigeminal tract and nucleus procedures in treatment of atypical facial pain | Kanpolat *et al.* | 2005 | Surgical neurology | Cervical region not investigated |
| Trigeminal proprioceptive projections to the hypoglossal nucleus and the cervical ventral gray column | Mizuno *et al.* | 1970 | Comparative Neurology | TCN not investigated |
| Stimulation of the greater occipital nerve induces increased central excitability of dural afferent input | Bartsch *et al.* | 2002 | Brain | TCN not investigated |
| Tympanic Resonance Hypothesis | Boedts | 2020 | Frontiers in Neurology | Review |
| Central neuropathic pain: Multiple sclerosis-related headaches | Charlson *et al.* | 2015 | Case-based diagnosis and management of headache disorders | Book chapter |
| Association of neck pain with symptoms of temporomandibular dysfunction in the general adult population | Chiancaglini *et al.* | 1999 | Scandinavian Journal of Rehabilitation Medicine | TCN not investigated |
| Immediate effects of manual therapy targeting the cervical or orofacial region in neck symptoms in patients with myofascial temporomandibular pain | Salom-Moreno *et al.* | 2016 | Manual Therapy | Conference abstract |
| C1 retrograde spinal cord stimulation for trigeminal deafferentation pain. case study with long-term follow-up | Richter | 2017 | Neuromodulation | Conference abstract |
| Referred pain after painful stimulation of the greater occipital nerve in humans: Evidence of convergence of cervical afferences on trigeminal nuclei | Piovesan *et al.* | 2001 | Cephalalgia | TCN not investigated |
| Facial neuralgia and the cervical vertebral column (author’s transl) | Tilscher | 1978 | Münchener Medizinischer Wochenschrift | Brief commentary |
| Behavioral characteristics and c-Fos expression in the medullary dorsal horn in a rat model for orofacial cancer pain | Ono *et al.* | 2009 | European Journal of Pain | Cervical region not investigated |
| An experimental model of hyper-irritability in the trigeminal skin field of the rat | Sakai *et al.* | 1979 | Pain | The connection between CC and Orofacial region is not investigated |
| Studies on pain conduction in the trigeminal nerve. A contribution to the surgical treatment of facial pain | Sjoqvist | 1938 | Acta Psychiatrica et Neurologica | Abstract |
| EHMTI-0009. A particular case of cluster headache | Tertan *et al.* | 2014 | Journal of Headache and Pain | Abstract |
| Suboccipital neurostimulation in medically intractable chronic daily headache | Bartsch | 2008 | The Lancet Neurology | Brief commentary |
| Neuron-glia interaction is a key mechanism underlying persistent orofacial pain | Iwata *et al.* | 2017 | Journal of Oral Sciences | Review |
| Onion-skin Hemifacial Dysesthesia Successfully Treated with C2-4 Anterior Cervical Decompression and Fusion: A Case Report | Kuraishi *et al.* | 2016 | Nmc Case Report Journal | TCN not investigated |
| Functional organization of trigeminal subnucleus interpolaris: nociceptive and innocuous afferent inputs, projections to thalamus, cerebellum, and spinal cord, and descending modulation from periaqueductal gray | Hayashi *et al.* | 1984 | Journal of Neurophysiology | TCN not investigated |
| Peripheral and Central Mechanisms of Persistent Orofacial Pain | Shinoda *et al.* | 2019 | Frontiers in Neuroscience | Review |
| Efferent connections of the brainstem trigeminal complex with the facial nucleus of the rat | Erzurumlu *et al.* | 1979 | Comparative Neurology | The connection between CC and Orofacial region is not investigated |
| Reappraisal of somatotopic tactile representation within trigeminal subnucleus caudalis | Yokota *et al.* | 1980 | Journal of Neurophysiology | The connection between CC and Orofacial region is not investigated |
| Mesencephalic trigeminal nucleus neurons supplying the jaw closing muscles have no spinal projection: a fluorescent double-labeling study in birds and mammals | Lucchi *et al.* | 1997 | Anatomical Record | TCN not investigated |
| Severe headache with eye involvement from herpes zoster ophthalmicus, trigeminal tract, and brainstem nuclei | Siritho | 2015 | Case Reports in Radiology | Cervical region not investigated |
| Massaging over the greater occipital nerve reduces the intensity of migraine attacks: evidence for inhibitory trigemino-cervical convergence mechanisms | Piovesan | 2007 | Arquivos de Neuro-Psiquiatria | TCN not investigated |
| Central sensitization induced in trigeminal and upper cervical dorsal horn neurons by noxious stimulation of deep cervical paraspinal tissues in rats with minimal surgical trauma | Vernon *et al.* | 2009 | Journal of Manipulative & Physiological Therapeutics | Orofacial region not investigated |
| Cluster-like headache: Association with cervical syringomyelia and Arnold-Chiari malformation | Seijo-Martinez *et al.* | 2004 | Cephalalgia | Orofacial region not investigated |
| Trigeminal neuralgia and chiropractic care: a case report | Rodine *et al.* | 2010 | Journal of the Canadian Chiropractic Association | TCN not investigated |
| Vertical dimension. Part 2: The changes in electrical activity of the cervical muscles upon varying the vertical dimension | Miralles *et al.* | 2002 | Cranio-the Journal of Craniomandibular & Sleep Practice | TCN not investigated |
| Editorial: Mechanisms of Orofacial Pain and Sex Differences | Liu *et al.* | 2021 | Frontiers in Integrated Neuroscience | Cervical region not investigated |
| A case of recurrent myelitis associated with anti-myelin oligodendrocyte glycoprotein antibody that developed only as localized short spinal cord lesions | Matsumoto *et al.* | 2017 | Rinsho Shinkeigaku - Clinical Neurology | The connection between CC and Orofacial region is not investigated |
| Projections of mechanoreceptive fields to cuneate-gracile and spinal trigeminal nuclear regions in sheep | Woudenberg | 1970 | Brain Research | The connection between CC and Orofacial region is not investigated |
| A dual effect of sympathetic nerve stimulation on jaw muscle spindles | Passatore *et al.* | 1982 | Journal of Autonomic Nervous System | TCN not investigated |
| Involvement of trigeminal subnucleus caudalis (medullary dorsal horn) in craniofacial nociceptive reflex activity | Tsai *et al.* | 1999 | Pain | Cervical region not investigated |
| Comparison of the effects of manipulation of the craniocervical joint and thoracic spine on interincisal distance | Baillergeau *et al.* | 2012 | Kinesitherapie | TCN not investigated |
| The Role of the Trigemino Cervical Complex in Chronic Whiplash Associated Headache: A Cross Sectional Study | Watson *et al.* | 2016 | Headache | TCN not investigated |
| The greater occipital nerve and its spinal and brainstem afferent projections: A stereological and tract-tracing study in the rat | Garcia-Magro | 2018 | Comparative Neurology | Orofacial region not investigated |
| Blink reflex R2 amplitudes in cervicogenic headache, chronic tension-type headache and migraine | Sand *et al.* | 2006 | Cephalgia | TCN not investigated |
| Structure-function relationships in the rat brainstem subnucleus interpolaris: VI. Cervical convergence in cells deafferented at birth and a potential primary afferent substrate | Jacquin *et al.* | 1989 | Comparative Neurology | TCN not investigated |
| The Clinical Features, Risk Factors, and Surgical Treatment of Cervicogenic Headache in Patients With Cervical Spine Disorders Requiring Surgery | Shimohata *et al.* | 2017 | Headache | TCN not investigated |
| Modular organization of the head retraction responses elicited by electrical painful stimulation of the facial skin in humans | Serrao *et al.* | 2015 | Clinical Neurophysiology | TCN not investigated |
| Nerve growth factor injection into semispinal neck muscle evokes sustained facilitation of the jaw-opening reflex in anesthetized mice - possible implications for tension-type headache | Makovska *et al.* | 2005 | Experimental Neurology | TCN not investigated |
| Trigeminal excitation of dorsal neck motoneurones in the cat | Alstermark *et al.* | 1992 | Experimental Brain Research | TCN not investigated |
| Differences in sensory processing between chronic cervical zygapophysial joint pain patients with and without cervicogenic headache | Chua *et al.* | 2011 | Cephalgia | TCN not investigated |
| Probable relationship between trigeminal neuralgia and cervical column disease. Report of cases | Ovalle *et al.* | 2008 | Revista de la Sociedad Espanola del Dolor | TCN not investigated |
| Role of capsaicin-sensitive primary afferent inputs from the masseter muscle in the C1 spinal neurons responding to tooth-pulp stimulation in rats | Takeda *et al.* | 2005 | Experimental Brain Research | TCN not investigated |
| Somatostatin enhances tooth-pulp-evoked cervical dorsal horn neuronal activity in the rat via inhibition of GABAergic interneurons | Takahashi *et al.* | 2014 | Brain Research Bulletin | TCN not investigated |
| Prostaglandin E-2 potentiates the excitability of small diameter trigeminal root ganglion neurons projecting onto the superficial layer of the cervical dorsal horn in rats | Kadoi *et al.* | 2007 | Experimental Brain Research | TCN not investigated |

TCN: trigeminocervical nucleus.
