# Supplementary material for: Integration of nociceptive activity from orofacial, cranial and cervical regions in the trigeminocervical nucleus: a scoping review with clinical implications
Source: J Oral Facial Pain Headache. 2025 Sep 12;39(3):1–12. doi: 10.22514/jofph.2025.042 (PMC12531578; doi:10.22514/jofph.2025.042)
Supplement: Supplementary file 3 [file Supplementary-material-3.docx]

Supplementary material

Supplementary material 3. Study details.

| **Study** | **Population details** | **Stimulus** | **Outcomes** | **Results** | **Conclusions** |
| --- | --- | --- | --- | --- | --- |
| **Adachi, 2010 [1]**  **Country:** Japan  **Aim:** To examine the effects of pulpal application of purinergic receptor agonist on the expression of pERK*^1^ in Vi/Vc, Vc and C1/C2 neurons and if their activation is associated with nocifensive behavior reflected as an increase in EMG activity of masticatory muscles. | **Population mammals:**Rats  **Mean Age:** NR  **Sex:** Male  **Total sample size:**75  **Number of groups:**9 | **Type of stimulus:** Chemical  **Location of stimulus:**  Molar tooth pulp  **Location of response:** TMJ muscles and Vc/C1 C1/2  **TCN:**Vc, Vi  **Cervical Spine:** Upper cervical spinal cord C1/2 | **Main outcome:**  Number of PERK-LI cells (define changes in gene expression)  **Secondary outcome:**  TMJ-muscle activation in EMG | **Direction:**Orofacial to cervical  **Main outcome**: The number of pERK-LI*^1^ cells was significantly larger in both sides of Vc and in ipsilateral paratrigeminal nucleus following pulpal application of ATP compared to saline-injected rats. (Means that a change in gene expression is likely—>positive immunohistochemistry answer).  **Secondary outcome:** The mechanical threshold evoking EMG activity was significantly lower in genioglossus muscle compared with anterior digastric and masseter muscles. | **Conclusions**: The present findings suggest that specific ATP-binding receptors might contribute to the activation of tooth pulpal nerve fibers following tooth pulp injury, resulting in central sensitization of Vc, Vi/Vc, C1/C2, and the paratrigeminal nucleus neurons. |
| **Bereiter, 2002 [2]**  **Country:** USA  **Aim:**To test the hypothesis that vagal afferents contribute to opioid analgesia in TMD pain by quantifying c-fos*^2^ and to determine if vagotomy (VgX) influenced Fos-LI produced by TMJ injury or its reduction by morphine in a sex-dependent manner. | **Population mammals:**Rats  **Mean Age:** NR  **Sex:** Male  **Total sample size:**NR  **Number of groups:**4 (TMJ injury, TMJ injury + VgX, TMJ injury + morphine, TMJ injury +morphine + VgX; Control) | **Type of stimulus:** Chemical  **Location of stimulus:** Posterior mandibular condyle region  **Location of response:**  Caudal brainstem and upper cervical spinal cord  **TCN:**Vi/Vc  **Cervical Spine:** Vc/C2 | **Main outcome:**  Quantification of Fos-LI*^2^ in the lower brainstem and upper cervical spinal cord | **Direction:** Orofacial to cervical  **Main outcome**: Acute injury to the TMJ region caused a similar pattern of Fos-LI in the caudal brainstem and upper cervical spinal cord in males and females; Fos-LI was produced in three main regions of the trigeminal brainstem complex: paratrigeminal nucleus, Vi/Vc-vl (bilaterally), and at the junction of Vc with the upper cervical spinal cord (Vc/ C2; unilaterally). | **Conclusions**: Vagal afferents contribute to morphine-induced inhibition of Fos-LI produced in the Vi/Vc-vl transition region following TMJ injury in a sex-dependent manner. It is suggested that sex steroids play a role in mediating the interaction between vagal afferent activity and opioid analgesia in deep craniofacial pain conditions such as TMD. |
| **Bereiter, 2001 [3]**  **Country:** USA  **Aim:**To compare the pattern and magnitude of Fos-LI produced by TMJ injury in males to that of females at different stages of the estrous cycle; to determine if opioid receptor agonists, morphine and a selective kappa-opioid receptor agonist had similar effects on Fos-LI produced in males and females after TMJ injury. | **Population mammals:**Rats  **Mean Age:** NR  **Sex:** NR  **Total sample size:**NR  **Number of groups:**8 | **Type of stimulus:**  Chemical  **Location of stimulus:**  TMJ  **Location of response:**  Paratrigeminal nucleus, subnucleus interpolaris, caudalis transition region (Vi/Vc-vl), and in the superficial laminae at the subnucleus caudalis/upper cervical spinal cord (Vc/C2)  **TCN:**Brainstem  **Cervical Spine:** C1-C2 spinal cord | **Main outcome:** The mean number of Fos-positive neurons in several brainstem regions of interest | **Direction:**Orofacial to cervical  **Main outcome**: The number of Fos-positive neurons produced at the Vc/C2 junction was proportional to the concentration of mustard oil injected into the TMJ region; proestrus females produced higher levels of Fos-LI at the Vc/C2 junction than diestrus females or males; Morphine caused a greater dose-related reduction in Fos-LI at the dPa5 and Vc/C2 junction in males than females. | **Conclusions**:  Vc/C2 junction region plays a critical role in the integration of pain signals originating from the TMJ and may underlie sex differences in sensory processing related to TMJ pain. |
| **Busch, 2006 [4]**  **Country:** Germany  **Aim:**To determine whether a specific reduction of sensory cervical input in humans would influence the nociceptive transmission from the first division of the trigeminal nerve. | **Population mammals:**Humans  **Mean Age:** 19–31 years, mean age 24  **Sex:** Mixed  **Total sample size:**15  **Number of groups:** 1 | **Type of stimulus:** Electrical  **Location of stimulus:**  Forehead, supraorbital  **Location of response:**  Trigeminal brainstem  **TCN:** Caudal part  **Cervical Spine:** C1-C3 | **Main outcome:**  blink reflex R2 latencies response areas (AUC) | **Direction:**Orofacial to cervical  **Main outcome**: Significantly decreased R2 response areas and increased latencies of the R2 components on the injection side after the nerve blockade; After eliciting the blink reflex on the injection side, a significant increase of R2 latencies for ipsilateral and contralateral reflex responses; on the non-injection side a significant decrease of R2-response areas for ipsilateral and contralateral reflex responses. | **Conclusions**: Our findings extend previous results related to anatomical and functional convergence of trigeminal and cervical afferent pathways in animals and suggest that the modulation of this pathway is of potential benefit in primary headache disorders. |
| **Casatti, 1999 [5]**  **Country:** Brazil  **Aim:**To determine the origins of TMJ innervation. | **Population mammals:** Rats  **Mean Age:** NR  **Sex:** NR  **Total sample size:**10  **Number of groups:**1 (Tracer deposition) | **Type of stimulus:**  Mechanical  **Location of stimulus:**  TMJ  **Location of response:**  Trigeminal ganglion; cervical spine; Dorsal root Ganglia C2 to C5; Trigeminal mesencephalic nucleus  **TCN:** Trigeminal mesencephalic nucleus  **Cervical Spine:** C2 to C6 | **Main outcome:**  The absolute and percentage values of the labeled perikaryal profiles | **Direction:**Orofacial to cervical  **Main outcome**: Labeled perikaryal profiles were not observed in the contralateral superior cervical ganglion. In the ventral third, additional labeled perikaryal profiles were also located in the anterior region, in such a way that some of them were clearly located between the intersection of the maxillar and mandibular divisions of the trigeminal ganglion. | **Conclusions**: In conclusion, the rat TMJ is a target organ for sensory innervation derived mainly from the trigeminal ganglion and, to a lesser extent, from the dorsal root ganglia (C2 to C5). |
| **Chen 1986**  **Country:** Japan  **Aim:** Transganglionic transport of horseradish peroxidase (HRP) was used to study the termination patterns of somatic afferent fibers innervating oral and facial structures within the main nucleus (Vp), oral nucleus (Vo), and interpolar nucleus (Vi). | **Population mammals:** Cats  **Mean Age:** NR  **Sex:** NR  **Total sample size:**54  **Number of groups:**1 | **Type of stimulus:** Chemical  **Location of stimulus:** Lingual, buccal, inferior alveolar, superior alveolar, pterygopalatine, auriculotemporal, mylohyoid, anterior branch of mental, posterior branch of mental, zygomatic, infraorbital, frontal nerves  **Location of response:** Upper cervical cord  **TCN:** Main nucleus (Vp), oral nucleus (Vo), and interpolar nucleus (Vi)  **Cervical Spine:** Upper cervical cord | **Main outcome:**  Transganglionic transport of horseradish peroxidase (HRP) | **Direction:**Cervical to orofacial  **Main outcome**: HRP-labeled terminals of the primary afferents that in- nervate the intraoral structures were seen continuously through the dorsomedial regions of the ipsilateral trige- minal sensory nuclear complex (TSNC), i.e., the principal nucleus, pars oralis and pars interpolaris. Thus, the labeled terminal fields formed a rostrocaudally running column, although the density of afferent projections slightly varied from one rostrocaudal level of the TSNC to the next. | **Conclusions**: The relationship between the functional segregation and the cytoarchitectonic differentiation of the TSNC is discussed, particularly with respect to this somatotopic organization, combined with the characteristics of projecting cells in the TSNC. |
| **Chiaia, 1987 [6]**  **Country:** USA  **Aim:**To compare relations between the structural and functional properties of trigeminal primary afferents in the hamster and rat, at the level of individual fibers. | **Population mammals:**Hamster  **Mean Age:** Adult (>3 months)  **Sex:** NR  **Total sample size:**64  **Number of groups:** 1 | **Type of stimulus:**  Mechanical  **Location of stimulus:** Orofacial region  **Location of response:**  Trigeminal spinal tract, medullary dorsal horn  **TCN:** subnucleus interpolaris, medullary dorsal horn  **Cervical Spine:** C1 | **Main outcome:**  Number of boutons given off by each arbor | **Direction:**Orofacial to cervical  **Main outcome**:  Guard hair afferents generally had smaller arbors and gave rise to fewer boutons than vibrissa-sensitive axons; Like vibrissa afferents, their arbor was generally circumscribed in both interpolaris and MDH, but they were larger in the latter nucleus; Skin sensitive afferents had arbors that tended to be some- what larger than those of vibrissa-or guard-hair-related fibers. The arbors of skin-sensitive afferents were on average larger in interpolaris than MDH. | **Conclusions**: Vibrissa-sensitive primary afferents share similar morphology, with larger, dense arbors in the medullary dorsal horn (MDH). Guard hair afferents have smaller arbors. Skin-sensitive afferents exhibit larger arbors in interpolaris but tend to be larger overall. The organization of trigeminal input supports an “onion-leaf” somatotopic model with head inversion, although exceptions exist. |
| **Chibuzo [7]**  **Country:** EUA  **Aim:** To determine the location and origin of afferent fibers that innervate the tongue muscles in dogs. | **Population mammals:** Dogs  **Mean Age:** 2–6 weeks old  **Sex:** NR  **Total sample size:**13  **Number of groups:** 2 | **Type of stimulus:** Chemical  **Location of stimulus:** Tongue, intermandibular; extrinsic muscls of inteh left side  **Location of response:** Neurons in the cranial nerve ganglia; the trigeminal, geniculate, glossopharyngeal, and proximal and distal vagal ganglia  **TCN:** The spinal trigeminal nucleus caudalis (Vc) and rostral spinal trigeminal nucleus (Vr) in the brainstem  **Cervical Spine:** First cervical | **Main outcome:**  The identification of the origin of the afferent fibers to the lingual muscles | **Direction:**Cervical to orofacial  **Main outcome**: Afferent fibers to the lingual muscles originate from the cervical spinal cord segments C1-C3 and travel through the hypoglossal nerve. | **Conclusions**: The results of anatomic and physiologic studies in the sheep and pig leave little doubt that the cell bodies of afferent fibers from the extrinsic eye muscles reside in the ophthalmic zone of the trigeminal ganglion. |
| **Chudler, 1991 [8]**  **Country:** USA  **Aim:**To investigate the electrical and mechanical response properties of neurons located in the dorsal and ventral horns of the C1 segment of the spinal cord. | **Population mammals:**Cats  **Mean Age:** Adult  **Sex:** Mixed  **Total sample size:**13  **Number of groups:**1 | **Type of stimulus:**  Electrical and mechanical  **Location of stimulus:**  **Electrical:** Gingiva, trigeminal and C1/C2 dermatomes, sagittal sinus; **Mechanical:** Head neck and face  **Location of response:**  C1 spinal cord  **TCN:** Dorsal Horn  **Cervical Spine:** C1 | **Main outcome:**  Numbers of C1 neurons that responded to electrical stimulation of orofacial and cranial areas; response to graded mechanical stimulation applied to the skin | **Direction:** Orofacial to cervical  **Main outcome**:  52.2% of the responsive neurons were classified as LTM, 35.9% as WDR and 11.9% as NS; WDR neurons exhibited more convergence and had larger receptive fields than either NS or LTM neurons. WDR and NS neurons had longer first spike latencies than LTM neurons at all tested sites. Only WDR neurons were found to project to the contralateral caudal thalamus. | **Conclusions**: This study has confirmed earlier results demonstrating convergence of cervical and trigeminal information in the upper cervical spinal cord. The present study has demonstrated that the degree of convergence in the C1 spinal cord is related to the classification (*i.e.*, LTM, WDR or NS) of each neuron. |
| **Classey, 2001 [9]**  **Country:** UK  **Aim:**To determine the population effects on TNC neurons of amino acid blockade using a dose that is clearly effective in electrophysiological studies. | **Population mammals:**Cats  **Mean Age:** Adult  **Sex:** Mixed  **Total sample size:**11  **Number of groups:**3  (1) stimulated (2) stimulated + MK-801 (3) no stimulation | **Type of stimulus:** Electrical  **Location of stimulus:**  Sinus sagittalis superior  **Location of response:**  TNC; caudal medulla and C1/2/3  **TCN:** Caudal medulla  **Cervical Spine:** Upper cervical spinal cord | **Main outcome:**  The number of Fos-positive cells | **Direction:** Orofacial to cervical  **Main outcome**:  Number of Fos-IR cells in the TNC, following SSS stimulation preceded by amino acid antagonist injection, was 40; Taking the upper cervical cord together with the caudal medulla as a functional continuum, SSS stimulation produced Fos expression with 78 active nuclei in laminae I/IIo. Animals that received surgery, a 24-h rest and were then stimulated for 2 h exhibited levels of Fos-positive nuclei 2/8 times that of the unstimulated control. | **Conclusions**: The reduction in SSS stimulation-evoked Fos protein expression following administration of amino acid antagonist, indicates a role for glutamate in neurotransmission within the TNC complex. Glutamatergic mechanisms in the TNC complex offers insight and therapeutic possibilities for primary neurovascular headaches. |
| **Dermartini, 2017 [10]**  **Country:** Italy  **Aim:**To evaluate the effect of ion-channel-blocker in trigeminal hyperalgesia in an animal model of migraine. | **Population mammals:**Rats  **Mean Age:** NR  **Sex:** Male  **Total sample size:**52  **Number of groups:**4 | **Type of stimulus:**  Chemical  **Location of stimulus:**  Right lip  **Location of response:**  Medullary segment containing the TNC; Trigeminal ganglion and cervical spinal cord  **TCN:** Trigeminal nucleus caudalis  **Cervical Spine:** NR | **Main outcome:**  Expression levels of the genes/proteins encoding for c-Fos CGRP and Substance P (SP). | **Direction:** Orofacial to cervical  **Main outcome**: In rats bearing nitroglycerin-induced hyperalgesia, ion-channel-blocker showed an anti-hyperalgesic effect in the second phase of the orofacial formalin test. No differences between groups were seen as regards CGRP and SP protein expression in the TNC. | **Conclusions**: Ion channels play a key role in the behavioral responses associated with pain induced by nitroglycerin at trigeminal level and suggest that SP and CGRP release may contribute to the central and peripheral sensitization phenomenon. Ion-channel-blocking treatment could be promising to counteract hyperalgesia and pain. |
| **Du, 2017 [11]**  **Country:** China  **Aim:**To explore the effects of abnormal occlusion and functional recovery caused by functional mandible deviation on the head and neck muscles, muscle spindle sensorymotor system electrophysiological response and neurotransmitter distribution in the central nucleus. | **Population mammals:**Rats  **Mean Age:** seven-week-old  **Sex:** Male  **Total sample size:**67  **Number of groups:**2 (Functional mandibule deviation and control) | **Type of stimulus:**  Mechanical  **Location of stimulus:**  Jaw  **Location of response:**  Neck muscles and TNC  **TCN:** NR  **Cervical Spine**  Neck muscles: splenius and trapezius | **Main outcome:**  Histamine Release | **Direction:**Orofacial to cervical  **Main outcome**: After functional mandibular deviation, Histamin levels on the ipsilateral sides of the trigeminal nucleus increased significantly and histamine receptor protein levels on the ipsilateral sides of the trigeminal nucleus increased significantly. | **Conclusions**: After functional mandibular deviation, the TMJ mechanical receptors not only caused the fusimotor fiber hypoallergenic fatigue slow response on the ipsilateral sides of splenius, but also increased the injury neurotransmitter histamine release. The TMJ receptors may be involved in the mechanical theory of the head and neck muscles nervous system regulation. |
| **Goadsby and Zagami, 1991 [12]**  **Country:** Australia  **Aim:**To determine whether craniovascular nociception is limited exclusively to known trigeminal relays or whether other structures are involved. | **Population mammals:**Cats  **Mean Age:** Adult  **Sex:** Mixed  **Total sample size:**22  **Number of groups:**3 (electrically stimulation of SSS, both trigeminal ganglia blocked then SSS was stimulated, control) | **Type of stimulus:**  Electrical  **Location of stimulus:**  Sinus sagittalis superior (SSS)  **Location of response:**  Brainstem and upper cervical cord  **TCN**: Nucleus caudalis  **Cervical Spine:** Cervical dorsal horn | **Main outcome:**  SSS stimulation | **Direction:** Cervical to orofacial  **Main outcome**: An increase metabolic activity and blood flow in the trigeminal nucleus caudal is, in the cervical dorsal hom and in a discrete area in the dorsolateral spinal cord at the second cervical segment. Responses in these 3 areas were blocked by ablation of the trigeminal ganglia. The dorsolateral area activated in the spinal cord corresponds to a group of cells in or near the lateral cervical nucleus. | **Conclusions**: These experiments have demonstrated that activation of a pain-sensitive structure, the superior sagittal sinus (SSS), can increase glucose utilization and blood flow in specific regions of the brainstem and spinal cord via a neural pathway that relays in the trigeminal ganglion. |
| **Hathaway, 1995 [13]**  **Country:** Canada  **Aim:**To analyse the distribution of Fos-LI in the rat brainstem following noxious chemical stimulation of the TMJ complex and adjacent soft tissues and persistency of c-fos expression. | **Population mammals:**Rats  **Mean Age:** NR  **Sex:** Male  **Total sample size:**17  **Number of groups:**4 | **Type of stimulus:**  Chemical  **Location of stimulus:** TMJ  **Location of response:**  Brainstem and C1-C3  **TCN:** Vo; Vi; Vi/Vc; Vp; Vc  **Cervical Spine:** C1-C3 | **Main outcome:**  Number of Fos-LI Nuclei | **Direction:**Orofacial to cervical  **Main outcome**: Compared to TMJ mustard oil injection, mineral oil injection produced less Fos-LI at all rostrocaudal levels, whereas subcutaneous mustard oil injection produced less Fos-LI in caudal subnucleus caudalis but similar amounts in the cervical dorsal horn; Neither of these injections yielded significant ipsilateral responses in subnucleus caudalis. | **Conclusions**: The results strongly suggest that central trigeminal neurons responsive to noxious chemical stimulation of the TMJ are restricted to caudal portions of Vsp but have a more extensive rostrocaudal distribution than those neurons responsive to noxious stimulation of overlying superficial tissues. |
| **Honda, 2008 [14]**  **Country:** Canada  **Aim:**To evaluate mechanisms that may underlie the sensitization of Vc and C1-C2 nociceptive neurons to heat, cold and mechanical stimuli following topical capsaicin treatment of the rat's facial skin by assessing, nocifensive behaviors as well as pERK in Vc and C1-C2 neurons. | **Population mammals: Rats**  **Mean Age:** NR  **Sex:** NR  **Total sample size:**NR  **Number of groups:**3 (control, capsaicin-treated and vehicle-treated rats) | **Type of stimulus:**  Heat, cold and mechanical stimulus  **Location of stimulus:**  Facial skin  **Location of response:**  Vc and C1-C2 neurons  **TCN:** Vc  **Cervical Spine:** C1-C2 nociceptive neurons | **Main outcome:**  pERK-LI cells | **Direction:** Orofacial to cervical  **Main outcome**: pERK-LI cells were observed in Vc and C1-C2 within 2 min after cessation of heat stimulus (50 °C) and peaked at 4 min, and subsequently declined in number; pERK-LI cells was also observed in the superficial laminae of Vc and C1-C2 at 4 minutes after cold stimulus of the lateral facial skin in rats with vehicle or capsaicin treatment and in the superficial laminae of the Vc and C1-C2 at 4 min after non-noxious (6 g) and noxious (60 g) mechanical stimuli of the facial skin. | **Conclusions**: Capsaicin treatment of the lateral facial skin causes an enhancement of ERK phosphorylation in Vc and C1-C2 neurons as well as induces nocifensive behavior to heat, cold and mechanical simulation of the capsaicin-treated skin. |
| **Honda, 2011 [15]**  **Country:** Japan  **Aim:**To clarify whether peripheral Glutamate receptors may be involved in the central sensitization of Vc and C1-C2 neurons activated by noxious heat or cold stimulation of these orofacial tissues. | **Population mammals:**Rats  **Mean Age:** NR  **Sex:** NR  **Total sample size:**408  **Number of groups:**2 (Glutamate or vehicle injection) | **Type of stimulus:**  Heat and cold  **Location of stimulus:**  Tongue or whisker pad skin  **Location of response:**  Vc and C1-C2 neurons  **TCN:** Vc  **Cervical Spine:**  Splenius capitis muscle | **Main outcome:**  ERK phosphorylation | **Direction:**Orofacial to cervical  **Main outcome**: ERK phosphorylation in Vc and C1-C2 neurons was detected within 2 min and peaked at 5 min following subcutaneous Glutamate injection; pERK-IR neurons expressed in Vc and C1-C2 after Glutamate injection was significantly smaller in Glutamate- injected rats compared to that of vehicle-injected rats; pERK-IR neurons also peaked at the obex level after cold stimulation (5 °C) of the tongue following submucosal Glu injection into the tongue. | **Conclusions**: Peripheral Glutamate receptor mechanisms may contribute to cold hyperalgesia in the tongue but not in the facial skin, and also contribute to heat hyperalgesia in the tongue and facial skin, and that the mitogen-activated protein kinase cascade in Vc and C1-C2 neurons may be involved in these Glutamate-evoked hyperalgesic effects. |
| **Hu, 1992 [16]**  **Country:** Canada  **Aim:**To determine the effects of deep inputs excited by the small-fibred irritant mustard oil on trigeminal nociceptive neurons. | **Population mammals:**Rats  **Mean Age:** NR  **Sex:** NR  **Total sample size:**NR  **Number of groups:**1 | **Type of stimulus:**  Chemical/mechanical/natural  **Location of stimulus:**  Masseter muscle  **Location of response:**  Brainstem  **TCN:** Subnucleus caudalis/Vo  **Cervical Spine:** Spinal tract nucleus | **Main outcome:**  Alterations from the mean level of activity | **Direction:**Orofacial to cervical  **Main outcome**: Injection of 5% mustard oil into the deep masseter muscle produced a facilitator effect in 12 of 27 nociceptive neurons tested in caudalis and in 5 of 12 nociceptive neurons in oralis.  This effect was reflected in an expansion of the cutaneous mechanoreceptive field, an increase in spontaneous activity or an increase in responsivity to electrical stimulation of cutaneous afferent inputs to the neurons. | **Conclusions**: Mustard oil injection into the deep masseter muscle increased excitability of trigeminal nociceptive neurons, indicating a link between deep craniofacial afferents and pain mechanisms. |
| **Hu, 2005 [17]**  **Country:** Canada  **Aim:**The aim of this study was to characterize the properties of somatosensory neurons in the first 2 cervical spinal dorsal horns (C1 and C2 DHs) and compare them with those previously described for the rostral subnucleus caudalis (rVc). | **Population mammals:**Rats  **Mean Age:** Adult  **Sex:** Male  **Total sample size:**59  **Number of groups:**3 (wide-dynamic-range (WDR), nociceptive-specific (NS), 72 low-thresholdmechanoreceptive (LTM)) | **Type of stimulus:**  Mechanical, Electrical  **Location of stimulus:**  Deep craniofacial tissues  **Location of response:**  Craniofacial and cervical tissues  **TCN:** Rostral subnucleus caudalis (rVc)  **Cervical Spine:** C1 and C2 | **Main outcome:**  Properties of somatosensory neurons | **Direction:** Orofacial to cervical  **Main outcome**:  LTM neurons were located in laminae III/IV and had a small mechanoreceptive field (RF) that included the posterior face and cervical tissues. Nociceptive neurons were located in laminae I/II or V/VI, and the RF of each C1 and C2 DH nociceptive neuron included a part of the upper cervical. C1 and C2 DH nociceptive neurons received mechanosensitive convergent afferent inputs from cervical and craniofacial deep tissues, and over 50% could be activated by hypoglossal and electrical stimulation. | **Conclusions**: These features suggest that the caudal Vc and C1 and C2 DHs may act as one integrative functional unit to process cutaneous, deep, and visceral nociceptive information from craniofacial and spinal afferent inputs. |
| **Imbe, 1999 [18]**  **Country:** USA  **Aim:** Preprodynorphin PPD and preproenkephalin PPE gene expression in a rat model of orofacial inflammation were examined to further characterize the neurochemical mechanisms underlying orofacial inflammation and hyperalgesia. | **Population mammals:**Rats  **Mean Age:** NR  **Sex:** Male  **Total sample size:**NR  **Number of groups:**2 Stimulated and untreated rats (Naive group) | **Type of stimulus:**  Chemical  **Location of stimulus:**  TMJ and perioral skin  **Location of response:**  TNC and spinal cord  **TCN:**Vi/Vc  **Cervical Spine: C1-C3** | **Main outcome:** Gene expression | **Direction:**Orofacial to cervical  **Main outcome**: Gene expression level ipsilateral to TMJ inflammation was increased when compared to skin inflammation. In TMJ-inflamed rats gene expression-positive neurons were found ipsilaterally in the the upper cervical dorsal horn, the dorsal portion of the subnucleus caudalis and caudal subnucleus interpolaris, and the paratrigeminal nucleus. | **Conclusions**:  TMJ inflammation resulted in a more intense and widespread increase in gene expression when compared to skin inflammation. These changes may contribute to persistent central hyperexcitability, and pain associated with temporomandibular disorders. |
| **Jacquin, 1986 [19]**  **Country:** USA  **Aim:**To examine  structure-function relationships in the  medullary dorsal horn (MDH) and rostral  cervical dorsal horn. | **Population mammals:**Rats  **Mean Age:** Adult  **Sex:** Mixed  **Total sample size:** 68  **Number of groups:**2 (Intracellular Recording; horseradish  peroxidase*^3^ (HRP) labeling) | **Type of stimulus:**  Electrical, mechanical  **Location of stimulus:**  Ipsilateral V ganglion, hairs intermediate in length to vibrissae and guard hairs, nociceptive, and mucosal afferents  **Location of response:**  Caudal medulla and C1  **TCN:** medullary dorsal horn (MDH)  **Cervical Spine:** C1 | **Main outcome:**  Intracellular recording (axon’s response properties), Labeling | **Direction:**Orofacial to cervical  **Main outcome**:  In the magnocellular laminae of the MDH, all mystacial vibrissa primary afferents gave rise to similarly shaped arbors, regardless of their functional classification. While morphological within and between individual axons, variance between functional classes was no greater than that within a class. Nonmystacial vibrissa afferent arbors, had their primary arbor focus in C1 and Cz dorsal horn. | **Conclusions**: Structure-function relationships do exist for nonvibrissa V primary afferents in the MDH and cervical dorsal horn. Rat vibrissa afferent arborizations do not adhere to these structure-function relationships. |
| **Jacquin, 1983 [20]**  **Country:** USA  **Aim:**To demonstrate the central projections of the inferior orbital (IO) nerve in normal adult rats and in adult animals subjected to  IO nerve section and vibrissae follicle cauterization on the day of birth. | **Population mammals:**Rats  **Mean Age:** Adult  **Sex:** NR  **Total sample size:**25  **Number of groups:**2 (1-Normal; 2- neonatally lesioned rats) | **Type of stimulus:** Mechanical  **Location of stimulus:**  IO nerve, vibrissae follicle  **Locatio n of response:**  Subnucleus interpolaris, caudalis, oralis, C1, C2  **TCN: S**ubnucleus interpolaris, caudalis, oralis  **Cervical Spine:** C1, C2 | **Main outcome:** Central projections of the IO nerve | **Direction:**Unclear  **Main outcome**: In the trigeminal brainstem nuclear complex barrellike aggregates of HRP-labeled terminals could be seen throughout subnucleus interpolaris, subnucleus caudalis and C1. In adult rats subjected to neonatal IO nerve section and vibrissae follicle cauterization, transganglionic HRP*^3^ transport from the “regenerate” IO nerve indicated an almost exclusive projection to the marginal layer of the medullary and rostral cervical dorsal horn. | **Conclusions**: Adult rats subjected to neonatal IO nerve section and vibrissa follicle cauterization, transganglionic HRP transport from the “regenerate” IO nerve indicated an almost exclusive projection to the marginal layer of the medullary and rostral cervical dorsal horn. |
| **Kamimura, 2018 [21]**  **Country:** Japan  **Aim:**To study whether a cannabinoid degrade enzyme inhibitor relieved orofacial neuropathic pain associated with partial transection of the inferior orbitalis nerve (ION). | **Population mammals:** Mice  **Mean Age:** 8–9 weeks  **Sex:** Male  **Total sample size:** 20  **Number of groups:**4 | **Type of stimulus:**  Mechanical  **Location of stimulus:**  Whisker-pad area  **Location of response:**  Vc or C1-C2 tissue  **TCN:** Brainstem  **Cervical Spine:** Vc; C1-C2 | **Main outcome:**  Nocifensive behaviour: head-withdrawal threshold (HWT)  **Secondary outcomes:**  Immunoreactivity: largest number of labeled cells | **Direction:** Orofacial to cervical  **Main outcome**: HWT significantly reduced post-op in treatment groups; HWT significantly decreased 2 h post op in 4and 16 mg/kg;  **Secondary outcome:** No difference between sham and 16mg/kg group 2h post-op Immunohistochemistry: treatment-group cells in Vc and C1-C2 were greater in neuropathic than in sham. Two hours after injection, immunoreactive cells in the Vc and C1-C2 decreased. | **Conclusions**: The findings suggest that the examined cannabinoid degrade enzyme inhibitor is a promising therapeutic option for orofacial neuropathic pain. |
| **Kato, 2003 [22]**  **Country:** USA  **Aim:**To identify and functionally characterize the neuronal circuit of a new variant of the trigemino-cervical reflex involving the ventral cervical muscles in the swine. | **Population mammals:**Swine  **Mean Age:** 4–7 weeks  **Sex:** NR  **Total sample size:**32  **Number of groups:**3 (lateral approach, dorsal approach, ventral approach) | **Type of stimulus:**  Electrical  **Location of stimulus:**  Nostril  **Location of response:**  Brainstem/Cervical muscles  **TCN**: Gasserian ganglion and brainstem  **Cervical Spine:** C1/2 | **Main outcome:**  Trigeminal evoked potentials in different regions of the brain and head  **Secondary outcomes:** Magnetic field measurement | **Direction:**Orofacial to cervical  **Main outcome**: The reflex produced a large positive potential in the brain with a latency of about 14 ms. Its amplitude declined rapidly with stimulation rate above 0.5 Hz and was abolished by a muscle relaxant.  **Secondary outcome:** This generator was found to be the ventral cervical muscles that were activated by a circuit involving the brainstem trigeminal nuclei and cervical motor nuclei. | **Conclusions**: It is not known whether this new variant of the trigeminal cervical reflex is species-specific, or it also exists in humans. If it exists in humans, then it may be useful as a clinical tool for testing the integrity of the brainstem and C1 and C2 since the circuit is now identified and characterized in the animal model. |
| **Kaube, 1993 [23]**  **Country:** Australia  **Aim:**To map more precisely the locations of higher order neurons using the c-Fos*^2^ expresion in the medulla and the upper cervical spinal cord activated by SSS electrical stimulation. | **Population mammals:**Cats  **Mean Age:** NR  **Sex:** Female  **Total sample size:**6  **Number of groups:**2 (Control; sagital sinus stimulation) | **Type of stimulus:**  Electrical  **Location of stimulus:**  Sagittal sinus  **Location of response:**  Caudal Medula and upper cervical spinal cord  **TCN:** Caudal medula  **Cervical Spine:**  Upper cervical | **Main outcome:**  Fos expression in the caudal medulla and upper cervical dorsal horn | **Direction:**Orofacial to cervical  **Main outcome**:  Increases in Fos-like immunoreactivity in laminae 1 and Iio of the trigeminal nucleus caudalis and the dorsal horn of the upper cervical spinal cord. Fos-like immunoreactivity was observed in lamina X of the upper cervical spinal cord, in the commissural and medial nuclei of the solitary tract and in the nucleus retroambigualis. | **Conclusions**: The use  of immunohistochemical detection of Fos has allowed visualization of several populations of neurons likely to be involved in the central neural processing of vascular headache syndromes, particularly migraine. |
| **Kiyomoto, 2015 [24]**  **Country:** Japan  **Aim:**We analyzed the possible role of proteinkinase phosphorylation in activated microglia in ectopic orofacial pain. | **Population mammals:**Rats  **Mean Age:** NR  **Sex:** Male  **Total sample size:**230  **Number of groups:**3 | **Type of stimulus:**  Chemical/mechanical  **Location of stimulus:**  (1) trapezius muscle; (2) facial skin  **Location of response:**  VC  **TCN:** NR  **Cervical Spine:** NR | **Main outcome:**  Immunoreactivity in Vc,Inhibition of proteinkinase phosphorylation in Vc | **Direction:**Cervical to orofacial  **Main outcome**: HWT to mechanical stimulation (facial skin) decreased on day 4 after inhibitor injection (trapezius muscle) compared with saline-injected rats; Microglia in Vc showed immunoreactivity which had a large soma with thick processes on day 4 after inhibitor injection (trapezius muscle) compared with saline group; Normalized protein amount Vc ipsilateral to the inhibitor injection was significantly greater than that of saline injection after Day4; | **Conclusions**: Thus, proteinkinase phosphorylation inhibition in microglia may be a promising therapeutic target for treating ectopic pain associated with trapezius muscle pain. |
| **Kiyomoto, 2013 [25]**  **Country:** Japan  **Aim:**To determin the possible role of fractalkine (cytokine protein) and its receptor in the ectopic orofacial pain associated with trapezius muscle. | **Population mammals:**Rats  **Mean Age:** NR  **Sex:** Male  **Total sample size:**474  **Number of groups:**4 (trapezium or whisker pad; CFA or saline injection) | **Type of stimulus:**  Chemical/mechanical  **Location of stimulus:**  Wisker pad and trapezium  **Location of response:**  Vc, C1 to C6  **TCN:** Vc  **Cervical Spine:** C1 to C3 | **Main outcome:**  Fractalkine (FKN) | **Direction:** Cervical to Orofacial  **Main outcome**: Fractalkine expression was enhanced in Vc and C1–C2 or C2–C6 following trapezius muscle or facial skin inflammation, microglia are activated via Fractalkine signaling. | **Conclusions**: Fractalkine expression was enhanced in Vc and C1–C2 or C2–C6 following trapezius muscle or facial skin inflammation, microglia are activated via Fractalkine signaling, resulting in ectopic mechanical allodynia. |
| **Kobayashi, 2011 [26]**  **Country:** Japan  **Aim:**The aim of this study is to clarify the neural mechanisms underlying orofacial pain abnormalities after cervical spinal nerve injury. | **Population mammals:**Rats  **Mean Age:** NR  **Sex:** Male  **Total sample size:**NR  **Number of groups:**2 rats with upper cervical spinal nerve transection (CNX), (2 sham) | **Type of stimulus:**  Mechanical, thermal  **Location of stimulus:**  Lateral facial skin  **Location of response:**  Upper cervical spinal nerve transection (CNX)  **TCN:** Trigeminal spinal subnucleus caudalis (Vc)  **Cervical Spine:** C1-C2 | **Main outcome:**  mechanical allodynia and thermal hyperalgesia occur in the lateral facial skin after CNX and also suggest that ERK phosphorylation of Vc and C1-C2 neurons and  astroglial cell activation are involved in orofacial extraterritorial pain | **Direction:** Orofacial to cervical  **Main outcome**: pERK-LI*^1^ cells in superficial laminae of Vc and C1-C2 were larger in CNX compared to Sham following noxious and non-noxious mechanical or thermal stimulation of the lateral facial skin at day 7 after CNX. Heat and mechanical nocifensive behaviors were depressed and the number of pERK-LI*^1^ cells in Vc and C1-C2 following noxious and non-noxious mechanical stimulation of the face was decreased following i.t. administration of the astroglial inhibitor fluoroacetate. | **Conclusions**: The present findings have demonstrated that mechanical allodynia and thermal hyperalgesia occur in the lateral facial skin after CNX and also suggest that ERK phosphorylation of Vc and C1-C2 neurons and  astroglial cell activation are involved in orofacial extraterritorial pain following cervical nerve injury. |
| **Kramer, 2013 [27]**  **Country:** USA  **Aim:**Whether Gabrα6 (receptor can function to inhibit neuronal activity) has an inhibitory role in myofascial nociceptive responses similar to inflammatory TMJ arthritis. | **Population mammals:**Rats  **Mean Age:** NR  **Sex:** Male  **Total sample size:**NR  **Number of groups:**5 (control siRNA or Gabrα6 siRNA infusion, sham or ligature surgery and the day of treatment) | **Type of stimulus:**  Mechanical, chemical  **Location of stimulus:**  Mechanical: Anterior superficial portion of the masseter: Chemical: trigeminal ganglia bilaterally  **Location of response:**  V1, V3, Vc, C1  **TCN:** Vc  **Cervical Spine:** C1 | **Main outcome:**  Quantitation of Gaba receptor subunits in trigeminal ganglia and Vc-C1; neuronal activity (p-ERK*^1^) | **Direction:**Orofacial to Cervical  **Main outcome**:  Gabrα6 siRNA infusion reduced Gabrα6 and GABAA receptor expression and significantly increased the nociceptive response in both nociceptive assays. Gabra6 siRNA infusion also significantly increased trigeminal ganglia p-ERK*^1^ expression of the ligated rats. | **Conclusions**: We conclude GABAA receptors consisting of the Gabrα6 subunit inhibit trigeminal ganglia nociceptive sensory  afferents in the trigeminal pathway and have an important role in the regulation of myofascial.  nociception. |
| **Kramer, 2014 [28]**  **Country:** USA  **Aim:**Hypothesized that reducing Gabrα6 (receptor can function to inhibit neuronal activity) expression in the  trigeminal ganglia will increase the orofacial nociceptive response of ovariectomized female rats treated with  estradiol (synthetic estrogen). | **Population mammals:**Rats  **Mean Age:** NR  **Sex:** Female  **Total sample size:**NR  **Number of groups:**6 (Hormonal status (diestrus and proestrus) Surgery: (Ligature and sham operated rats), drug (Sham siRNA, Gabra SiRNA)) | **Type of stimulus:** Mechanical, chemical  **Location of stimulus:**  Mechanical: anterior superficial portion of the masseter; Chemical: trigeminal ganglia bilaterally  **Location of response:**  V1, V3, Vc, C1  **TCN:** Dorsal Vc  **Cervical Spine:** C1 | **Main outcome:**  Quantitation of Gaba receptor subunits | **Direction:**Orofacial to Cervical  **Main outcome**:  The main effect for infusion of Gabrα6 siRNA into the TG was a reduction in Gabrβ1 expression in both the trigeminal ganglia and Vc-C1 suggesting that total GABAA receptor content was reduced. After a high estradiol concentration Gabrβ1 expression increased in the trigeminal ganglia but not the Vc-C1. | **Conclusions**: Gabrα6 expression increased when rats received a proestrus dose of estradiol. Rats given a proestrus dose of estradiol also showed a decreased nociceptive response. Gabrα6 siRNA infusion, which has been shown to reduce Gabrα6 expression, increased the nociceptive response and reversed the attenuating effect of estradiol. |
| **Kurose, 2017 [29]**  **Country:** Japan  **Aim:**To determine if the preceding per- sistent unilateral monoarthritis of TMJ could facilitate MM nociception bilaterally indicated by pERK*^1^ and Fos expression*^2^ in the Vc/C2 region and formalin-evoked orofacial nocifensive behavioral activity. | **Population mammals:**Rats  **Mean Age:** NR  **Sex:** Male  **Total sample size:**110  **Number of groups:**3 CFA group formalin ipsilateral; CFA formalin contralateral; non-CFA | **Type of stimulus:** Chemical  **Location of stimulus:**  Midregion to the masseter muscle (MM)  **Location of response:**  Vc/C2 region  **TCN:**Medulla, brainstem  **Cervical Spine:** Vc/C2 | **Main outcome:**  average number of fos-positive cells | **Direction:**Orofacial to cervical  **Main outcome**: MM- evoked orofacial nocifensive behavioral responses are enhanced bilaterally after unilateral TMJ inflammation; Latency until pERK-IR cell reaches at the maximum level was delayed after nociceptive MM stimulation compared to skin stimulation under non-TMJ inflammatory; pERK and Fos positive cells evoked by MM stimulation increased at the Vc/C2 region ipsilateral to TMJ inflammation; pERK and Fos positive cells increased after MM stimulation contralateral to the affected side compared to non-CFA group. | **Conclusions**: Persistent unilateral inflammation of TMJ region increases orofacial nocifensive behavioral activities, pERK and Fos expression at the Vc/C2 region by MM injection of formalin independent of the side of injection. |
| **Lam, 2008 [30]**  **Country:** Canada  **Aim:** If a surgical incision of the facial skin and fascia overlying the TMJ can induce central sensitization in nociceptive neurons receiving TMJ afferent input in the Vc/upper cervical cord region. | **Population mammals:**Rats  **Mean Age:** NR  **Sex:** Male  **Total sample size:**65  **Number of groups:**2 (1: presence, 2: absence of lidocaine pretreatment) | **Type of stimulus:**  Mechanical, chemical  **Location of stimulus:**  TMJ  **Location of response:**  Trigeminal subnucleus caudalis/upper cervical cord  **TCN:** Trigeminal subnucleus caudalis  **Cervical Spine:** Upper cervical cord | **Main outcome:**  Neuronal activity | **Direction:**Orofacial to cervical  **Main outcome**:  Capsaicin injection into the TMJ induced cutaneous receptive field expansion, cutaneous mechanical activation threshold reduction and TMJ mechanical activation threshold reduction following lidocaine pretreatment of the cutaneous incision site whereas capsaicin injection following incision alone not only failed to induce further central sensitization but also decreased the existing incision-induced central sensitization in most neurons tested. | **Conclusions**: Central sensitization induced by capsaicin alone or by cutaneous incision alone can readily occur in TMJ-responsive nociceptive neurons and that following incision-induced excitability increases, capsaicin may result in a temporary  suppression of nociceptive neuronal changes reflecting central sensitization. |
| **Lam, 2009 [31]**  **Country:** Canada  **Aim:**Examined the effect of the peripheral application of glutamate and capsaicin to the TMJ in influencing the activation and central sensitization of  TMJ-responsive nociceptive neurons in the Vc/upper cervical cord. | **Population mammals:**Rats  **Mean Age:** NR  **Sex:** Male  **Total sample size:**49  **Number of groups:**2 (1: glutamate control + capsaicin; 2: glutamate + capsaicin) | **Type of stimulus:**  Mechanical, chemical  **Location of stimulus:**  TMJ  **Location of response:**  Trigeminal subnucleus caudalis/upper cervical  cord (Vc/UCC)  **TCN:** Trigeminal subnucleus caudalis  **Cervical Spine:** upper cervical cord | **Main outcome:**  Cutaneous receptive field size  and mechanical activation threshold of the TMJ-responsive nociceptive neurons | **Direction:**Orofacial to cervical  **Main outcome**: When injected alone, glutamate and capsaicin activated and induced central sensitization in most Vc/upper cervical cord neurons. Following glutamate injection, capsaicin evoked greater activity and less cutaneous/TMJ mechanical activation threshold reduction compared with capsaicin alone, whereas capsaicin abolished all subsequent glutamate-evoked activity and depressed cutaneous receptive field expansion in most neurons. | **Conclusions:** Central sensitization can be readily induced by both cutaneous incision and capsaicin injection, and capsaicin may even lead to a temporary suppression of nociceptive neuronal changes following incision-induced excitability increases. |
| **Li, 2014 [32]**  **Country:** USA  **Aim:**To study whether chronic constriction injury to the infraorbital nerve (CCI-ION) caused calcium channel  protein dysregulation in trigeminal ganglia and  associated spinal subnucleus caudalis and C1/C2 cervical dorsal spinal cord (Vc/C2). | **Population mammals:**Rats  **Mean Age:** NR  **Sex:** Male  **Total sample size:**NR  **Number of groups:**2 (Control; Experimental) | **Type of stimulus:** Mechanical  **Location of stimulus:**  TMJ  **Location of response:** trigeminal subnucleus caudalis/upper cervical cord  **TCN:** Dorsal Vc  **Cervical Spine:** C1, C2 | **Main outcome:**  Immunochemistry | **Direction:** Orofacial to cervical  **Main outcome**: Three weeks after CCI-ION, the intensity of calcium channel immunoreactivity was increased in the superficial, dorsal horn of Vc/C2 in the injury side compared with that in the non-injury side. | **Conclusions**: CCI-ON  leads to calcium channel up-regulation in dorsal Vc/C2 spinal cord  that may contribute to the development of orofacial neuropathic pain states through a central mechanism involving abnormal sensitization of Vc/C2 neurons by enhanced excitatory synaptogenesis and presynaptic release of excitatory neurotransmitters. |
| **Li, 2013 [33]**  **Country:** USA  **Aim:**To examine glucoproteine expression in trigeminal ganglia (TG) and associated trigeminal spinal complex of  dorsal spinal subnucleus caudalis and C1/C2 spinal cord (Vc/C2) in an orofacial neuropathic  pain model derived from trigeminal nerve injury and whether altered glucoproteine expression contributed to the development of orofacial neuropathic pain states. | **Population mammals:**Rats  **Mean Age:** NR  **Sex:**  Male  **Total sample size:**73  **Number of groups:**2 (1-CCI-Ion 2-sham) | **Type of stimulus:**  Mechanical, Chemical  **Location of stimulus:**  1 - Mechanical: infraorbital nerve (ION); 2 - Chemical: Vc/C2  **Location of response:**  1- Vc, C1, C2; 2- vibrissal pad  **TCN:** Vc  **Cervical Spine:** C1, C2 | **Main outcome:**  Glucoproteine expression alteration in Vc/C2 | **Direction:**Cervical to orofacial/Orofacial to cervical  **Main outcome**: Trigeminal nerve injury induced glucoproteine upregulation in Vc/C2 at  a time point correlated with orofacial tactile allodynia. Intrathecal treatment with glucoproteine antisense, but not mismatch, oligodeoxynucleotides blocked both injury-induced glucoproteine upregulation in Vc/C2 and behavioral hypersensitivity. | **Conclusions**: Infraorbital nerve injury leads to glucoproteine upregulation in  trigeminal spinal complex that contributes to orofacial neuropathic pain states. |
| **Liu, 2012 [34]**  **Country:** Japan  **Aim:**To establish a novel experimental model of inflammatory tongue pain by submucosal injection of  CFA into the tongue. | **Population mammals:**Rats  **Mean Age:** Adult  **Sex:** Male  **Total sample size:**NR  **Number of groups: 2** (CFA-OR saline injected  Tongue) | **Type of stimulus:**  Mechanical, Thermal  **Location of stimulus:**  Splenius capitis muscle (tongue)  **Location of response:**  Both ipsilateral and contralateral trigeminal  spinal subnucleus caudalis (Vc) and upper cervical spinal cord (C1-C2)  **TCN:** Vc  **Cervical Spine:** C1, C2 | **Main outcome:**  ERK phosphorylation in Vc and C1-C2 | **Direction:**Cervical to orofacial/Orofacial to cervical  **Main outcome**: Submucosal injection of CFA into the tongue produced a long-lasting mechanical allodynia and heat hyperalgesia at the inflamed site, concomitant with an increase in the pERK*^1^ immunoreactivity in the Vc and C1-C2. An enhanced activation of ERK in the Vc and C1-C2 following CFA injection. Administration of the pERK inhibitor and a selective gluatamate antagonist significantly depressed the mechanical allodynia and heat hyperalgesia in the CFA-injected tongue. pERK-IR cells in ipsilateral Vc and C1-C2 was also decreased by both drugs. | **Conclusions**: The present study constructed a new animal model of inflammatory tongue pain in rodents, and  demonstrated pivotal roles of glutamate-pERK signaling in the development of mechanical and heat  hypersensitivity that evolved in the inflamed tongue. |
| **Luo, 1995 [35]**  **Country:** USA  **Aim:**Intracellular staining with biotinamide was used to study the axonal projection and synaptic morphology of rat jaw-muscle spindle afferents. | **Population mammals:**Rats  **Mean Age:** NR  **Sex:** NR  **Total sample size:**41  **Number of groups:**5 | **Type of stimulus:**  Stretching of the jaw-closing muscles  **Location of stimulus:**  Diastema of the mandible, so that the jaw muscles could be stretched  **Location of response:**  Caudal Brainstem  **TCN:** Caudal Brainstem  **Cervical Spine:** NR | **Main outcome:**  Distribution of biotinamide-labeled  jaw-muscle spindle afferent axons | **Direction:**Orofacial to cervical  **Main outcome**: Collaterals and boutons from axons labeled with biotinamide were observed in the trigeminal motor nucleus  (Vrno), the region dorsal to Vmo including the supratrigeminal region (Vsup), the dorsomedial portion of the trigeminal principal sensory nucleus (Vpdm), Vodm and adjacent reticular formation, Vidm and adjacent reticular formation, Vc, the dorsal division of the medullary reticular field (MdD), and the caudal part of Vme. | **Conclusions:** This projection of jaw-muscle spindle afferents to caudal brainstem regions may play a significant role in masticatory-muscle stretch reflexes and in the integration of trigeminal proprioceptive information and its transmission to higher centers. |
| **Luz, 2019 [36]**  **Country:** Portugal  **Aim:**To study the properties of TNC lamina I neurons and efficacy of their trigeminal inputs; to examine caudal projections of thin trigeminal afferents to the medullary and cervical superficial dorsal horns. | **Population mammals:**Rats  **Mean Age:** NR  **Sex:** NR  **Total sample size:** NR  **Number of groups:**1 | **Type of stimulus:** Electrical  **Location of stimulus:**  the trigeminal nerve together with its ophthalmic branch V1  **Location of response:**  medullary dorsal horn and the cervical segments C1 to C4  **TCN:** medullary dorsal horn or Vc  **Cervical Spine:** C1, C2, C3, C4 | **Main outcome:**  Segmental distribution of excitatory and inhibitory inputs | **Direction:**Orofacial to cervical  **Main outcome**: The monosynaptic inputs from the trigeminal Ad, high-threshold Ad, low-threshold C, and C afferents were recorded in the medullary neurons, as well as in the cervical neurons located in the segments C1 to C2 and, to a lesser degree, in C3 to C4. This pattern of supply was consistent with our labelling experiments showing extensive cervical projections of trigeminal afferents. | **Conclusions**: The trigeminocervical lamina I neurons receive  a complex pattern of long-range monosynaptic and polysynaptic inputs from a variety of the trigeminal nociceptive afferents. |
| **Lyubashina, 2012 [37]**  **Country:** Russia  **Aim:**To determine if a vagal nerve stimulation (VNS) effects the neuronal activity of the spinal trigeminal nucleus. | **Population mammals:**Rats  **Mean Age:** Adult  **Sex:** Male  **Total sample size:**20  **Number of groups:**2 (preconditioning VNS, continuous VNS) | **Type of stimulus:**  Electrical/mechanical  **Location of stimulus:**  Left vagal nerve and facial cutaneous receptive field  **Location of response:**  Left STN at the level of C1 spinal cord  **TCN:** Spinal trigeminus nucleus (STN)  **Cervical Spine:** C1 | **Main outcome:**  Mean number of spikes per second or a mean number of spikes per stimulus | **Direction:**Orofacial to cervical  **Main outcome**: Continuous stimulation of the left cervical vagus nerve produces predominantly inhibitory effect on responses of the convergent spinal trigeminal neurons to electrical stimulation of the dura mater;  with the decrease in evoked activity, the majority of tested trigeminal units demonstrated VNS-induced depression of ongoing firing | **Conclusions**: The predominantly inhibitory effect of the left cervical VNS on activity of the second-order sensory trigeminal neurons in the rat model of headache. The described vagal afferent modulation of the spinal trigeminal neurons may play a role in the ascending visceral regulation of processing of sensory information from the dura mater. |
| **Lyubashina, 2017 [38]**  **Country:** Russia  **Aim:**To investigate the effects of GON electrical stimulation with parameters comparable to the ones used in the clinic on activity of the dura-sensitive spinal trigeminal neurons in a rat model of trigemino-durovascular nociceptionthe activity of the TCC cells with convergent meningeal, orofacial and cervico-occipital inputs and the changes in their ongoing firing and responses to electrical stimulation of the dura mater. | **Population mammals:**Rats  **Mean Age:** NR  **Sex:** Male  **Total sample size:**11  **Number of groups:** 5 | **Type of stimulus:**  Electrical  **Location of stimulus:**  Dura mater close to the superior sagittal sinus; distal part of the GON  **Location of response:**  C1  **TCN:** Trigeminal nucleus caudalis  **Cervical Spine:** C1 | **Main outcome:**  Spike activity of TCC cells | **Direction:**Orofacial to cervical  **Main outcome**: At all parameter sets applied, preconditioning GON stimulation caused a decrease in ongoing activity of the spinal trigeminal neurons (2) The inhibitory effect depended mostly on the GON stimulation intensity (3) None of the GON stimulation parameter sets produced long-lasting changes in the evoked neuronal activity, which in 10 min after each successive GON stimulation, just prior the next one, did not differ from its initial level. | **Conclusions**: Preconditioning high-frequency suprathreshold electrical stimulation of the greater occipital nerve produces suppression of both the ongoing activity of the convergent spinal trigeminal neurons and their responses to electrical stimulation of the dura mater. The inhibitory effect depended on the stimulation parameters, being maximally pronounced when a stimulus of 6 V was applied to the GON. |
| **Marfurt and Turner, 1984 [39]**  **Country:** USA  **Aim:**To map the central projections of trigeminal primary afferent neurons that innervate the dental pulp organ of the rat first maxillary molar tooth. | **Population mammals:**Rats  **Mean Age:** Adult  **Sex:** Male  **Total sample size:**10  **Number of groups:**2 (40% HRP*^3^; 5% HRP-WGA) | **Type of stimulus:**  Chemical  **Location of stimulus:**  First maxillary pulp chamber  **Location of response:**  Brainstem, cerebellum, spinal cord segments C1 and C2, and both trigeminal ganglia  **TCN:** Brainstem, cerebellum  **Cervical Spine:** Spinal cord C1/2 | **Main outcome:**  Locations of HRP-labeled fibers and terminal fields | **Direction:**Orofacial to cervical  **Main outcome**: HRP-labeled central processes of the tooth pulp afferent neurons terminated in significant numbers in each of the four rostrocaudal subdivisions of the ipsilateral TNC. The labeled terminal fields formed a column that stretched relatively uninterrupted from just caudal to the rostromedial tip of the trigeminal principal sensory nucleus to the caudal pole of the second cervical segment of the spinal cord. No labeled fibers at any level of the TNC crossed the midline to end in the contralateral TNC or upper cervical dorsal horn. | **Conclusions**: Acute injury to the TMJ region by removing first maxillary tooth and injecting HRP leads to an activation in TNC. |
| **Marfurt *et al.* 1981** [40]  **Country:** EUA  **Aim:** To determine the extent to which trigeminal primary afferent fibers convey somatosensory information to areas of the central nervous system outside of the TBNC. | **Population mammals:** Rats  **Mean Age:** NR  **Sex:** Female  **Total sample size:**20  **Number of groups:**1 | **Type of stimulus:** Chemical  **Location of stimulus:** Three main divisions of the trigeminal ganglion  **Location of response:** Mandibular and ophthalmic nerve  **TCN:** Trigeminal brainstem nuclear complex (TBNC)  **Cervical Spine:** C1-C7 | **Main outcome:**  Projections of the neurons | **Direction:** Cervical to orofacial  **Main outcome:** Labeled terminals were observed in all dorsoventral and mediolateral areas of the trigeminal main sensory nucleus, pars oralis, and pars interpolaris, and in all laminae of pars caudalis and the contiguous dorsal horn of the C1 spinal cord; The spinal cord and completely filled the dorsal horn of the C1 segment. Labeling intensity was high in all laminae but was particularly dense in laminae I and 11. In C2 and C3, the amount of terminal labeling decreased and was concentrated mainly in a wedge-shaped, intermediate area of the dorsal horn, as well as in laminae I throughout its entire mediolateral extent. Only modest amounts of reaction product were observed in levels C4 and C5, where most of it was concentrated in laminae I, 11, and V. A few isolated fibers descended as far caudally as C6 and C7. | **Conclusions:** This study has demonstrated that somatosensory information from the head and face may be transmitted directly to widespread and functionally heterogeneous areas of the rat central nervous system, including the spinal cord dorsal horn, numerous brainstem nuclei, and the cerebellum. |
| **Margolis, 1989 [41]**  **Country:** USA  **Aim:**To assess the location of brainstem neurons that initially become infected with herpes simplex virus after inoculation of the anterior chamber of the eye and to study the sequential spread of the virus within the brainstem and to other areas of the CNS. | **Population mammals:** Mice  **Mean Age:** 8–12 weeks  **Sex:** NR  **Total sample size:**22  **Number of groups:** 2 | **Type of stimulus:**  Chemical  **Location of stimulus:**  Chamber of the left eye  **Location of response:** Brainstem  **TCN:** Brainstem  **Cervical Spine:** NR | **Main outcome:** Number of labeled neurons | **Direction:**Orofacial to cervical  **Main outcome**: Labeled neurons were first noted in the CNS at 4 days postinoculation in the Edinger-Westphal nucleus, ipsilateral spinal trigeminal nucleus, pars caudalis, pars interpolaris, and ipsilateral dorsal horn of the rostral cervical spinal cord. By 5 days post inoculation, additional sites of labeling included the seventh nerve nucleus, nucleus locus coeruleus, and the nuclei raphe magnus and raphe pallidus. By 7 days postinoculation, no new foci of labeled cels were noted in the brainstem. | **Conclusions**: Our findings suggest that the resultant CNS infection is not diffuse but rather is restricted to a small number of non-contiguous foci in the brain stem and forebrain. The infected areas largely appear to be synaptically related, which suggests that the principal route of spread of infection in the CNS is transneuronal, presumably via axonal transport. |
| **Matsumoto, 1999 [42]**  **Country:** Japan  **Aim:**To verify the effects of electrical stimulation of the ipsilateral tooth pulp on C1 spinal neurons. | **Population mammals:**Rats  **Mean Age:** Adult  **Sex:** Male  **Total sample size:**33  **Number of groups:**1 | **Type of stimulus:**  Electrical  **Location of stimulus:**  Tooth pulp and phrenic nerve fibers  **Location of response:**  C1 spinal neurons  **TCN:** Caudalis  **Cervical Spine:** C1 | **Main outcome:**  Neuronal activity | **Direction:**Orofacial to cervical  **Main outcome**: Excitatory receptive somatic fields were determined in  28 neurons, and somatic field locations of 17 neurons (60.7%) included the ipsilateral face, neck, and jaw. Both  noxious pinch and brushing hair excited all 28 neurons. | **Conclusions**: There may be the convergence of face, neck, jaw, tooth pulp, and phrenic nerve afferents on the same C1 spinal neurons in the rat. |
| **Miyamoto, 2011 [43]**  **Country:** Japan  **Aim:**To clarify the involvement of glutamate subunits of Cation channel receptors in orofacial neuropathic pain | **Population mammals:**Rats  **Mean Age:** Adult  **Sex:** Male  **Total sample size:** 33  **Number of groups:**1 | **Type of stimulus:**  Mechanical  **Location of stimulus:**  Whisker pad skin  **Location of response:**  Vc and C1–C2  **TCN:** Vc  **Cervical Spine:** C1–C2 | **Main outcome:**  Extracellular-signal regulated  kinase (ERK) phosphorylation*^1^ | **Direction:**Orofacial to cervical  **Main outcome**: The number of pERK-LI cells examined in Vc and C1–C2 in wild-type mice after the non-noxious stimulation was larger than that of glutamate-injected mice. | **Conclusions**: The present findings suggest that glutamate subunits of Cation channel receptors play roles in the  trigeminal nerve injury-mediated enhancement of Vc and C1–C2 neuronal excitability, and  hyperalgesia. |
| **Morch, 2007 [44]**  **Country:** Canada  **Aim:**To investigate the convergence of cutaneous, musculoskeletal, dural and visceral afferents onto nociceptive neurons in the first cervical dorsal horn  in urethane ⁄ chloralose-anesthetized rats. | **Population mammals:**Rats  **Mean Age:** Adult  **Sex:** Male  **Total sample size:**58  **Number of groups:**2 (1: wide dynamic range 2: nociceptive-specific) | **Type of stimulus:**  Mechanical, chemical, Electrical  **Location of stimulus:**  Facial, neck, shoulder and forepaw skin, cornea, dura, C2 nerve, hypoglossal nerve, TMJ, masseter muscle and superior laryngeal nerve.  **Location of response:**  First cervical dorsal horn  **TCN:** Trigeminal (V)  **Cervical Spine:** C2 | **Main outcome:**  activation of the nociceptive neurons in the first cervical dorsal horn | **Direction:** Orofacial to cervical  **Main outcome**:  The proportion of neurons responding to corneal, dural, C2 nerve, hypoglossal nerve, TMJ, MAS muscle and superior laryngeal nerve stimulations was 87, 54, 85, 52, 73, 64 and 31%, respectively. Electrical stimulation showed a double logarithmic stimulus–response relation, and cluster analysis of the excitability to corneal, musculoskeletal, dural and visceral stimulations revealed two groups of neurons, one mainly containing wide dynamic range neurons and one mainly containing nociceptive-specific neurons. | **Conclusions**: These findings indicate that afferent convergence in first cervical dorsal horn nociceptive neurons may be limited to the craniofacial area. |
| **Nakajima, 2011 [45]**  **Country:** Japan  **Aim:**To clarify the involvement of protein kinase Cγ (PKCγ) in the facial neuropathic pain following infraorbital nerve injury. To test the change of PKCγ expression in the  trigeminal spinal subnucleus caudalis (Vc) and upper  cervical spinal cord (C1/C2) following chronic constriction injury of the infraorbital nerve (ION-CCI). | **Population mammals:**Rats  **Mean Age:** Adult  **Sex:** Male  **Total sample size:**58  **Number of groups:**1 | **Type of stimulus:**  Mechanical  **Location of stimulus:**  Infraorbital nerve  **Location of response:**  Vc, C1/C2  **TCN:** Vc  **Cervical Spine:** C1/C2 | **Main outcome:** PKCγ Expression Followed ION-CCI | **Direction:**Orofacial to cervical  **Main outcome**: PKCγ-LI fibers and cells were distributed in the IIi of Vc. Relative density of the PKCγ immuno-product in the Vc was larger in ION-CCI rats at days 3 and 7 after ION-CCI compared with sham. Density of the PKCγ immuno-product in the C1/C2 was not different between ION-CCI and sham rats. No changes in the relative density of PKCγ immuno-product in the contralateral Vc. | **Conclusions**: These findings suggest that PKCγ in the Vc IIi is involved in induction and maintenance of the excitability of Vc neurons, resulting in static mechanical allodynia in the orofacial region following trigeminal nerve injury. |
| **Nishimori, 1986 [46]**  **Country:** Japan  **Aim:**To investigate the central projection of muscle afferents from the masseter nerve to the trigeminal sensory nuclear complex. | **Population mammals:**Cats  **Mean Age:** NR  **Sex:** NR  **Total sample size:**NR  **Number of groups:**2 | **Type of stimulus:**  Chemical  **Location of stimulus:** Masseter nerve  **Location of response:**  TSNC  **TCN:** Nucleus interpolaris and caudalis  **Cervical Spine:** Upper cervical dorsal horn | **Main outcome:**  Distribution of  HRP*^3^-labeled cells and axons in the trigeminal ganglion. | **Direction:**Orofacial to cervical  **Main outcome**: The central processes of the masseteric nerve terminated in the caudal third of the pars interpolaris, and laminae I/V through the caudal twothirds of caudalis and rostral parts of the C1 spinal cord segment. | **Conclusions**: Muscle pain from the masseter muscle is relayed to the second-order neurons in the caudal parts of the pars interpolaris and laminae I/V, and subsequently mediated to the sensory cortex via VPM and posterior nucleus (POre). |
| **Noma, 2008 [47]**  **Country:** Japan  **Aim:**To define the somatotopic arrangement of neurons in the trigeminal spinal subnucleus caudalis and upper cervical cord activated by acute noxious stimulation of various orofacial sites. | **Population mammals:**Rats  **Mean Age:** NR  **Sex:** Male  **Total sample size:**154  **Number of groups:**1 | **Type of stimulus:** Chemical  **Location of stimulus:**  Tongue, lower gum, upper and lower lips  **Location of response:** Trigeminal Spinal Nucleus Caudalis and Upper Cervical Cord  **TCN:** Trigeminal Spinal Nucleus Caudalis  **Cervical Spine:** Upper cervical spinal cord | **Main outcome:**  pERK-Immunoreactive*^1^ | **Direction:**Cervical to orofacial/Orofacial to cervical  **Main outcome**: The rostrocaudal distribution area of pERK-LI cells was more extensive from the Vi/Vc zone to the Vc/C2 zone after intraoral injection than that after facial injection, and the rostrocaudal distribution of pERK-LI cells from the Vi/Vc zone to the Vc/C2 zone had a somatotopic arrangement, with the snout being represented most rostrally and ophthalmic, ocular, or mental regions represented most caudally. | **Conclusions**: The capsaicin-sensitive small-fiber afferent inputs acutely activated by stimulation of facial and intraoral sites can produce ERK phosphorylation in the Vi/Vc zone, middle Vc, and Vc/C2 zone. |
| **Noma, 2017 [48]**  **Country:** Japan  **Aim: To** investigate the effects of Botulinum neurotoxin type A on mechanical allodynia and hyperalgesia in ION-CCI rats and examined if Botulinum neurotoxin type A attenuates excitability in Vc neurons by using pERK*^1^ immunohistochemistry. | **Population mammals:** Rats  **Mean Age:** Adult  **Sex:** Male  **Total sample size:** 20  **Number of groups:**4 | **Type of stimulus:**  Mechanical  **Location of stimulus:**  Infraorbital nerve  **Location of response:**  Vc and C1-C2  **TCN:** Trigeminal spinal subnucleus caudalis (Vc)  **Cervical Spine:** Upper cervical spinal cord (C1-C2) | **Main outcome:** Number and distribution pattern of the phosphorylated extracellular signal-regulated kinase (pERK)-immunoreactive (IR). | **Direction:**Orofacial to cervical  **Main outcome**: On day 21, nocifensive behavior was attenuated by high-dose but not low-dose Botulinum neurotoxin type A administration. After noxious mechanical stimulation of whisker pad skin, the numbers of pERK-IR cells in the superficial laminae of Vc and C1-C2 were significantly lower in the high-dose Botulinum neurotoxin type A group than in the ION-CCI + saline group. | **Conclusions**: Intradermal injection of high-dose Botulinum neurotoxin type A at the site innervated by ION alleviates ION-CCI-induced mechanical allodynia and hyperalgesia and Vc neuronal responses after noxious stimulation. |
| **Nomura, 2002 [49]**  **Country:** Japan  **Aim:**To clarify the central mechanism of allodynia-like responses in the adjacent region of the face following inferior alveolar nerve transection by employing the Fos-technique to explore changes in number and spatial arrangement of the Vc and the first segment of the spinal cord (C1) neurons. | **Population mammals:**Rats  **Mean Age:** NR  **Sex:** Male  **Total sample size:**80  **Number of groups:**2 (1: experimental 2: sham) | **Type of stimulus:**  Mechanical  **Location of stimulus:**  Whisker pad  **Location of response:**  Trigeminal spinal nucleus caudalis and upper cervical cord  **TCN:** Trigeminal spinal nucleus caudalis (Vc)  **Cervical Spine:** First segment of the spinal cord (C1) | **Main outcome:**  Number of Fos protein-LI cells*^2^ | **Direction:**Orofacial to cervical  **Main outcome**:  Fos protein-LI cells were expressed bilaterally in the Vc and C1, but were more numerous on the ipsilateral side to transection than on the contralateral side. The largest number of Fos protein-LI cells was observed at 2400 mm caudal from the trigeminal subnucleus interporalis (Vi)–Vc border both in ipsilateral and contralateral sides. The number of Fos protein-LI cells increased after application of 1, 4 and 16 g stimuli as compared to rats without mechanical stimulation. | **Conclusions**: The change in the numbers and spatial arrangement of nociceptive neurons in the Vc and C1 after inferior alveolar nerve transection reflect the development of mechanical hyperalgesia in the area adjacent to the IAN innervated region. |
| **Ogawa, 2003 [50]**  **Country:** Japan  **Aim:**To study the mechanisms of trigeminal pain following chronic parotid gland inflammation, a model of chronic parotitis was introduced in rats with an injection of Complete Freund’s adjuvant (CFA) into one parotid gland. | **Population mammals:**Rats  **Mean Age:** NR  **Sex:** Male  **Total sample size:**10  **Number of groups:**2 (CFA parotid gland group; 2 saline parotid gland group) | **Type of stimulus:**  Mechanical  **Location of stimulus:**  Unilateral parotid gland / left lateral portion of the face  **Location of response:**  Vi, Vc, paratrigeminal nucleus, C2  **TCN:** Vi, Vc, paratrigeminal nucleus  **Cervical Spine:** C2 | **Main outcome:**  Trigeminal Fos protein expression*^2^ | **Direction:** Orofacial to cervical  **Main outcome**: A large number of Fos protein-LI cells were expressed bilaterally in the ventral portion of the Vi/Vc. Fos protein-LI cells in the C2 of the upper cervical cord were found on the ipsilateral side to the CFA injection. Bilateral expression of Fos protein-like immunoreactive cells was observed in the transition zone between the Vi, Vc and paratrigeminal nucleus. A significant unilateral expression of Fos protein-positive cells was observed on the ipsilateral side of the C2 dorsal horn. | **Conclusions**: A large number of Fos protein-LI cells were expressed bilaterally in the ventral portion of the Vi/Vc and in the C2 after the stimulus of parotid gland. |
| **Ogawa, 2006 [51]**  **Country:** Japan  **Aim**: To clarify the functional involvement of the trigeminal subnucleus interpolaris/caudalis transition region (Vi/Vc) and upper cervical spinal cord (C1/C2) in processing nociceptive input relevant to parotitis. | **Population mammals:**Rats  **Mean Age:** Adult  **Sex:** Male  **Total sample size:**68  **Number of groups:**1 | **Type of stimulus:**  Mechanical and thermal  **Location of stimulus:**  To the most sensitive area of the mechanical cutaneous receptive field  **Location of response:**  Medulla; C1/2 spinal cord  **TCN:** Vi/Vc  **Cervical Spine:** C1/C2 | **Main outcome:** Peak firing frequency to the mechanical or thermal stimulus | **Direction:** Orofacial to cervical  **Main outcome**: Eight of 23 Vi/Vc neurons responded to mechanical distention of the parotid gland, whereas no C1/C2 neurons responded to the parotid distention; Mechanical and cold responses increased significantly in C1/C2 but not Vi/Vc neurons following capsaicin; At the Vi/Vc transition region, pinch-evoked activity increased in neurons | **Conclusions**: Acute inflammation of the parotid gland differentially affects nociceptive neurons in the Vi/Vc transition region and the C1/C2 region. These two regions in Vsp and upper cervical cord are involved in functionally distinct responses to inflammation of the parotid gland. |
| **Okamoto, 2007 [52]**  **Country:** Japan  **Aim:**To assess the contribution of central serotonin receptors to the craniofacial tissue nociception in naïve male rats. | **Population mammals:**Rats  **Mean Age:** NR  **Sex:** Male  **Total sample size:**NR  **Number of groups:**4 | **Type of stimulus:**  Electrical, chemical, mechanical  **Location of stimulus:**  Left masseter muscle  **Location of response:**  Vc and upper cervical (C1–C2) spinal cord  **TCN:** Vc; Vc/C2  **Cervical Spine:** C1–C2 | **Main outcome:**  Orofacial nocifensive behavioral activities  **Secondary outcomes:**  Fos immunohistochemistry*^2^ | **Direction:**Orofacial to cervical  **Main outcome**: Orofacial nocifensive behavioral activities evoked by formalin injection into the masseter muscle were significantly reduced by intra- cisternal administration of amphetamines.  **Secondary outcome:** Formalin-evoked deep-nociceptive unit discharges were significantly reduced after topical administration of amphetamine onto the Vc/C2 region compared with vehicle-treated group in the early and late phases. | **Conclusions**: The stimulus on the orofacial nociceptive area through serotonin receptors can reduce nociceptive neural activities in the Vc/C2 region. |
| **Okamoto, 2008 [53]**  **Country:** USA  **Aim:**To determine if high and low doses of estradiol replacement in ovariectomized female rats differentially affected the TMJ-evoked Fos-LI*^2^ responses in the caudal trigeminal brainstem nuclear complex, including the Vc/C1-2 junction, and second, to determine if amino acid derivative and glutamate  receptor-mediated modulation of the Fos-LI*^2^ responses evoked by TMJ stimulation were  influenced by estradiol replacement. | **Population mammals:**Rats  **Mean Age:** Adult  **Sex:**  Female  **Total sample size:**40  **Number of groups:** 3 (1: high (HE2) dose, 2: low (LE2) dose, 3: control) | **Type of stimulus:**  Chemical  **Location of stimulus:**  TMJ  **Location of response:**  Vc/C1-2 junction  **TCN:** Trigeminal subnucleus caudalis (Vc); ventral trigeminal interpolaris/caudalis transition region (Vi/Vcvl)  **Cervical Spine:** Upper cervical spinal cord | **Main outcome:**  Fos Immunocytochemistry | **Direction:** Orofacial to Cervical  **Main outcome**: TMJ-evoked Fos LI in laminae I-II at Vc/C1-2 junction and the dorsal paratrigeminal region was significantly greater in HE2 than LE2 rats, while Fos-LI produced at the Vi/Vcvl was similar. E2 treatment also modified the influence of amino acid derivate and glutamate receptor antagonists on TMJ-evoked Fos-LI. The amino acid derivate antagonist dose-dependently reduced the Fos-LI response at the Vc/C1-2 junction in HE2 rats, while only high dose amino acid derivate-antagonist was effective in LE2 rats. Amino acid derivate-antagonist reduced equally the Fos-LI response at the Vi/Vc transition in both groups, while only minor effects were seen at the paratrigeminal nucleus region. The glutamate receptor antagonist, reduced Fos-LI at the Vc/C1-2 and Vi/Vcvl regions in HE2 rats, while only high dose glutamate receptor antagonist was effective in LE2 rats. | **Conclusions**: These results suggest that the stimulus on the TMJ nociceptive area has an influence on the nociceptive processing at the Vc/C1-2 junction mediated, in part, through ionotropic glutamate receptor-dependent mechanisms. |
| **Okamoto, 2005 [54]**  **Country:** Japan  **Aim:** To investigate the roles of ion-channel receptors of Vc/C2 region in neural activities recorded from superficial laminae of Vc/C2 region evoked by chemical stimulation to masseter muscle or noxious mechanical stimulation to facial skin, and those in orofacial nocifensive behavioral activities evoked by the injection of formalin into masseter muscle during TMJ inflammation. | **Population mammals:** Rats  **Mean Age:** NR  **Sex:** Male  **Total sample size:**68  **Number of groups:**2 (1: CFA, 2: non-CFA) | **Type of stimulus:**  Mechanical, chemical  **Location of stimulus:**  TMJ  **Location of response:**  Trigeminal subnucleus caudalis/upper cervical spinal cord junction  **TCN:** Trigeminal subnucleus caudalis  **Cervical Spine:** C1/C2 | **Main outcome:**  Neuronal activity at the TCN level | **Direction:**Orofacial to cervical  **Main outcome**: There is one improvement on the neural activity at the level of the Vc/C2 junction region relevant for TMD pain, after the stimulus. | **Conclusions**: The central serotonergic circuits modulate orofacial nociceptive processing through ion-channel receptors facilitates neural activity at the Vc/C2 region. |
| **Panneton, 1991 [55]**  **Country:** USA  **Aim:**To investigate the primary Afferent Projections From the Upper Respiratory Tract in the Muskrat. | **Population mammals:**Rats  **Mean Age:** Adult  **Sex:** NR  **Total sample size:**NR  **Number of groups: 1** | **Type of stimulus:**  Mechanical  **Location of stimulus:**  Upper respiratory tract  **Location of response:**  Subnucleus interpolaris; oralis and obex  **TCN:** ventromedial portions of the principal nucleus, subnucleus oralis and interpolaris  **Cervical Spine:**  Cervical dorsal horn | **Main outcome:**  Central projections of the sensory fibers innervating the upper respiratory tract | **Direction:**Orofacial to cervical  **Main outcome**: Convergence of primary afferent fibers innervating the upper respiratory tract of the muskrat into the paratrigeminal nuclei and lamina I of the medullary dorsal horn was verified. Numerous double-labeled cells were observed in the left V ganglion following HRP injections into the left supraorbital nerve or midline scalp, and DY injections into the right medullary and cervical dorsal horns. | **Conclusions**: There is a convergence of primary afferent fibers innervating the upper respiratory tract of the muskrat into the paratrigeminal nuclei and lamina I of the medullary dorsal horn. |
| **Park, 2016 [56]**  **Country:** USA  **Aim:**To assess whether there were detectable changes in excitatory or inhibitory synapses in the afferent recipient zone of the TNC complex in a rat model of chronic constriction injury of the infraorbital nerve (CCI-ION), which leads to orofacial neuropathic pain. | **Population mammals:**Rats  **Mean Age:** Adult  **Sex:** Male  **Total sample size:**6  **Number of groups:**2 (operation group; sham-operation) | **Type of stimulus:**  Mechanical  **Location of stimulus:**  Infraorbital nerve/face  **Location of response:**  Vc/c2  **TCN:** Vc/C2  **Cervical Spine:** C1/C2 | **Main outcome:** transmission electron microscopy was used to examine synaptic profiles | **Direction:**Orofacial to cervical  **Main outcome**:  Profile counts for R synapses were similar across groups. Counts for F synapses were higher ipsilateral to the CCI-ION injury than in the other groups. | **Conclusions**: These findings support that the rats with orofacial pain states had increased numbers and decreased mean synaptic length of R-profiles within the Vc/C2 superficial dorsal horn (lamina I) three-weeks post CCI-ION. Increases in the number of excitatory synapses in the superficial dorsal horn of Vc/C2. |
| **Puri, 2011 [57]**  **Country: USA**  **Aim:**To determine genes that are modulated by physiological levels of estradiol that could have a role in TMJ pain. | **Population mammals:**Rats  **Mean Age:** NR  **Sex:** Female  **Total sample size:**NR  **Number of groups:**4 | **Type of stimulus:**  Chemical  **Location of stimulus:**  TMJ  **Location of response:**  TMJ, trigeminal ganglia, Vc/C1–2  **TCN:** Trigeminal subnucleus caudalis  **Cervical Spine:** Upper cervical cord junction | **Main outcome:**  Estrogen effects on transcript expression. | **Direction:**Orofacial to cervical  **Main outcome**: Estradiol induced changes in gene expression within the TMJ, trigeminal ganglion and Vc/C1–2. | **Conclusions**: To conclude, plasma levels of estrogen in female rats modulate gene expression in the joint,  trigeminal ganglion and subnucleus caudalis region. |
| **Qu, 2020 [58]**  **Country:** China  **Aim:**To demonstrate the efficacy of electrical acupuncture in preventing migraine attacks by stimulating the acupoint GB20, which is located in the upper cervical tissues. | **Population mammals:**Rats  **Mean Age:** 12 weeks  **Sex:** Male  **Total sample size:**48  **Number of groups:**6 | **Type of stimulus:**  Mechanical, chemical  **Location of stimulus:**  Mechanical: face and cervical; Chemical: dura mater  **Location of response:**  Dura mater, tcc/c1 region  **TCN:** Subnucleus caudalis  **Cervical Spine:** C1 neurons | **Main outcome:**  activities of the neurons at the C1 level. | **Direction:**Cervical to orofacial/Orofacial to cervical  **Main outcome**: The activation of the GB20 through electrical acupuncture can reduce the active sate of the TCC neurons while preventing the onset of migraine. | **Conclusions**: There is one reduction of the discharge frequency of neurons in the TCC after stimulus. |
| **Sabino, 2002 [59]**  **Country:** USA  **Aim:**To better understand the neurons in the trigeminal complex and spinal cord that are activated following nociceptive stimuli to the orofacial complex. | **Population mammals:**Rats  **Mean Age:** NR  **Sex:** Male  **Total sample size:**15  **Number of groups:**1 (Tooth extraction) | **Type of stimulus:**  Mechanical  **Location of stimulus:**  Tooth  **Location of response:**  Brainstem and cervical spinal cord  **TCN:** Nuclei interpolaris and caudalis  **Cervical Spine:** Vi/Vc | **Main outcome:**  Internalization of the Substance P receptors in lamina I | **Direction:**Orofacial to cervical  **Main outcome**: SPR expression was observed in neurons located in the principal trigeminal nucleus (Pr5), SNo, and SNc, and the transition zone between SNc and SNi (Vi/Vc); Substance P receptors were rapidly internalized into the cytoplasm of lamina I and III–V neurons located in SNc and cervical spinal cord. | **Conclusions**: Following relatively minor tissue injury neurons showing Substance P receptor internalization were confined to the nucleus caudalis while procedures which cause greater tissue injury, neurons showing Substance P receptor internalization extended from the interpolaris/caudalis transition zone through the C7 spinal level. |
| **Sato, 2005 [60]**  **Country:** Japan  **Aim:**To examine the anatomical relationship between nociceptive neurons in the Vi/Vc and Vc/C1,2 zones through fluorogold retorgade tracing combined with Fos expression following TMJ inflammation. | **Population mammals:**Rats  **Mean Age:** Adult  **Sex:** Male  **Total sample size:**15  **Number of groups:**1 | **Type of stimulus:**  Mechanical  **Location of stimulus:**  Left TMJ  **Location of response:**  Medulla and upper cervical cord  **TCN:** Vc/C1-C2 and Vi/Vc  **Cervical Spine:** Upper cervical spinal cord | **Main outcome:** number of FG cells and Fos/FG cells | **Direction:**Orofacial to cervical  **Main outcome**: Numerous Fos protein-Li cells were present in both the Vi/Vc transition zone and the laminated Vc/C1-2 zone. Many neurons in the deep and superficial laminae exhibited Fos protein immunoreactivity after TMJ inflammation. | **Conclusions**: The information from the injured TMJ is specifically relayed to the Vi/Vc transition zone via the caudal laminated Vc/C1-2 zone. |
| **Sessle, 1986 [61]**  **Country:** USA  **Aim:**To determine the presence and extent of convergent inputs from oral-facial, tooth pulp, neck, muscle and visceral afferents to the neurons of the medullary dorsal horn. | **Population mammals:**Cats  **Mean Age:** Adult  **Sex:** NR  **Total sample size:** 15  **Number of groups:** 6 | **Type of stimulus:**  Mechanical, thermal, electrical  **Location of stimulus:**  Facial skin, oral mucosa, canine and premolar tooth pulp, laryngeal mucosa, cervical skin and muscle, jaw and tongue muscles, and tactile  **Location of response:**  Trigeminal (V) subnucleus caudalis  **TCN:** Vc  **Cervical Spine:** NR | **Main outcome:** Extracellular recordings of the activity of single neurons | **Direction:**Orofacial to cervical  **Main outcome**: Extensive convergence of afferent inputs, including inputs from skin or mucosal areas outside the neuronal oral-facial receptive field delineated by natural stimuli, was a particular feature of the units classified as cutaneous nociceptive neurons. On the basis of antidromic activation, 15% of these WDR and NS neurons were shown to have a direct projection to the contralateral thalamus. | **Conclusions**: there is a considerable proportion of neurons that have additional inputs from afferents supplying jaw and tongue muscles, larynx, neck, and canine or premolar tooth pulps, as well as from cutaneous or mucosal V afferents outside the mechanoreceptive field of the neurons. |
| **Shibuta, 2012 [62]**  **Country:** Japan  **Aim:**To clarify whether peripheral Glutamate receptors may be involved in the central sensitization of Vc and C1-C2 neurons activated by noxious heat or cold stimulation of these orofacial tissues. | **Population mammals:**Rats  **Mean Age:** NR  **Sex:** NR  **Total sample size:**408  **Number of groups:**2 (Glutamate or vehicle injection) | **Type of stimulus:**  Heat and cold  **Location of stimulus:**  Tongue or whisker pad skin  **Location of response:**  Vc and C1-C2 neurons  **TCN:** Vc and C1-C2 neurons  **Cervical Spine:** Splenius capitis muscle | **Main outcome:**  ERK phosphorylation*^1^ | **Direction:**Orofacial to cervical  **Main outcome**: ERK phosphorylation in Vc and C1-C2 neurons was detected within 2 min and peaked at 5 min following subcutaneous Glu injection; pERK-IR neurons expressed in Vc and C1-C2 after Glutamate injection was smaller in inhibitor-injected rats compared to that of vehicle-injected rats; The number of pERK-IR neurons also peaked at the obex level after cold stimulation (5 °C) of the tongue following submucosal Glutamate (0.5 M) injection into the tongue. | **Conclusions**: Glutamate application to the tongue or whisker pad skin caused an enhancement of head-withdrawal reflex and ERK phosphorylation in Vc and C1-C2 neurons to heat stimulation |
| **Shigenaga, 1998 [63]**  **Country:** Japan  **Aim:**To examine the central projections of the masticatory muscle afferents to the trigeminal sensory nuclear complex by the method of **t**ransganglionic transport of horseradish peroxidase wheat germ agglutinin **(**HRP-WGA) *^3^ conjugate. | **Population mammals:**Cats  **Mean Age:** NR  **Sex:** Female  **Total sample size:**21  **Number of groups:**5 | **Type of stimulus:**  Chemical  **Location of stimulus:**  Jaw-closing muscles  **Location of response:**  Brainstem, cervical cord and trigeminal ganglia  **TCN:** Brainstem and trigeminal ganglia  **Cervical Spine:** Cervical cord | **Main outcome:** Number of labelled cells | **Direction:** Orofacial to cervical  **Main outcome**: Projections of the muscle afferents of ganglionic origin to the trigeminal sensory nuclear complex were confined primarily to the caudal half of pars interpolaris (Vi), and the medullary and upper cervical dorsal horns. | **Conclusions**: After a stimulus of the orofacial nerve, an improvement on the afferents of the cervical area were identified. |
| **Shigenaga, 1986 [64]**  **Country:** Canada  **Aim:**To evaluate mechanisms that may underlie the sensitization of Vc and C1-C2 nociceptive neurons to heat, cold and mechanical stimuli following topical capsaicin treatment of the rat's facial skin by assessing, nocifensive behaviors as well as pERK*^1^ in Vc and C1-C2 neurons. | **Population mammals:**Rats  **Mean Age:** NR  **Sex:** NR  **Total sample size:**NR  **Number of groups:** 3 | **Type of stimulus:**  Heat, cold and mechanical stimulus  **Location of stimulus:**  Facial skin  **Location of response:**  Vc and C1-C2 neurons  **TCN:** Vc  **Cervical Spine:** C1-C2 nociceptive neurons | **Main outcome:** pERK-LI cells | **Direction:**Orofacial to cervical  **Main outcome**:  pERK-LI cells were observed in Vc and C1-C2 within 2 min after cessation of heat stimulus and peaked at 4 min, and subsequently declined in number. pERK-LI cells were also observed in the superficial laminae of Vc and C1-C2 at 4 minutes after cold stimulus of the lateral facial skin in rats with vehicle or capsaicin treatment. pERK-LI cells were also observed in the superficial laminae of the Vc and C1-C2 at 4 min after non-noxious and noxious mechanical stimuli of the facial skin. | **Conclusions**: After a cold stimulus of the orofacial nerve, an improvement on the afferents cells of the cervical area were identified. |
| Shigenaga Y *et al.* [65] **1986**  **Country:** Japan  **Aim:** Transganglionic transport of horseradish peroxidase (HRP) was used to study the termination patterns of somatic afferent fibers innervating oral and facial structures within the main nucleus (Vp), oral nucleus (Vo), and interpolar nucleus (Vi). | **Population mammals:** Cats  **Mean Age:** NR  **Sex:** NR  **Total sample size:**54  **Number of groups:**1 | **Type of stimulus:** Chemical  **Location of stimulus:** Lingual, buccal, inferior alveolar, superior alveolar, pterygopalatine, auriculotemporal, mylohyoid, anterior branch of mental, posterior branch of mental, zygomatic, infraorbital, frontal nerves  **Location of response:** Upper cervical cord  **TCN:** Main nucleus (Vp), oral nucleus (Vo), and interpolar nucleus (Vi)  **Cervical Spine:** Upper cervical cord | **Main outcome:**  Transganglionic transport of horseradish peroxidase (HRP) | **Direction:**Cervical to orofacial  **Main outcome**: HRP-labeled terminals of the primary afferents that in- nervate the intraoral structures were seen continuously through the dorsomedial regions of the ipsilateral trigeminal sensory nuclear complex (TSNC), i.e., the principal nucleus, pars oralis and pars interpolaris. Thus, the labeled terminal fields formed a rostrocaudally running column, although the density of afferent projections slightly varied from one rostrocaudal level of the TSNC to the next. | **Conclusions**: The relationship between the functional segregation and the cytoarchitectonic differentiation of the TSNC is discussed, particularly with respect to this somatotopic organization, combined with the characteristics of projecting cells in the TSNC. |
| **Shimizu, 2006 [66]**  **Country:** Japan  **Aim:**The pERK*^1^ and Fos*^1^ expression and masticatory muscle activity were analyzed in rats with capsaicin-induced acute inflammation of the tooth pulp in order to clarify the role of the spinal trigeminal nucleus and upper cervical spinal cord in tooth pulp pain. | **Population mammals:**Rats  **Mean Age:** NR  **Sex:** Male  **Total sample size:**35  **Number of groups: 7** | **Type of stimulus:**  Chemical  **Location of stimulus:**  Tooth pulp  **Location of response:**  Medullary and upper cervical cord neurons  **TCN:** Medullary  **Cervical Spine:** Upper cervical spinal cord | **Main outcome:**  Masticatory muscle activity | **Direction:**Orofacial to cervical  **Main outcome**: Digastric and masseteric muscle activities  were significantly increased following capsaicin injection into the molar tooth pulp but not  after vehicle treatment. | **Conclusions**: The present findings suggest that tooth-pulp-driven neurons in the spinal trigeminal nucleus are involved in tooth pulp pain through activation of the intracellular signal transduction pathway that involves earlier ERK phosphorylation and subsequent Fos expression. |
| **Strassman and Vos, 1993 [67]**  **Country:** USA  **Aim:**The distribution of fos-like-immunoreactivity*^2^ (fos-LI) in the medullary and upper cervical  dorsal horn was examined following noxious facial stimulation. | **Population mammals:**Rats  **Mean Age:** NR  **Sex:**  Male  **Total sample size:**63  **Number of groups: 2** (1-stimulated; 2-non-stimulated) | **Type of stimulus:**  Electrical, mechanical, chemical, thermal  **Location of stimulus:**  Mechanical: Mandibular, ophthalmic, maxillary and mandibular intraoral site, tongue; cornea. Electrical: infraorbital nerve; Thermal: supraorbital vibrissae; Chemical: subcutaneous  **Location of response:**  Medullary dorsal horn and nucleus interpolaris, C1, C2  **TCN:** Medullary dorsal horn and nucleus interpolaris (Vi)  **Cervical Spine:** C1, C2 | **Main outcome:**  fos-LI mapping | **Direction:**Orofacial to cervical  **Main outcome**: Pinch of different facial sites produced labeling in the ipsilateral dorsal horn whose distribution varied with the rostrocaudal and dorsoventral position of the facial stimulation site. The cornea was exceptional among the facial stimulation sites in that it had a specific representation at two distinct rostrocaudal levels, in C1 and the interpolaris-caudalis transition region; The proportion of labelling in laminae III-IV relative to laminae 1–11 was higher with noxious mechanical stimulation than with noxious thermal or chemical. The proportion of labeling in laminae III-IV produced by electrical stimulation of the infraorbital nerve was no greater than that produced by pinch. | **Conclusions**: The stimulus in the orofacial area, improve the input at the rostrocaudal levels, in C1 and the interpolaris-caudalis transition region. |
| **Strassman, 1994 [68]**  **Country:** USA  **Aim:**To investigate possible differences in the distribution of central neurons activated by afferent pathways from cutaneous and deep tissues that may be related to the differing quality of the sensations they evoke. | **Population mammals:**Rats  **Mean Age:** Adult  **Sex:** Male  **Total sample size:**NR  **Number of groups:**2 (control and stimulation) | **Type of stimulus:**  Mechanical  **Location of stimulus:**  Transverse/sagittal sinus  **Location of response:**  Upper cervical and medullary dorsal horna;  **TCN:** Medullary dorsal horna  **Cervical Spine:** Upper cervical | **Main outcome:**  Distribution of fos-LI-labeled cells*^2^ | **Direction:**Cervical to orofacial  **Main outcome**: Transverse sinus stimulation increased the number of fos-LI cells above control in the dorsal horn and the Vi/Vc region on the side ipsilateral to the stimulus. Sagittal sinus stimulation produced smaller increases in labeling above control in the dorsal horn and Vi/Vc. The labeling produced by transverse sinus stimulation was significantly greater than that produced by sagittal sinus stimulation in both the dorsal horn and Vi/Vc. | **Conclusions**: Fos-LI mapping can be a useful method for the investigation of somatotopy but is subject to serious limitations when used for the investigation of laminar organization. The interpolaris-caudalis transition region may have properties that are distinct from those of the rest of the trigeminal complex, possibly related to an involvement in autonomic function. |
| **Sugimoto, 1994 [69]**  **Country:** Japan  **Aim:**To demonstrate the transsynaptic activation of sensory neurons in the subnucleus caudalis by noxious mechanical stimulation of maxillary and mandibular oral mucous membranes. | **Population mammals:**Rat  **Mean Age:** NR  **Sex:** NR  **Total sample size:**25  **Number of groups:** 1 | **Type of stimulus:**  Mechanical  **Location of stimulus:**  First molar, tongue, perifacial skins, upper and lower eyelid, lateral eye angle, the submental area  **Location of response:**  Caudal medulla and cervical spinal cord  **TCN:** Subnucleus caudalis  **Cervical Spine:** Spinal cord | Main outcome:  Topographic distribution of neurons with c-fos*^2^ like immunoreactivity | **Direction:**Orofacial to cervical  **Main outcome**: Fos-neurons following stimulation of the cutaneous receptive field were found in laminae I and II of the first and second cervical segments of the spinal cord ipsilateral to the stimulation and exhibited a clear somatotopic segregation; *i.e.*, the fos-neurons responding to receptive fields in the mandibular, maxillary, and ophthalmic divisions were arranged in a mediolateral sequence. Fos-neurons responding to the oral mucous membrane were found in the similar laminae of the rostral pole of subnucleus caudalis bilaterally. | **Conclusions**: The stimulus in the orofacial area results in a clear somatotopic segregation in laminae I and II of the first and second cervical segments of the spinal cord ipsilateral to the stimulation. |
| **Sugimoto, 1997 [70]**  **Country:** Japan  **Aim:**To analyze the central projection of CGRP and SP trigeminal primary neurons to the lower brainstem (the brainstem sensory trigeminal nuclear complex and surrounding structures) of rats. | **Population mammals:**Rats  **Mean Age:** Adult  **Sex:** Male  **Total sample size:**13  **Number of groups:**3 (Normal; unilateral trigeminal rhizotomy; Trigeminal tractotomy alone or a combined and upper cervical hemicordotomy) | **Type of stimulus:** Chemical  **Location of stimulus:**  Lower brainstem  **Location of response:**  Lower brainstem  **TCN:** Vi and paratrigeminal nucleus (paraV), Vo and principal sensory trigeminal nucleus (PrV)  **Cervical Spine:** Upper cervical cord | **Main outcome:**  Distribution of CGRP-like immunoreactivity and SP in the lower brainstem | **Direction:**Unclear  **Main outcome**: The glossopharyngeal and vagal primaries are candidates for the source of CGRP-ir projection to the Vo and the medullary dorsal horn, while the dorsal root axons supply the medullary dorsal horn with CGRP-ir terminals. Contralateral primary neurons crossing the midline appear to contain CGRP and to terminate in the medullary dorsal horn. | **Conclusions**: The CGRP-ir trigeminal primary neurons projected to the entire rostrocaudal extent of the ipsilateral brainstem sensory trigeminal nuclear complex. |
| **Suzuki, 2008 [71]**  **Country:** Japan  **Aim:**To clarify the effect of age-related change in trigeminal nociception. | **Population mammals:**Rats  **Mean Age:** 9–12-month-old; 29–34-month old  **Sex:** NR  **Total sample size:**20  **Number of groups:**2 (9–12 and 29–34-month old rats) | **Type of stimulus:**  Chemical  **Location of stimulus:**  Whisker pad  **Location of response:**  Vc and upper cervical spinal cord neurons  **TCN:** Vc  **Cervical Spine:** Upper cervical spinal cord | **Main outcome:** Phosphorylation of extra-cellular signal-regulated kinase (pERK)*^1^ | **Direction:**Orofacial to cervical  **Main outcome**: The number of pERK-LI cells was slightly, but not significantly larger in aged rats compared with that of adults. Pretreatment with naloxone significantly increased the number of capsaicin-induced pERK- LI cells in adult rats but not in aged rats. | **Conclusions**: The present findings suggest that the descending modulation system impaired with advancing age, resulting in the abnormal pain sensation in aged rats |
| **Takemura, 1987 [72]**  **Country:** Japan  **Aim:**To determine the exact central projections of peripheral branches of the trigeminal nerve for understanding the underlying functional segregation of primary neurons compromising them. | **Population mammals:**Rats  **Mean Age:** NR  **Sex:** Male  **Total sample size:** 33  **Number of groups:** 6 | **Type of stimulus:**  Chemical  **Location of stimulus:**  TMJ/auricular region  **Location of response:**  Brainstem and cervical segments of spinal cord  **TCN:** Trigeminal nucleus principalis, subnuclei oralis and interpolaris  **Cervical Spine:** C1/2/3 | **Main outcome:** Projectional areas of each branch. | **Direction:** Orofacial to cervical  **Main outcome**: Primary neurons innervating the intraoral structures project to the nucleus of the solitary tract and the supra and paratrigeminal nuclei, whereas those innervating the facial skin do not. Primary neurons innervating the periphery of the face project to the spinal dorsal horn and those innervating the intra perioral region project to medullary dorsal horn, though this segregation from the medulla to the C3 segment is loose. Trigeminal primary neurons project to the contralateral dorsal horn from the medulla to the C3 segment. | **Conclusions**: The contralateral projection was reported at the cervical segment after a chemical stimulus at the alveolar nerve on TMJ. |
| **Takeshita, 2001 [73]**  **Country:** USA  **Aim:**To determine if TMJ-responsive neurons encoded the intensity of pro-inflammatory chemical signals. | **Population mammals:**Rats  **Mean Age:** NR  **Sex:** Male  **Total sample size:**NR  **Number of groups:**4 (phosphate buffered saline, glutamate, bradykinin,  or mustard oil) | **Type of stimulus:**  Electrical, mechanical, chemical  **Location of stimulus:** Mechanical: TMJ, skin, condyle, temporalis muscle; Chemical: TMJ  **Location of response:**  Vc; C1, C2  **TCN:** Vc  **Cervical Spine:** C1, C2 | **Main outcome:**  Fos-LI mapping*^2^ | **Direction:**Orofacial to cervical  **Main outcome**: Cornea was exceptional among the facial stimulation sites in that it had a specific representation C1 and the interpolaris-caudalis transition region; The proportion of labelling in laminae III-IV relative to laminae I-II was higher with noxious mechanical stimulation than with noxious thermal (55°C) or chemical stimulation. The proportion of labelling in laminae III-IV produced by electrical stimulation of the infraorbital nerve was no greater than that produced by pinch. | **Conclusions**: Just the mechanical stimulus at the orofacial region can influence the proportion of labelling at the Vc and C1-2 level. |
| **Tanimoto, 2004 [74]**  **Country:** Japan  **Aim:**To test the hypothesis that vagal afferent stimulation modulates the first cervical dorsal horn neuron activity, which is projected by tooth pulp afferent inputs through the activation of a local ion-channel receptor system. | **Population mammals:**Rats  **Mean Age:** NR  **Sex:** Male  **Total sample size:**31  **Number of groups:** 1 | **Type of stimulus:**  Electrical, mechanical  **Location of stimulus:**  Pulp of upper incisors  **Location of response:**  C1  **TCN:** NR  **Cervical Spine:** C1 | **Main outcome:**  Extracellular Recording of C1 Spinal Neuron Activity. | **Direction:**Orofacial to cervical  **Main outcome**: Tooth pulp stimulation evoked C1 spinal neuron excitation was inhibited by vagal afferent stimulation. Application of glutamate produced a maximal increase in the C1 spinal neuron activity at a minimal current. In 81.5% neurons vagal afferent conditioning stimulation caused an inhibition of the glutamate application evoked C1 spinal neuron excitation. | **Conclusions**: These results suggest that vagal afferent stimulation-induced suppression of C1 spinal neuron activity, responding to tooth pulp stimulation, ion-channel activation. |
| **Tanimoto, 2002 [75]**  **Country:** Japan  **Aim:** To test the hypothesis that vagal afferent inputs modify the tooth pulp stimulation-evoked activity of the C1 neurons via the activation of endogenous noradrenergic and serotonergic systems. | **Population mammals:** Rats  **Mean Age:** Adult  **Sex:** Male  **Total sample size:**30  **Number of groups:**1 | **Type of stimulus:**  Electrical, mechanical  **Location of stimulus:**  Pulp of upper incisors  **Location of response:**  C1  **TCN:** NR  **Cervical Spine:** C1 | **Main outcome:**  Recordings of the digastric muscle electromyogram  and C1 spinal neuronal activity | **Direction:**Orofacial to cervical  **Main outcome**: Activity of C1 spinal neurons and the amplitude in a digastric muscle electromyogram increased proportionally during tooth pulp stimulation. Activity in 82.1% of these C1 neurons was suppressed by vagal afferent stimulation of the right vagus nerve. Suppressive effects of vagal afferent stimulation on C1 spinal neuron activity were significantly reduced after intravenous administration of adrenergics receptor. | **Conclusions**: These results suggest that vagal afferent stimulation inhibits nociceptive transmission in the C1 spinal neuron activity via the activation of both noradrenergic and serotonergic descending inhibitory systems, from the stimulus of the pulp of upper incisors. |
| **Tashiro, 2009 [76]**  **Country:** USA  **Aim:** The present study tested the hypothesis that estrogen status acts through the MAPK/ERK*^1^ signaling pathway to alter TMJ nociceptive processing. | **Population mammals:**Rats  **Mean Age:** Adult  **Sex:** Female  **Total sample size:**30  **Number of groups:**2 (Naïve; CFA) | **Type of stimulus:**  Chemical  **Location of stimulus:** TMJ  **Location of response:** Spinomedullary junction  **TCN:** Vc  **Cervical Spine:** C1-2 | **Main outcome:** Spike amplitude and shape | **Direction:**Orofacial to cervical  **Main outcome**: In naive rats, low dose MAPK/ERK inhibitor caused a maximal inhibition of ATP-evoked activity, whereas even high doses had only minor effects on units in treatment rats. By contrast, after chronic TMJ inflammation, MAPK/ERK inhibitor produced a marked and similar dose-related inhibition of ATP-evoked activity in high and low-dose rats. | **Conclusions**: These data suggested that E2 and inflammation acted, at least in part, through a common MAPK/ERK signaling pathway to enhance the activity of TMJ-responsive units’ laminae I–II at the Vc/C1–2. |
| **Yasuda, 1995 [77]**  **Country:** Japan  **Aim:**To determine the distribution of trigeminal mesencephalic nucleus innervating the anterior belly of the digastricus muscle and mylohyoid muscle, as well as that of the central projections of the afferent fibers of each branch of the mylohyoid nerve. | **Population mammals:**Rats  **Mean Age:** NR  **Sex:** NR  **Total sample size:**16  **Number of groups:**3 mylohyoid muscle, Digastricus muscle, and the cutaneous branch | **Type of stimulus:**  Chemical  **Location of stimulus:**  mylohoid muscle, digatricus muscle and cutaneous branch  **Location of response:** Superior colliculus and the upper cervical cord  **TCN:** Ipsilateral trigeminal nucleus caudalis  **Cervical Spine:** Upper cervical | **Main outcome:**  labeling by histochemical procedure for detection of HRP*^3^ activity | **Direction:**Orofacial to cervical  **Main outcome**: Projections of the primary afferents of the mylohoid muscle were observed in the ipsilateral trigeminal nucleus caudalis, the upper cervical dorsal horns of laminae 1-111, and the dorsolateral reticular formation, whereas the primary afferents of the digastricus muscle terminated in the ipsilateral trigeminal nucleus principals and reticular formation. | **Conclusions**: The main terminal fields of the mylohoid and digastricus afferent muscle fibers are in lamina I in the Vc and the upper cervical dorsal horn. |
| **Vos and Strassman, 1995 [78]**  **Country:** USA  **Aim:**Changes in patterns of nonevoked and mechanical stimulation-evoked fos*^2^ expression were quantified in rats that were tested for behavioral abnormalities indicative of trigeminal neuropathic pain after loose constrictive ligation of the infraorbital nerve. | **Population mammals:**Rats  **Mean Age:** NR  **Sex:** Male  **Total sample size:**24  **Number of groups:** 2 ((1) ION-CCI rats (2) Sham-operated rats) | **Type of stimulus:**  Mechanical  **Location of stimulus:** Hair  **Location of response:**  Medulla and upper cervical cord  **TCN:** Laminae I–II and laminae III–IV  **Cervical Spine:** Upper cervical cord | **Main outcome:**  Behavioral changes and number of Fos-Li cells | **Direction:** Orofacial to cervical  **Main outcome**: The number of fos-LI neurons in the contralateral medullary horn was not different among the six groups. | **Conclusions**:  The stimulus in the hair does not affect the number of Fos-Li cells at the cervical area. |
| **Young and Perryman, 1984 [79]**  **Country:** USA  **Aim:**We have monitored behavioral responses to electrical stimulation of the dental pulp and facial skin in monkeys before and after a variety of lesions in the trigeminal brain-stem complex and section of various cranial and spinal nerves. | **Population mammals:**Monkeys  **Mean Age:** NR  **Sex:** NR  **Total sample size:**11  **Number of groups:**6 | **Type of stimulus:**  Electrical  **Location of stimulus:**  Dental pulp and facial skin  **Location of response:**  Trigeminal brain-stem nuclear complex  **TCN:**nucleus caudalis, nucleus principalis, nucleus oralis, nucleus interpolaris  **Cervical Spine:** C-l-C-3 | **Main outcome:**  Thresholds for lever-pressing in response | **Direction:**Orofacial to cervical  **Main outcome**: Radiofrequency destruction of the trigeminal nuclei principalis, oralis, and interpolaris caused elevations of lever-pressing thresholds in response to dental pulp stimulation, and also smaller but statistically significant elevations on cutaneous electrical stimulation. Mild reductions in adversive responses to cutaneous pinscratch were also produced by these rostral nuclear lesions, suggesting analgesia. | **Conclusions**: Primary afferent fibers for cutaneous facial nociception are contained in the trigeminal, facial, glossopharyngeal, and vagus nerves, and the upper cervical dorsal and ventral roots. |
| **Zerari-Mailly, 2003 [80]**  **Country:** France  **Aim:**To identify neurons involved in the rat blink reflex (to specify the distribution pattern of activated neurons in the sensory trigeminal nuclei, the C1 cervical spinal cord, and the pontomedullary receptive field). | **Population mammals:**Rats  **Mean Age:** Adult  **Sex:** NR  **Total sample size:**11  **Number of groups:**2 stimulations + gold HRP*^3^ gold HRP + nerve dissection) | **Type of stimulus:**  Electrical  **Location of stimulus:**  Left SO nerve.  **Location of response:**  Vo/Vi/Vc-C2  **TCN:** Vo, Vi  **Cervical Spine:** Vc; C1-C2 | **Main outcome:**  Number of single-labeled c-Fos*^2^ neurons as well as double-labeled c-Fos/gold–HRP neurons | **Direction:**Cervical to orofacial/Orofacial to cervical  **Main outcome**: gold-HRP/c-Fos was in neurons of ventral pars caudalis layers I–IV and ventral pars interpolaris; C1: SO, stimulation - c-Fos neurons in laminae I–V; ffter additional injections in VII, the double-labeled c-Fos/gold-HRP neurons were concentrated in laminae IV and V; c-Fos neurons - throughout the pontomedullary reticular formation; c-Fos neurons - lateral portion of the dorsal medullary reticular field. | **Conclusions**: The present results demonstrate the existence of neurons in the Vi/Vc and C1 spinal cord that receive direct inputs from the eyelids and project to the SO nerve. |
| **Zhou, 1999 [81]**  **Country:** USA  **Aim:**To examine the effects of persistent orofacial deep versus cutaneous tissue injury on prolonged neuronal activation in the trigeminal nociceptive pathways; to analyze and understand the functional significance of Fos*^2^ induction in trigeminal as well as other medullary structures. | **Population mammals:** Rats  **Mean Age:** NR  **Sex:** NR  **Total sample size:**20  **Number of groups:**1 | **Type of stimulus:**  Chemical  **Location of stimulus:** Jaw  **Location of response:**  Caudal medulla and upper cervical cord  **TCN:** Trigeminal subnuclei interpolaris and caudalis  **Cervical Spine:** C1–C2 dorsal horn | **Main outcome:**  Fos-LI in the trigeminal nucleus | **Direction:**Orofacial to cervical  **Main outcome**: In laminated subnucleus caudalis and cervical dorsal horn, Fos-positive cells were mainly distributed in laminae I/II and V/VI; few Fos-labeled neurons were found in laminae III/IV, after stimulation in the jaw. | **Conclusions**: The stimulus at the jaw induces a distribution of Fos-positive cells were mainly distributed in laminae I/II and V/VI; few Fos-labeled neurons were found in laminae III/IV |
| **Westberg, 1991 [82]**  **Country:** Sweden  **Aim:**To illuminate the convergence profile on and the projection of the  NVspo-gamma, interneurons in further detail, in order to broaden our understanding of the significance of these neurones in the control of orofacial movements. | **Population mammals:** Cats  **Mean Age:** NR  **Sex:** Mixed  **Total sample size:**24  **Number of groups:** 1 | **Type of stimulus:** Electrical  **Location of stimulus:** Trigeminal tract (V) or the presynaptic potential in the different trigeminal sensory nuclei (main sensory trigeminal nucleus (NVsnpr) and oral nucleus of spinal trigeminal tract (NVspo-y))  **Location of response:** Masseteric, the digastric and in the dorsal neck motor nuclei  **TCN:** Trigeminal tract (V) or the presynaptic potential in the different trigeminal sensory nuclei (NVsnpr and NVspo-y)  **Cervical Spine:** Axon terminals within the neck muscle motoneurone pools | **Main outcome:** Functional localization of recording positions h~ the NVspo-T | **Direction:**Cervical to orofacial  **Main outcome**: It was found that the inter neurones, mainly recorded in the dorsal and dorsomedial aspect of the NVspo-gamma, receive short latency inputs from the low threshold oral and perioral afferents and longer latency inputs from the high threshold jaw and neck muscle afferents. There was evidence for convergence from the cervical segmental level (29%) and some of the neurones had axon terminals in the superior colliculus. | **Conclusions**:  The stimulus applied at the trigeminal tract trigger inputs at the orofacial region. |
| **Kubo, 2022 [83]**  **Country:** Japan  **Aim:** To establish a new rat model of craniofacial myalgia, and to clarify which central nervous system pathways are activated in the model. | **Population mammals:** Rats  **Mean Age:** 8–10 weeks old  **Sex:** Mixed  **Total sample size:**109  **Number of groups:**3 inserted), which was called the NGF group. | **Type of stimulus:** Electrical, mechanical  **Location of stimulus:** Trapezius and masseter muscles  **Location of response:** Masseter muscle  **TCN:** Ventral trigeminal interpolaris/caudalis transition zone (Vi/Vc)  **Cervical Spine:** Upper cervical cord | **Main outcome:**  Expression of pERK-IR*^1^ | **Direction:**Cervical to orofacial  **Main outcome**: The number of pERK-immunoreactive neurons in the brainstem was increased significantly in female rats in the group with both stimuli compared to rats in other groups with a single stimulus. Mechanosensitive MM-innervating neurons in the brainstem projected to the parabrachial nucleus. Morphine administration blocked the increase in the number of pERK-immunoreactive neurons in both the brainstem and parabrachial nucleus. | **Conclusions**: The number of pERK-immunoreactive neurons in the brainstem was increased significantly after the Trapezius and masseter muscles stimulation. |

*^1^ pERK/pERK-LI: is used to assess changes in immunohistochemistry by phosphorylating gene expression; pERK phosphorylates to pERK-LI;

*^2^ c-fos/Fos-LI: protein that is used as a marker for neuronal activity following depolarization; c-fos after depolarization Fos-LI;

*^3^ Horseradish perioxidase (HRP): tracer for immunohistochemistry by following the endocytic pathway.

ATP: Adenosintriphosphat; C1: Cervical vertebrae 1; C2: Cervical vertebrae 2; C3: Cervical vertebrae 3; CFA: Complete Freuds-Adjuvant; CGRP: Calcitonin gene-related peptide; HWT: Head-withdrawal-threshold; IO: Inferior orbital; ION: Infraorbital nerve; CCI: Chronic constriction injury; LTM: Low-threshold-mechanoreceptive; NS: Non-specific; NR: Not reported; SO nerve: Subociptal nerve; SP: Substance P; SSS: Sinus sagittalis superior; TMJ: Tempormandibular joint; VNS: Vagal nerve stimulation; WDR: Wide-dynamic-range; Vc: Spinal trigeminal nucleus caudalis; Vo: Spinal trigeminal nucleus oralis; Vi: Spinal trigeminal nucleus interpolaris; NVsnpr: main sensory trigeminal nucleus; NVspo-y: oral nucleus of the spinal trigeminal tract.

References

[1] Adachi K, Shimizu K, Hu JW, Suzuki I, Sakagami H, Koshikawa N, *et al*. Purinergic receptors are involved in tooth-pulp evoked nocifensive behavior and brainstem neuronal activity. Molecular Pain. 2010; 6: 59.

[2] Bereiter DA, Bereiter DF, Ramos M. Vagotomy prevents morphine-induced reduction in Fos-like immunoreactivity in trigeminal spinal nucleus produced after TMJ injury in a sex-dependent manner. Pain. 2002; 96: 205–213.

[3] Bereiter DA. Sex differences in brainstem neural activation after injury to the TMJ region. Cells Tissues Organs. 2001; 169: 226–237.

[4] Busch V, Jakob W, Juergens T, Schulte-Mattler W, Kaube H, May A. Functional connectivity between trigeminal and occipital nerves revealed by occipital nerve blockade and nociceptive blink reflexes. Cephalalgia. 2006; 26: 50–55.

[5] Casatti CA, Frigo L, Bauer JA. Origin of sensory and autonomic innervation of the rat temporomandibular joint: a retrograde axonal tracing study with the fluorescent dye fast blue. Journal of Dental Research. 1999; 78: 776–783.

[6] Chiaia NL, Hess PR, Hosoi M, Rhoades RW. Morphological characteristics of low-threshold primary afferents in the trigeminal subnuclei interpolaris and caudalis (the medullary dorsal horn) of the golden hamster. Journal of Comparative Neurology. 1987; 264: 527–546.

[7] Chibuzo GA, Cummings JF. The origins of the afferent fibers to the lingual muscles of the dog, a retrograde labelling study with horseradish peroxidase. Anatomical Record. 1981; 200: 95–101.

[8] Chudler EH, Foote WE, Poletti CE. Responses of cat C1 spinal cord dorsal and ventral horn neurons to noxious and non-noxious stimulation of the head and face. Brain Research. 1991; 555: 181–192.

[9] Classey JD, Knight YE, Goadsby PJ. The NMDA receptor antagonist MK-801 reduces Fos-like immunoreactivity within the trigeminocervical complex following superior sagittal sinus stimulation in the cat. Brain Research. 2001; 907: 117–124.

[10] Demartini C, Tassorelli C, Zanaboni AM, Tonsi G, Francesconi O, Nativi C, *et al*. The role of the transient receptor potential ankyrin type-1 (TRPA1) channel in migraine pain: evaluation in an animal model. Journal of Headache & Pain. 2017; 18: 94.

[11] Du BL, Li JN, Guo HM, Li S, Liu B. The effect of functional mandibular shift on the muscle spindle systems in head-neck muscles and the related neurotransmitter histamine. Journal of Craniofacial Surgery. 2017; 28: 1628–1634.

[12] Goadsby PJ, Zagami AS. Stimulation of the superior sagittal sinus increases metabolic activity and blood flow in certain regions of the brainstem and upper cervical spinal cord of the cat. Brain. 1991; 114: 1001–1011.

[13] Hathaway CB, Hu JW, Bereiter DA. Distribution of Fos-like immunoreactivity in the caudal brainstem of the rat following noxious chemical stimulation of the temporomandibular joint. Journal of Comparative Neurology. 1995; 356: 444–456.

[14] Honda K, Kitagawa J, Sessle BJ, Kondo M, Tsuboi Y, Yonehara Y, *et al*. Mechanisms involved in an increment of multimodal excitability of medullary and upper cervical dorsal horn neurons following cutaneous capsaicin treatment. Molecular Pain. 2008; 4: 59.

[15] Honda K, Noma N, Shinoda M, Miyamoto M, Katagiri A, Kita D, *et al*. Involvement of peripheral ionotropic glutamate receptors in orofacial thermal hyperalgesia in rats. Molecular Pain. 2011; 7: 75.

[16] Hu JW, Sessle BJ, Raboisson P, Dallel R, Woda A. Stimulation of craniofacial muscle afferents induces prolonged facilitatory effects in trigeminal nociceptive brain-stem neurones. Pain. 1992; 48: 53–60.

[17] Hu JW, Sun KQ, Vernon H, Sessle BJ. Craniofacial inputs to upper cervical dorsal horn: implications for somatosensory information processing. Brain Research. 2005; 1044: 93–106.

[18] Imbe H, Ren K. Orofacial deep and cutaneous tissue inflammation differentially upregulates preprodynorphin mRNA in the trigeminal and paratrigeminal nuclei of the rat. Brain Research Molecular Brain Research. 1999; 67: 87–97.

[19] Jacquin MF, Renehan WE, Mooney RD, Rhoades RW. Structure-function relationships in rat medullary and cervical dorsal horns. I. Trigeminal primary afferents. Journal of Neurophysiology. 1986; 55: 1153–1186.

[20] Jacquin MF, Rhoades RW. Central projections of the normal and ‘regenerate’ infraorbital nerve in adult rats subjected to neonatal unilateral infraorbital lesions: a transganglionic horseradish peroxidase study. Brain Research. 1983; 269: 137–144.

[21] Kamimura R, Hossain MZ, Unno S, Ando H, Masuda Y, Takahashi K, *et al*. Inhibition of 2-arachydonoylgycerol degradation attenuates orofacial neuropathic pain in trigeminal nerve-injured mice. Journal of Oral Science. 2018; 60: 37–44.

[22] Kato S, Papuashvili N, Okada YC. Identification and functional characterization of the trigeminal ventral cervical reflex pathway in the swine. Clinical Neurophysiology. 2003; 114: 263–271.

[23] Kaube H, Keay KA, Hoskin KL, Bandler R, Goadsby PJ. Expression of c-Fos-like immunoreactivity in the caudal medulla and upper cervical spinal cord following stimulation of the superior sagittal sinus in the cat. Brain Research. 1993; 629: 95–102.

[24] Kiyomoto M, Shinoda M, Honda K, Nakaya Y, Dezawa K, Katagiri A, *et al.* p38 phosphorylation in medullary microglia mediates ectopic orofacial inflammatory pain in rats. Molecular Pain. 2015; 11: 48.

[25] Kiyomoto M, Shinoda M, Okada-Ogawa A, Noma N, Shibuta K, Tsuboi Y, *et al*. Fractalkine signaling in microglia contributes to ectopic orofacial pain following trapezius muscle inflammation. Journal of Neuroscience. 2013; 33: 7667–7680.

[26] Kobayashi A, Shinoda M, Sessle BJ, Honda K, Imamura Y, Hitomi S, *et al.* Mechanisms involved in extraterritorial facial pain following cervical spinal nerve injury in rats. Molecular Pain. 2011; 7: 12.

[27] Kramer PR, Bellinger LL. Reduced GABAA receptor alpha6 expression in the trigeminal ganglion enhanced myofascial nociceptive response. Neuroscience. 2013; 245: 1–11.

[28] Kramer PR, Bellinger LL. Infusion of Gabralpha6 siRNA into the trigeminal ganglia increased the myogenic orofacial nociceptive response of ovariectomized rats treated with 17β-estradiol. Neuroscience. 2014; 278: 144–153.

[29] Kurose M, Imbe H, Nakatani Y, Hasegawa M, Fujii N, Takagi R, *et al*. Bilateral increases in ERK activation at the spinomedullary junction region by acute masseter muscle injury during temporomandibular joint inflammation in the rats. Experimental Brain Research. 2017; 235: 913–921.

[30] Lam DK, Sessle BJ, Hu JW. Surgical incision can alter capsaicin-induced central sensitization in rat brainstem nociceptive neurons. Neuroscience. 2008; 156: 737–747.

[31] Lam DK, Sessle BJ, Hu JW. Glutamate and capsaicin effects on trigeminal nociception II: activation and central sensitization in brainstem neurons with deep craniofacial afferent input. Brain Research. 2009; 1253: 48–59.

[32] Li KW, Yu YP, Zhou C, Kim DS, Lin B, Sharp K, *et al*. Calcium channel alpha2delta1 proteins mediate trigeminal neuropathic pain states associated with aberrant excitatory synaptogenesis. Journal of Biological Chemistry. 2014; 289: 7025–7037.

[33] Li CX, Yang Q, Vemulapalli S, Waters RS. Forelimb amputation-induced reorganization in the cuneate nucleus (CN) is not reflected in large-scale reorganization in rat forepaw barrel subfield cortex (FBS). Brain Research. 2013; 1526: 26–43.

[34] Liu MG, Matsuura S, Shinoda M, Honda K, Suzuki I, Shibuta K, *et al*. Metabotropic glutamate receptor 5 contributes to inflammatory tongue pain via extracellular signal-regulated kinase signaling in the trigeminal spinal subnucleus caudalis and upper cervical spinal cord. Journal of Neuroinflammation. 2012; 9: 258.

[35] Luo P, Wong R, Dessem D. Projection of jaw-muscle spindle afferents to the caudal brainstem in rats demonstrated using intracellular biotinamide. Journal of Comparative Neurology. 1995; 358: 63–78.

[36] Luz LL, Fernandes EC, Dora F, Lukoyanov NV, Szucs P, Safronov BV. Trigeminal Aδ- and C-afferent supply of lamina I neurons in the trigeminocervical complex. Pain. 2019; 160: 2612–2623.

[37] Lyubashina OA, Sokolov AY, Panteleev SS. Vagal afferent modulation of spinal trigeminal neuronal responses to dural electrical stimulation in rats. Neuroscience. 2012; 222: 29–37.

[38] Lyubashina OA, Panteleev SS, Sokolov AY. Inhibitory effect of high-frequency greater occipital nerve electrical stimulation on trigeminovascular nociceptive processing in rats. Journal of Neural Transmission. 2017; 124: 171–183.

[39] Marfurt CF, Turner DF. The central projections of tooth pulp afferent neurons in the rat as determined by the transganglionic transport of horseradish peroxidase. Journal of Comparative Neurology. 1984; 223: 535–547.

[40] Marfurt CF. The central projections of trigeminal primary afferent neurons in the cat as determined by the tranganglionic transport of horseradish peroxidase. Journal of Comparative Neurology. 1981; 203: 785–798.

[41] Margolis TP, LaVail JH, Setzer PY, Dawson CR. Selective spread of herpes simplex virus in the central nervous system after ocular inoculation. Journal of Virology. 1989; 63: 4756–4761.

[42] Matsumoto S, Takeda M, Tanimoto T. Effects of electrical stimulation of the tooth pulp and phrenic nerve fibers on C-1 spinal neurons in the rat. Experimental Brain Research. 1999; 126: 351–358.

[43] Miyamoto M, Tsuboi Y, Takamiya K, Huganir RL, Kondo M, Shinoda M, *et al*. Involvement of GluR2 and GluR3 subunit C-termini in the trigeminal spinal subnucleus caudalis and C1-C2 neurons in trigeminal neuropathic pain. Neuroscience Letters. 2011; 491: 8–12.

[44] Morch CD, Hu JW, Arendt-Nielsen L, Sessle BJ. Convergence of cutaneous, musculoskeletal, dural and visceral afferents onto nociceptive neurons in the first cervical dorsal horn. European Journal of Neuroscience. 2007; 26: 142–154.

[45] Nakajima A, Tsuboi Y, Suzuki I, Honda K, Shinoda M, Kondo M, *et al.* PKCgamma in Vc and C1/C2 is involved in trigeminal neuropathic pain. Journal of Dental Research. 2011; 90: 777–781.

[46] Nishimori T, Sera M, Suemune S, Yoshida A, Tsuru K, Tsuiki Y, *et al*. The distribution of muscle primary afferents from the masseter nerve to the trigeminal sensory nuclei. Brain research. 1986; 372: 375–381.

[47] Noma N, Tsuboi Y, Kondo M, Matsumoto M, Sessle BJ, Kitagawa J, *et al.* Organization of pERK-immunoreactive cells in trigeminal spinal nucleus caudalis and upper cervical cord following capsaicin injection into oral and craniofacial regions in rats. Journal of Comparative Neurology. 2008; 507: 1428–1440.

[48] Noma N, Watanabe K, Sato Y, Imamura Y, Yamamoto Y, Ito R, *et al*. Botulinum neurotoxin type A alleviates mechanical hypersensitivity associated with infraorbital nerve constriction injury in rats. Neuroscience Letters. 2017; 637: 96–101.

[49] Nomura H, Ogawa A, Tashiro A, Morimoto T, Hu JW, Iwata K. Induction of Fos protein-like immunoreactivity in the trigeminal spinal nucleus caudalis and upper cervical cord following noxious and non-noxious mechanical stimulation of the whisker pad of the rat with an inferior alveolar nerve transection. Pain. 2002; 95: 225–238.

[50] Ogawa A, Ren K, Tsuboi Y, Morimoto T, Sato T, Iwata K. A new model of experimental parotitis in rats and its implication for trigeminal nociception. Experimental Brain Research. 2003; 152: 307–316.

[51] Ogawa A, Meng ID, Ren K, Imamura Y, Iwata K. Differential responses of rostral subnucleus caudalis and upper cervical dorsal horn neurons to mechanical and chemical stimulation of the parotid gland in rats. Brain Research. 2006; 1106: 123–133.

[52] Okamoto K, Imbe H, Kimura A, Donishi T, Tamai Y, Senba E. Activation of central 5HT2A receptors reduces the craniofacial nociception of rats. Neuroscience. 2007; 147: 1090–1102.

[53] Okamoto K, Bereiter DF, Thompson R, Tashiro A, Bereiter DA. Estradiol replacement modifies c-fos expression at the spinomedullary junction evoked by temporomandibular joint stimulation in ovariectomized female rats. Neuroscience. 2008; 156: 729–736.

[54] Okamoto K, Kimura A, Donishi T, Imbe H, Senba E, Tamai Y. Central serotonin 3 receptors play an important role in the modulation of nociceptive neural activity of trigeminal subnucleus caudalis and nocifensive orofacial behavior in rats with persistent temporomandibular joint inflammation. Neuroscience. 2005; 135: 569–581.

[55] Panneton WM. Primary afferent projections from the upper respiratory tract in the muskrat. Journal of Comparative Neurology. 1991; 308: 51–65.

[56] Park J, Trinh VN, Sears-Kraxberger I, Li KW, Steward O, Luo ZD. Synaptic ultrastructure changes in trigeminocervical complex posttrigeminal nerve injury. Journal of Comparative Neurology. 2016; 524: 309–322.

[57] Puri J, Bellinger LL, Kramer PR. Estrogen in cycling rats alters gene expression in the temporomandibular joint, trigeminal ganglia and trigeminal subnucleus caudalis/upper cervical cord junction. Journal of Cellular Physiology. 2011; 226: 3169–3180.

[58] Qu ZY, Liu L, Zhao LP, Xu XB, Li ZJ, Zhu YP, *et al*. Prophylactic electroacupuncture on the upper cervical segments decreases neuronal discharges of the trigeminocervical complex in migraine-affected rats: an *in vivo* extracellular electrophysiological experiment. Journal of Pain Research. 2020; 13: 25–37.

[59] Sabino MA, Honore P, Rogers SD, Mach DB, Luger NM, Mantyh PW. Tooth extraction-induced internalization of the substance P receptor in trigeminal nucleus and spinal cord neurons: imaging the neurochemistry of dental pain. Pain. 2002; 95: 175–186.

[60] Sato T, Kitagawa J, Ren K, Tanaka H, Tanabe A, Watanabe T, *et al*. Activation of trigeminal intranuclear pathway in rats with temporomandibular joint inflammation. Journal of Oral Science. 2005; 47: 65–69.

[61] Sessle BJ, Hu JW, Amano N, Zhong G. Convergence of cutaneous, tooth pulp, visceral, neck and muscle afferents onto nociceptive and non-nociceptive neurones in trigeminal subnucleus caudalis (medullary dorsal horn) and its implications for referred pain. Pain. 1986; 27: 219–235.

[62] Shibuta K, Suzuki I, Shinoda M, Tsuboi Y, Honda K, Shimizu N, *et al*. Organization of hyperactive microglial cells in trigeminal spinal subnucleus caudalis and upper cervical spinal cord associated with orofacial neuropathic pain. Brain Research. 2012; 1451: 74–86.

[63] Shigenaga Y, Sera M, Nishimori T, Suemune S, Nishimura M, Yoshida A, *et al*. The central projection of masticatory afferent fibers to the trigeminal sensory nuclear complex and upper cervical spinal cord. Journal of Comparative Neurology. 1988; 268: 489–507.

[64] Shigenaga Y, Chen IC, Suemune S, Nishimori T, Nasution ID, Yoshida A, *et al*. Oral and facial representation within the medullary and upper cervical dorsal horns in the cat. Journal of Comparative Neurology. 1986; 243: 388–408.

[65] Shigenaga Y, Okamoto T, Nishimori T, Suemune S, Nasution I, Chen I, *et al*. Oral and facial representation in the trigeminal principal and rostral spinal nuclei of the cat. Journal of Comparative Neurology. 1986; 244: 1–18.

[66] Shimizu K, Asano M, Kitagawa J, Ogiso B, Ren K, Oki H, *et al*. Phosphorylation of extracellular signal-regulated kinase in medullary and upper cervical cord neurons following noxious tooth pulp stimulation. Brain Research. 2006; 1072: 99–109.

[67] Strassman AM, Vos BP. Somatotopic and laminar organization of fos-like immunoreactivity in the medullary and upper cervical dorsal horn induced by noxious facial stimulation in the rat. Journal of Comparative Neurology. 1993; 331: 495–516.

[68] Strassman AM, Mineta Y, Vos BP. Distribution of fos-like immunoreactivity in the medullary and upper cervical dorsal horn produced by stimulation of dural blood vessels in the rat. Journal of Neuroscience. 1994; 14: 3725–3735.

[69] Sugimoto T, Hara T, Shirai H, Abe T, Ichikawa H, Sato T. c-fos induction in the subnucleus caudalis following noxious mechanical stimulation of the oral mucous membrane. Experimental Neurology. 1994; 129: 251–256.

[70] Sugimoto T, Fujiyoshi Y, Xiao C, He YF, Ichikawa H. Central projection of calcitonin gene-related peptide (CGRP)- and substance P (SP)-immunoreactive trigeminal primary neurons in the rat. The Journal of Comparative Neurology. 1997; 378: 425–442.

[71] Suzuki I, Kitagawa J, Noma N, Tsuboi Y, Kondo M, Honda K, *et al*. Attenuation of naloxone-induced Vc pERK hyper-expression following capsaicin stimulation of the face in aged rat. Neuroscience Letters. 2008; 442: 39–43.

[72] Takemura M, Sugimoto T, Sakai A. Topographic organization of central terminal region of different sensory branches of the rat mandibular nerve. Experimental Neurology. 1987; 96: 540–557.

[73] Takeshita S, Hirata H, Bereiter DA. Intensity coding by TMJ-responsive neurons in superficial laminae of caudal medullary dorsal horn of the rat. Journal of Neurophysiology. 2001; 86: 2393–2404.

[74] Tanimoto T, Takeda M, Nishikawa T, Matsumoto S. The role of 5-hydroxytryptamine_3_ receptors in the vagal afferent activation-induced inhibition of the first cervical dorsal horn spinal neurons projected from tooth pulp in the rat. Journal of Pharmacology and Experimental Therapeutics. 2004; 311: 803–810.

[75] Tanimoto T, Takeda M, Matsumoto S. Suppressive effect of vagal afferents on cervical dorsal horn neurons responding to tooth pulp electrical stimulation in the rat. Experimental Brain Research. 2002; 145: 468–479.

[76] Tashiro A, Okamoto K, Bereiter DA. Chronic inflammation and estradiol interact through MAPK activation to affect TMJ nociceptive processing by trigeminal caudalis neurons. Neuroscience. 2009; 164: 1813–1820.

[77] Yasuda K, Furusawa K, Tanaka M, Yamaoka M. The distribution of afferent neurons in the trigeminal mesencephalic nucleus and the central projection of afferent fibers of the mylohyoid nerve in the rat. Somatosensory & Motor Research. 1995; 12: 309–315.

[78] Vos BP, Strassman AM. Fos expression in the medullary dorsal horn of the rat after chronic constriction injury to the infraorbital nerve. The Journal of Comparative Neurology. 1995; 357: 362–375.

[79] Young RF, Perryman KM. Pathways for orofacial pain sensation in the trigeminal brain-stem nuclear complex of the Macaque monkey. Journal of Neurosurgery. 1984; 61: 563–568.

[80] Zerari-Mailly F, Dauvergne C, Buisseret P, Buisseret-Delmas C. Localization of trigeminal, spinal, and reticular neurons involved in the rat blink reflex. Journal of Comparative Neurology. 2003; 467: 173–184.

[81] Zhou Q, Imbe H, Dubner R, Ren K. Persistent Fos protein expression after orofacial deep or cutaneous tissue inflammation in rats: Implications for persistent orofacial pain. Journal of Comparative Neurology. 1999; 412: 276–291.

[82] Westberg KG, Olsson KA. Integration in trigeminal premotor interneurones in the cat. 1. Functional characteristics of neurones in the subnucleus-gamma of the oral nucleus of the spinal trigeminal tract. Experimental Brain Research. 1991; 84: 102–114.

[83] Kubo A, Sugawara S, Iwata K, Yamaguchi S, Mizumura K. Masseter muscle contraction and cervical muscle sensitization by nerve growth factor cause mechanical hyperalgesia in masticatory muscle with activation of the trigemino‐lateral parabrachial nucleus system in female rats. Headache. 2022; 62: 1365–1375.
