# Supplementary material for: Integration of nociceptive activity from orofacial, cranial and cervical regions in the trigeminocervical nucleus: a scoping review with clinical implications
Source: J Oral Facial Pain Headache. 2025 Sep 12;39(3):1–12. doi: 10.22514/jofph.2025.042 (PMC12531578; doi:10.22514/jofph.2025.042)
Supplement: Supplementary file 4 [file Supplementary-material-4.docx]

Supplementary material

Supplementary material 4. Detailed description of included studies regarding the connection between the cranial, orofacial, and cervical regions through the TCN.

|  | Cervical to orofacial | Stimulus Cervical | Area of Response Orofacial | TNC Area  Vc/Vi | Obex | Vc/C1 | Others | Orofacial to cervical | Stimulus Orofacial | Area of Response: Cervical or other areas | TNC Area  Vc/Vi | Obex | Vc/C1 | Written conclusion supporting the direction of the connection |
| --- | --- | --- | --- | --- | --- | --- | --- | --- | --- | --- | --- | --- | --- | --- |
| STIMULUS OROFACIAL (OROFACIAL/CRANIAL TO CERVICAL DIRECTION) | | | | | | | | | | | | | | |
| Bereiter, 2002 [1] | 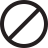 | NA | NA | 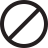 | 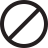 | 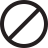 | 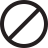 | 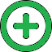 | TMJ | Cervical: Upper cervical spinal cord  Other areas: Caudal brainstem | 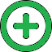 | 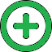 | 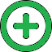 | Acute injury to the TMJ region caused a similar pattern of Fos-LI in the caudal brainstem and upper cervical spinal cord in males and females. Sex steroids play a role in mediating the interaction between vagal afferent activity and opioid analgesia in deep craniofacial pain conditions such as TMD. |
| Bereiter, 2001 [2] | 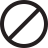 | NA | NA | 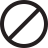 | 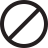 | 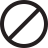 | 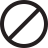 | 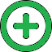 | TMJ | Cervical: Upper cervical dorsal horn ipsilateral  Other areas: Trigeminal  spinal nucleus | 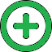 | 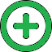 | 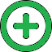 | Vc/C2 junction  region plays a critical role in the integration of pain signals originating from the TMJ region. |
| Casatii, 1999 [3] | 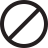 | NA | NA | 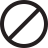 | 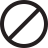 | 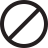 | 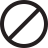 | 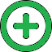 | TMJ | Cervical: Trigeminal, dorsal root (C2 to C6), superior cervical | 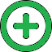 | 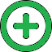 | 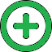 | The sympathetic innervation is supplied by the superior cervical and stellate ganglia and the parasympathetic innervation by the otic ganglion. |
| Du, 2017 [4] | 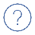 | NA | NA | 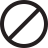 | 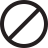 | 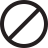 | 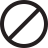 | 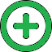 | TMJ | Cervical: Splenius and trapezius muscles | 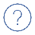 | 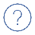 | 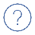 | The TMJ receptors may be involved in the mechanical theory of the head and neck muscle's nervous system regulation. |
| Hathaway, 1995 [5] | 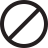 | NA | NA | 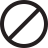 | 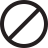 | 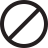 | 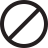 | 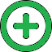 | TMJ | Cervical: Upper cervical spinal cord (C1 to C3)  Other areas: Brainstem (Vo; Vi; Vi/Vc; Vp; Vc) | 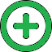 | 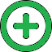 | 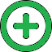 | The wide rostro caudal distribution of Fos-LI within the caudal brainstem reflects the distribution of TMJ-responsive nociceptive neurons that may underlie the spread and referral of pain from the TMJ region. |
| Kramer, 2013 [6] | 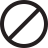 | NA | NA | 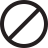 | 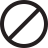 | 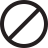 | 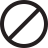 | 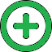 | TG (TMJ) | Cervical: Upper cervical region  Other areas: TNC caudalis | 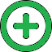 | 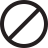 | 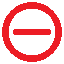 | GABAA receptors consisting of the Gabrα6 subunit inhibit TG nociceptive sensory afferents in the trigeminal pathway and have an important role in the regulation of myofascial nociception. |
| Imbe and Ren, 1999 [7] | 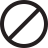 | NA | NA | 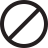 | 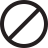 | 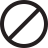 | 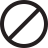 | 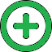 | TMJ | Cervical: C1 to C3  Other areas: Vi/Vc transition; Vi ; Vc | 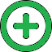 | 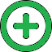 | 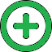 | These changes may contribute to persistent central hyperexcitability and pain associated with TMD. |
| Lam, 2009 [8] | 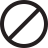 | NA | NA | 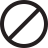 | 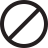 | 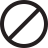 | 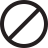 | 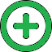 | TMJ | Cervical: Upper cervical cord (Vc/UCC)  Other areas: Trigeminal subnucleus caudalis | 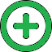 | 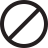 | 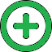 | Peripheral and central sensitization may be differentially involved in the nociceptive effects of glutamate and capsaicin applied to deep craniofacial tissues. |
| Lam, 2008 [9] | 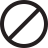 | NA | NA | 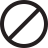 | 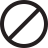 | 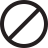 | 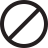 | 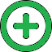 | TMJ | Cervical: Upper cervical cord  Other areas: Trigeminal subnucleus caudalis | 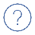 | 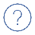 | 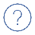 | Central sensitization induced by capsaicin alone or by cutaneous incision alone can readily occur in TMJ-responsive nociceptive neurons and that following incision-induced excitability increases. |
| Okamoto, 2008 [10] | 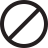 | NA | NA | 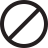 | 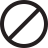 | 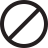 | 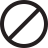 | 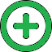 | TMJ | Cervical: Vc/C1-2 junction  Other areas: Trigeminal subnucleus caudalis (Vc); Vi/Vcvl | 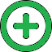 | 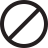 | 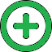 | These results suggest that the stimulus on the TMJ nociceptive area influences the nociceptive processing at the Vc/C1-2 junction. |
| Okamoto, 2005 [11] | 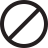 | NA | NA | 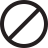 | 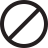 | 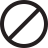 | 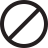 | 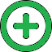 | TMJ | Cervical: Upper cervical spinal cord junction  Other areas: Trigeminal subnucleus caudalis | 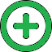 | 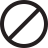 | 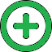 | The stimulus on the orofacial area (TMJ) modifies neural activity at the level of the Vc/C2 junction region relevant for TMD pain. |
| Puri, 2011 [12] | 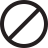 | NA | NA |  |  |  |  |  | TMJ | Cervical: Upper cervical cord junction (Vc/C1–2)  Other areas: Trigeminal subnucleus caudalis |  |  |  | There is one positive influence of the TMJ stimulus on the neuronal excitability identified by changes in the concentration of 17β-estradiol at the upper cervical area. |
| Zhou, 1999 [13] |  | NA | NA |  |  |  |  |  | TMJ | Cervical: Upper cervical cord nearly coplanar (laminae I/II, II/IV, and V/VI)  Other areas: The caudal medulla |  |  |  | The stimulus at the jaw induces a distribution of Fos-positive cells were mainly distributed in laminae I/II and V/VI; few Fos-labeled neurons were found in laminae III/IV. |
| Takeshita, 2001 [14] |  | NA | NA |  |  |  |  |  | TMJ | Cervical: Upper cervical (C1–C2) spinal cord  Other areas: Subnucleus caudalis |  |  |  | Just the mechanical stimulus at the orofacial region can influence the proportion of labeling at the Vc and C1–2 levels. |
| Marfurt and Rajchert, 1991 [15] |  | NA | NA |  |  |  |  |  | Mandibular region | Cervical: Cervical area  Other areas: Laminae areas |  |  |  | Somatosensory information from the head and face may be transmitted directly to widespread and functionally heterogeneous areas of the rat central nervous system. |
| Chibuzo, 1981 [16] |  | NA | NA |  |  |  |  |  | Tongue, intermandibular | Cervical: First cervical ganglia  Other areas: Trigeminal ganglia |  |  |  | Large numbers of sensory neurons were concentrated ventromedially within the mandibular zone of the trigeminal ganglion. |
| Adachi, 2010 [17] |  | NA | NA |  |  |  |  |  | Molar tooth pulp | Cervical: Vc, Vi/C1 C1/2  Other areas: TMJ muscles |  |  |  | The activation of receptors in the tooth pulp is sufficient to elicit nociceptive behavioral responses and trigeminal brainstem neuronal activity, including the first cervical levels (C1/C2). |
| Tanimoto, 2002 [18] |  | NA | NA |  |  |  |  |  | Pulp of upper incisors | Cervical: C1 spinal neuronal activity |  |  |  | These results suggest that vagal afferent stimulation inhibits nociceptive transmission in the C1 spinal neuron activity via the activation of both noradrenergic and serotonergic descending inhibitory systems, from the stimulus of the pulp of upper incisors. |
| Tanimoto, 2004 [19] |  | NA | NA |  |  |  |  |  | Pulp of upper incisors | Cervical: C1 spinal neuronal activity |  |  |  | These results suggest that vagal afferent stimulation-induced suppression of C1 spinal neuron activity, responding to tooth pulp stimulation, ion-channel activation. |
| Marfurt, 1984 [20] |  | NA | NA |  |  |  |  |  | First maxillary pulp chamber | Cervical: spinal cord segments C1 and C2  Other areas: Brainstem, cerebellum, trigeminal ganglia |  |  |  | The maxillary tooth pulp afferent fibers terminate in a markedly dorsal area of the TBNC. |
| Sabino, 2002 [21] |  | NA | NA |  |  |  |  |  | Maxillary incisor or molar | Cervical: Spinal cord  Other areas: Trigeminal complex |  |  |  | The stimulus on the tooth has increased the activity in the spinal cord area. |
| Shimizu, 2006 [22] |  | NA | NA |  |  |  |  |  | Upper or lower dental tooth pulp | Cervical: Upper cervical cord neurons  Other areas: Medullary neurons |  |  |  | The tooth-pulp-driven neurons in the spinal trigeminal nucleus are involved in tooth-pulp pain through activation of the intracellular signal transduction pathway. |
| Matsumoto, 1999 [23] |  | NA | NA |  |  |  |  |  | Tooth pulp and phrenic nerve fibers | Cervical: C1 spinal neurons |  |  |  | There may be the convergence of face, neck, jaw, TP, and PN afferents on the same C1 spinal neurons In the rat. |
| Kato, 2003 [24] |  | NA | NA |  |  |  |  |  | Nostril | Cervical: Cervical muscles  Other areas: Brainstem |  |  |  | If this new variant of the trigeminal cervical reflex exists in humans, then it may be useful as a clinical tool for testing the integrity of the brainstem and C1 and C2 since the circuit is now identified and characterized in the animal model. |
| Busch, 2006 [25] |  | NA | NA |  |  |  |  |  | Forehead | Cervical: C1–C3 |  |  |  | The anatomical and functional convergence of trigeminal and cervical afferent pathways in animals and the modulation of this pathway is of potential benefit in primary headache disorders. |
| Chudler, 1991 [26] |  | NA | NA |  |  |  |  |  | Head, face | Cervical: C1  Other areas: Medullary dorsal horn |  |  |  | Some neurons in the first cervical segment of the spinal cord receive convergent input for trigeminal and cervical pathways and may be involved in mediating orofacial and cranial pain. |
| Classey, 2001 [27] |  | NA | NA |  |  |  |  |  | Head (sagittal) | Cervical: C1/2/3 levels of upper cervical cord  Other areas: TNC; caudal medulla |  |  |  | Fos protein expression was reduced in the cervical (upper spinal cord) region following the administration of MK-801, suggesting the involvement of glutamate in neurotransmission within the trigeminocervical complex. This suggests a potential link from the orofacial (trigeminal) region to the cervical region in the context of trigeminovascular activation. |
| Noma, 2017 [28] |  | NA | NA |  |  |  |  |  | Whisker pad | Cervical: Upper cervical spinal cord (C1–C2)  Other areas: Trigeminal spinal subnucleus caudalis (Vc) |  |  |  | By suppressing Vc neuronal activity, high-dose intradermal injection of BoNT-A at the site of ION innervation alleviates mechanical facial allodynia and hyperalgesia associated with ION-CCI. |
| Nomuera, 2002 [29] |  | NA | NA |  |  |  |  |  | Whisker pad | Cervical: First segment of the spinal cord (C1)  Other areas: Trigeminal spinal nucleus caudalis (Vc) |  |  |  | The high numbers and spatial arrangement of nociceptive neurons in the Vc and C1 after IAN transection reflect the influence of the orofacial area in the cervical area. |
| Suziki, 2008 [30] |  | NA | NA |  |  |  |  |  | Whisker pad | Cervical: Upper cervical spinal cord neurons  Other areas: Trigeminal spinal subnucleus caudalis (Vc) |  |  |  | The descending modulation system is impaired with advancing age, resulting in abnormal pain sensation in aged rats. |
| Kamimura, 2018 [31] |  | NA | NA |  |  |  |  |  | Whisker-pad area | Vc or C1–C2 tissue |  |  |  | Inhibition of the degradation of the major endocannabinoid 2-AG is a promising therapeutic option for orofacial neuropathic pain. |
| Honda, 2011 [32] |  | NA | NA |  |  |  |  |  | Tongue or whisker pad skin | Cervical: C1–C2 neurons  Other areas: Vc |  |  |  | The peripheral Glu receptor mechanisms may contribute to cold hyperalgesia in the tongue but not in the facial skin, and also contribute to heat hyperalgesia in the tongue and facial. |
| Hu, 1992 [33] |  | NA | NA |  |  |  |  |  | Masseter muscle | Cervical: spinal tract nucleus  Other areas: Subnucleus caudalis/Vo |  |  |  | Mustard oil injection into the deep masseter muscle increased the excitability of trigeminal nociceptive neurons, indicating a link between deep craniofacial afferents and pain mechanisms. |
| Kramer, 2014 [34] |  | NA | NA |  |  |  |  |  | Masseter muscle | Cervical: C1–2  Other areas: V3, Vc |  |  |  | The estradiol decreased the orofacial nociceptive response, in part, by causing an increase in Gabrα6 expression. |
| Kubo, 2022 [35] |  | NA | NA |  |  |  |  |  | Masseter muscle | Other areas: Lower brainstem |  |  |  | The number of pERK-immunoreactive neurons in the brainstem was increased significantly after the Trapezius and masseter muscles stimulation. |
| Kurose, 2017 [36] |  |  |  |  |  |  |  |  | masseter muscle (MM) | Cervical: Vc/C2 region  Other Area: Medula, brainstem |  |  |  | A persistent TMJ inflammation for 10 and 14 days is sufficient to enhance MM nociception indicated by behaviors and neural responses in superficial laminae at the Vc/C2 region. |
| Okamoto, 2007 [37] |  | NA | NA |  |  |  |  |  | Left masseter muscle | Cervical: Superficial laminae at the Vc/C2 region  Other areas: Trigeminal subnucleus caudalis (Vc) |  |  |  | The stimulus on the orofacial nociceptive area through serotonin receptors can reduce nociceptive neural activities in the Vc/C2 region. |
| Luo, 1995 [38] |  | NA | NA |  |  |  |  |  | Diastema of the mandible | Cervical: Cervical spinal cord  Other areas:  Mesencephalic nucleus of the trigeminal nerve |  |  |  | This projection of jaw-muscle spindle afferents to caudal brainstem regions may play a significant role in masticatory-muscle stretch reflexes. |
| Nishimo, 1986 [39] |  | NA | NA |  |  |  |  |  | Masseter nerve, tooth pulp | Cervical: Upper cervical dorsal horn  Other areas: Nucleus interpolaris; nucleus caudalis |  |  |  | Muscle pain from the masseter muscle is relayed to the second-order neurons in the caudal parts of the pars interpolaris and laminae I/V, and subsequently mediated to the sensory cortex via VPM and posterior nucleus (POre). |
| Shigena, 1986 [40] |  | NA | NA |  |  |  |  |  | Masseter, temporal, pterygoid, digestric, myolohoid | Cervical: Cervical cord  Other areas: Brainstem, and trigeminal ganglia |  |  |  | After a cold stimulus of the orofacial nerve, an improvement in the afferent cells of the cervical area was identified. |
| Chiaia, 1987 [41] |  | NA | NA |  |  |  |  |  | Orofacial region  (guard-hair) | Cervical: The rostral portion of the first cervical segment  Other areas: Subnucleus interpolaris, and the medullary dorsal horn (Obex) |  |  |  | Fibers with rostral receptive fields tended to terminate in the rostral portion of the MDH while primary afferents with caudal fields gave off most of their collaterals in the caudal MDH and the rostral portion of the first cervical segment. |
| Demartini, 2017 [42] |  | NA | NA |  |  |  |  |  | Right lip | Cervical: Cervical spinal cord (CSS)  Other areas: Medulla, trigeminal ganglion |  |  |  | There is an involvement of TRPA1 channels in the pathophysiology of migraine, and show their active role in counteracting hyperalgesia at the trigeminal level. |
| Goadsby, 1991 [43] |  | NA | NA |  |  |  |  |  | Superior sagittal sinus | Cervical: Upper cervical cord  Other areas: Brainstem |  |  |  | The dorsolateral area activated in the spinal cord corresponds to a group of cells in or near the lateral cervical nucleus that may form an important relay for craniovascular nociception and thus be of relevance to the mechanism of headache. |
| Kaube, 1993 [44] |  | NA | NA |  |  |  |  |  | Sagittal sinus | Cervical: C1–C3 level |  |  |  | Several populations of neurons likely to be involved in the central neural processing of vascular headache syndromes, particularly migraine. |
| Honda, 2008 [45] |  | NA | NA |  |  |  |  |  | Facial skin | Cervical: C1–C2 neurons  Other areas: Vc |  |  |  | The capsaicin treatment of the lateral facial skin causes an enhancement of ERK phosphorylation in Vc and C1–C2 neurons as well as induces nocifensive behavior to heat, cold and mechanical simulation of the capsaicin-treated skin. |
| Hu, 2005 [46] |  | NA | NA |  |  |  |  |  | Deep craniofacial tissues | Cervical: C1 and C2  Other areas: Rostral subnucleus caudalis (rVc) |  |  |  | Nociceptive neurons in C1 and C2 regions had receptive fields covering craniofacial and cervical tissues, including deep craniofacial areas. This suggests an integration of nociceptive information from these regions in C1 and C2 DHs. |
| Jacquin, 1986 [47] |  | NA | NA |  |  |  |  |  | V ganglion; vibrissa, intermediate length hair, hairy skin, guard hair, nociceptive, nasal mucosal afferent | Cervical: C1  Other areas: Medullary dorsal horn (MDH) |  |  |  | The “onion-leaf” rostrocaudal organization  of V afferents in the- MDH and cervical dorsal horn, as well as an “inside-out”  vibrissae map in those mid- and cat&l-MDH regions where overlap of terminals from axons innervating different follicles within a given row occur. |
| Jacquin, 1983 [48] |  | NA | NA |  |  |  |  |  | IO nerve section and vibrissae follicle | Cervical: C1; C2  Other areas: Vprinc; Vo; Vi; Vc |  |  |  | Transganglionic HRP transport from the “regenerate” IO nerve indicated an almost exclusive projection to the marginal layer of the medullary and rostral cervical dorsal horn. |
| Luz, 2019 [49] |  | NA | NA |  |  |  |  |  | Trigeminal nerve together with its ophthalmic branch | Cervical: C1 to C4 |  |  |  | The trigeminocervical lamina I neurons receive a complex pattern of long-range monosynaptic and polysynaptic inputs from a variety of trigeminal nociceptive afferents. |
| Lyubashina, 2012 [50] |  | NA | NA |  |  |  |  |  | Left vagal nerve and facial cutaneous receptive field | Cervical: Left STN at the level of C1 spinal cord |  |  |  | These results provide evidence of VNS-induced modulation of  trigeminovascular nociception, and therefore contribute to a deeper understanding of neurophysiological mechanisms  underlying effects of vagal stimulation in chronic  headaches. |
| Lyubashina, 2017 [51] |  | NA | NA |  |  |  | C1 level |  | Dura mater close to the superior sagittal sinus; distal part of the GON | Spinal cord at the c1 level |  |  |  | Electrical stimulation of the greater occipital nerve produces suppression of both the ongoing activity of the convergent spinal trigeminal neurons and their responses to electrical stimulation of the dura mater. |
| Margolis, 1989 [52] |  | NA | NA |  |  |  |  |  | Chamber of the left eye | Other areas: Brain stem |  |  |  | The principal route of spread of infection in the CNS is transneuronal, presumably via axonal transport. |
| Morch, 2007 [53] |  | NA | NA |  |  |  | NA |  | Cornea, dura, hypoglossal nerve, TMJ, masseter muscle and superior laryngeal nerve, tongue | Cervical: First cervical dorsal horn |  |  |  | The afferent convergence in first cervical dorsal horn nociceptive neurons may be limited to the craniofacial area and that they may play an important role in the integration of craniofacial and upper cervical nociceptive inputs. |
| Nakajima, 2011 [54] |  | NA | NA |  |  |  |  |  | Infraorbital nerve | Cervical: upper cervical spinal cord (C1/C2)  Other areas: Vc |  |  |  | PKCγ expression in the Vc played an important role in the mechanism of orofacial static mechanical allodynia following trigeminal nerve injury. |
| Noma, 2008 [55] |  | NA | NA |  |  |  |  |  | Infraorbital nerve | Cervical: upper cervical spinal cord (C1–C2)  Other areas: Trigeminal spinal subnucleus caudalis (Vc) |  |  |  | The capsaicin-sensitive small-fiber afferent inputs acutely activated by stimulation of facial and intraoral sites can produce ERK phosphorylation in the Vi/Vc zone, middle Vc, and Vc/C2 zone. |
| Park, 2016 [56] |  | NA | NA |  |  |  |  |  | Infraorbital nerve | Cervical: C2  Other areas: Trigeminal spinal subnucleus caudalis (Vc) |  |  |  | After the orofacial stimulus an increase in the number of excitatory synapses in the superficial dorsal horn of Vc/C2 was identified. |
| Panneton, 1991 [57] |  | NA | NA |  |  |  |  |  | Ethmoidal nerve | Cervical: Cervical spinal cord |  |  |  | There is a convergence of primary afferent fibers innervating the upper respiratory tract of the muskrat into the paratrigeminal nuclei and lamina I of the medullary dorsal horn. |
| Ogawa, 2003 [58] |  | NA | NA |  |  |  |  |  | Parotid gland | Cervical: upper cervical (C2) dorsal horn  Other areas: Trigeminal spinal nucleus interpolaris (Vi) and caudalis (Vc) and paratrigeminal nucleus (Pa5) |  |  |  | A large number of Fos protein-LI cells were expressed bilaterally in the ventral portion of the Vi/Vc and the C2 after the stimulus of parotid gland. |
| Ogawa, 2006 [59] |  | NA | NA |  |  |  |  |  | Parotid gland | Cervical: C1/2 spinal cord  Other areas: Medulla |  |  |  | The stimulus of the parotid gland influences the sensitization of C1/C2, but not Vi/Vc nociceptive neurons. |
| Sato, 2005 [60] |  | NA | NA |  |  |  |  |  | Posterior edge of the zygomatic arc | Cervical: Vc/C1,2  Other areas: Vi/Vc |  |  |  | The TMJ inflammation is specifically relayed to the Vi/Vc transition zone via the caudal laminated Vc/C1-2 zone. |
| Shibuta, 2012 [61] |  | NA | NA |  |  |  |  |  | Infraorbital nerve | Cervical: Upper cervical spinal cord (C1)  Other areas: Trigeminal spinal subnucleus caudalis (Vc) |  |  |  | Glutamate application to the tongue or whisker pad skin caused an enhancement of head-withdrawal reflex and ERK phosphorylation in Vc and C1–C2 neurons to heat stimulation. |
| Strassman, 1994 [62] |  | NA | NA |  |  |  |  |  | Sagittal sinus | Cervical: Upper cervical  Other areas: Medullary dorsal horn; TNC |  |  |  | The stimulus in the orofacial area, improve the input at the rostrocaudal levels, in C1 and the interpolaris-caudalis transition region. |
| Strassmand, 1993 [63] |  | NA | NA |  |  |  |  |  | Mandibulary, maxillary, ophthalmic tissues; infraorbital | Cervical: C1, C2  Other areas: Medullary dorsal horn (nucleus caudalis) and nucleus interpolaris |  |  |  | The interpolaris-caudalis transition region may have properties that are distinct from those of the rest of the trigeminal complex, possibly related to an involvement in autonomic function. |
| Sugimoto, 1994 [64] |  | NA | NA |  |  |  |  |  | Maxillary first molar, tongue, upper and lower eyelid, vibrissal pad | Cervical: Spinal cord  Other areas: Subnucleus caudalis |  |  |  | The stimulus in the orofacial area results in a clear somatotopic segregation in laminae I and II of the first and second cervical segments of the spinal cord ipsilateral to the stimulation. |
| Takemura, 1987 [65] |  | NA | NA |  |  |  |  |  | Left auriculotemporal, mylohyoid, inferior alveolar, mental, lingual and buccal nerves | Cervical: Dorsal horn from medulla to the C3  Other areas: Nucleus principalis, subnuclei oralis and interpolaris, |  |  |  | The contralateral projection was reported at the cervical segment after a chemical stimulus at the alveolar nerve on TMJ. |
| Sessle, 1986 [66] |  | Cervical skin and muscle | NA |  |  |  |  |  | Facial skin, oral mucosa, canine and premolar tooth pulp,  laryngeal mucosa, jaw,tongue muscles | Other areas: trigeminal (V) subnucleus caudalis (medullary dorsal horn) at levels O-3 mm caudal to the obex. |  |  |  | The stimulus on the orofacial area demonstrated an improvement of the neuronal activity at the cervical area of the TCN. |
| Sugimoto, 1997 [67] |  | NA | NA |  |  |  |  |  | Trigeminal nerve | Lower brainstem |  |  |  | The glossopharyngeal and vagal primaries are candidates for the source of CGRP-ir projection to the Vo and the MDH, while the dorsal root axons supply the MDH with CGRP-ir terminals. Contralateral primary neurons crossing the midline appear to contain CGRP and to terminate in the MDH. |
| Westberg, 1991 [68] |  | Cervical nerves | NA |  |  |  |  |  | Trigeminal, facial | Other areas: trigeminal tract; different trigeminal sensory nuclei |  |  |  | The stimulus applied at the trigeminal tract trigger inputs at the orofacial region. |
| Shigenaga, Chen *et al*. [69] 1986 |  | NA | NA |  |  |  |  |  | Facial skin | Cervical Spine: C1-C2 nociceptive neurons  Other areas: Vc |  |  |  | An improvement on the afferents of the cervical area were identified after a stimulus of the orofacial nerve. |
| Shigenaga, Sera *et al*. 1988 [70] |  | NA | NA |  |  |  |  |  | Jaw-closing muscles | Cervical Spine: Cervical cord  Other areas: Brainstem and trigeminal ganglia |  |  |  | After a stimulus of the orofacial nerve, an improvement on the afferents of the cervical area were identified. |
| Tashiro, Okamoto *et al*. [71] 2009 |  | NA | NA |  |  |  |  |  | TMJ | Cervical Spine: C1-2; Spinomedullary junction  Other areas: Vc |  |  |  | Multiple regions of the caudal trigeminal brainstem complex integrate light-related sensory information. |
| Yasuda, Furusawa *et al*. [72] 1995 |  | NA | NA |  |  |  |  |  | Mylohoid muscle, digatricus muscle and cutaneous branch | Cervical Spine: Upper cervical  Other areas: Ipsilateral trigeminal nucleus caudalis |  |  |  | The mylohoid and digastricus afferent muscle fibers are in lamina I in the Vc and the upper cervical dorsal horn. |
| Vos and Strassman 1995 [73] |  | NA | NA |  |  |  |  |  | Hair | Cervical Spine: Upper cervical cord  Other areas: Laminae I-II and laminae III-IV |  |  |  | The stimulus in the hair does not affect the number of Fos-Li cells at the cervical area. |
| Young and Perryman 1984 [74] |  | NA | NA |  |  |  |  |  | Dental pulp and facial skin | Cervical Spine: C-l-C-3  Other areas: nucleus caudalis, nucleus principalis, nucleus oralis, nucleus interpolaris |  |  |  | Primary afferent fibers for cutaneous facial nociception are contained in the trigeminal, facial, glossopharyngeal, and vagus nerves, and the upper cervical dorsal and ventral roots. |
| STIMULUS CERVICAL (CERVICAL TO OROFACIAL/CRANIAL DIRECTION) | | | | | | | | | | | | | | |
| First author, year | Cervical to orofacial | Stimulus Cervical | Area of Response Orofacial | TNC Area  Vc/Vi | Obex | Vc/C1 | Others | Orofacial to cervical | Stimulus Orofacial | Area of Response: Cervical or other areas | TNC Area  Vc/Vi | Obex | Vc/C1 | Written conclusion supporting the direction of the connection |
| Kiyomoto, 2013 [75] |  | Trapezius muscles | Facial Skin |  |  |  |  |  | NA | NA |  |  |  | FKN signaling may be a promising therapeutic target for treating orofacial pain associated with back and neck pain. |
| Kobayashi, 2011 [76] |  | Upper cervical spinal nerve transection (CNX) | lateral facial skin |  |  |  | C1-C2 |  | NA | NA |  |  |  | Mechanical allodynia and thermal hyperalgesia occur in the lateral facial skin after CNX. |
| Kiyomoto, 2015 [77] |  | Trapezius muscles | Facial skin |  |  |  |  |  |  |  |  |  |  | There is a mechanical hypersensitivity in the lateral facial skian associated with trapezius muscle inflammation. |
| Miyamoto, 2011 [78] |  | Whisker pad skin ipsilateral | Vc and C1–C2 |  |  |  | C1-C2 |  | NA | NA |  |  |  | GluR2 and GluR3 subunits of AMPA receptor play roles in the trigeminal nerve injury-mediated enhancement of Vc and C1–C2 neuronal excitability, and  hyperalgesia. |
| Qu, 2020 [79] |  | Acupoint GB20 (upper cervical tissues) | Face |  |  |  |  |  | NA | NA |  |  |  | There is one reduction of the discharge frequency of neurons in the TCC after stimulus. |
| BOTH DIRECTIONS INVESTIGATED (OROFACIAL/CRANIAL TO CERVICAL AND CERVICAL TO OROFACIAL/CRANIAL) | | | | | | | | | | | | | | |
| First author, year | Cervical to orofacial | Stimulus Cervical | Area of Response Orofacial | TNC Area  Vc/Vi | Obex | Vc/C1 | Others | Orofacial to cervical | Stimulus Orofacial | Area of Response: Cervical or other areas | TNC Area  Vc/Vi | Obex | Vc/C1 | Written conclusion supporting the direction of the connection |
| Kamimura, 2018 [31] |  | C1 | Face |  |  |  |  |  | Infraorbital nerve | Cervical: C1, C2  Other areas: Vc |  |  |  | Inhibition of 2-AG degradation by MAGL inhibi- tors is a promising therapeutic option for treatment of orofacial neuropathic pain. |
| Li, 2013 [80] |  | Vc/C2 region | Vibrissal pad |  |  |  | Vc/V2 |  | Infraorbital nerve | Cervical: C1, C2  Other areas: Vc |  |  |  | Infraorbital nerve injury leads to TSP4 upregulation in trigeminal spinal complex that contributes to orofacial neuropathic pain states. |
| Li, 2014 [81] |  | C1 | Face |  |  |  | Vc/V2 |  | Infraorbital nerve | Cervical: upper cervical cord (C1-C2)  Other areas: trigeminal subnucleus caudalis |  |  |  | CCI-ION-induced Cav21 up-regulation may contribute to orofacial neuropathic pain states through abnormal excitatory synapse formation and enhanced presynaptic excitatory neurotransmitter release in Vc/C2. |
| Liu, 2012 [82] |  | trapezius muscle | Facial skin |  |  |  | Vc/V2 |  | Tongue | Cervical: Upper cervical spinal cord (C1-C2)  Other areas: Trigeminal spinal Vc |  |  |  | It is a new animal model of inflammatory tongue pain in rodents, and demonstrated pivotal roles of the mGluR5-pERK signaling in the development of mechanical and heat hypersensitivity that evolved in the inflamed tongue. |
| Sessle, 1986 [66] |  | Cervical skin and muscle | NA |  |  |  |  |  | Facial skin, oral mucosa, canine and premolar tooth pulp,  laryngeal mucosa, jaw, tongue muscles | Other areas: trigeminal (V) subnucleus caudalis (medullary dorsal horn) at levels O-3 mm caudal to the obex. |  |  |  | The stimulus on the orofacial area demonstrated an improvement of the neuronal activity at the cervical area of the TCN. |
| Wetsberg and Olsson, 1991 [68] |  | Cervical nerves | NA |  |  |  |  |  | Trigeminal, facial | Other areas: trigeminal tract; different trigeminal sensory nuclei |  |  |  | The stimulus applied at the trigeminal tract trigger inputs at the orofacial region. |
| Zerari-Mailly, 2003 [83] |  | NR | NR |  |  |  |  |  | Main branches of the left SO nerve | Cervical: Vc-C2  Other areas: Vo/Vi |  |  |  | The present results demonstrate the existence of neurons in the Vi/Vc and C1 spinal cord that receive direct inputs from the eyelids and project to the SO nerve |

The direction was not studied; There is a connection; There is no connection The results are not clear.

TNC: Trigeminocervical nucleus; TMJ: Temporomandibular joint; NA: Not applicable; NR: Not reported; SO: Supraorbital nerve; TMD: Temporomandibular disorders; Vc: Trigeminal spinal subnucleus caudalis; TBNC: Trigemino brainsterm nuclear complex; Fos-li: Fos-like immunoreactivity; UCC: Upper cervical cord; C1-C2-C3-C4-C5-C6: Cervical levels; Bont-A: Botulinum neurotoxin A; Vi: Trigeminal spinal nucleus interpolaris; CNX: Upper cervical spinal nerve transection; IAN: inferior alveolar nerve; FKN: Fractalkine; TP: Tooth pulp; PN: Phrenic nerve; MDH: Medullary dorsal horn; TG: Trigeminal ganglia; ION: infraorbital nerve; ION-CC: constriction.

References

[1] Bereiter DA, Bereiter DF, Ramos M. Vagotomy prevents morphine-induced reduction in Fos-like immunoreactivity in trigeminal spinal nucleus produced after TMJ injury in a sex-dependent manner. Pain. 2002; 96: 205–213.

[2] Bereiter DA. Sex differences in brainstem neural activation after injury to the TMJ region. Cells Tissues Organs. 2001; 169: 226–237.

[3] Casatti CA, Frigo L, Bauer JA. Origin of sensory and autonomic innervation of the rat temporomandibular joint: a retrograde axonal tracing study with the fluorescent dye fast blue. Journal of Dental Research. 1999; 78: 776–783.

[4] Du BL, Li JN, Guo HM, Li S, Liu B. The effect of functional mandibular shift on the muscle spindle systems in head-neck muscles and the related neurotransmitter histamine. Journal of Craniofacial Surgery. 2017; 28: 1628–1634.

[5] Hathaway CB, Hu JW, Bereiter DA. Distribution of Fos-like immunoreactivity in the caudal brainstem of the rat following noxious chemical stimulation of the temporomandibular joint. Journal of Comparative Neurology. 1995; 356: 444–456.

[6] Kramer PR, Bellinger LL. Reduced GABAA receptor α6 expression in the trigeminal ganglion enhanced myofascial nociceptive response. Neuroscience. 2013; 245: 1–11.

[7] Imbe H, Ren K. Orofacial deep and cutaneous tissue inflammation differentially upregulates preprodynorphin mRNA in the trigeminal and paratrigeminal nuclei of the rat. Brain Research Molecular Brain Research. 1999; 67: 87–97.

[8] Lam DK, Sessle BJ, Hu JW. Glutamate and capsaicin effects on trigeminal nociception II: activation and central sensitization in brainstem neurons with deep craniofacial afferent input. Brain Research. 2009; 1253: 48–59.

[9] Lam DK, Sessle BJ, Hu JW. Surgical incision can alter capsaicin-induced central sensitization in rat brainstem nociceptive neurons. Neuroscience. 2008; 156: 737–747.

[10] Okamoto K, Bereiter DF, Thompson R, Tashiro A, Bereiter DA. Estradiol replacement modifies c-fos expression at the spinomedullary junction evoked by temporomandibular joint stimulation in ovariectomized female rats. Neuroscience. 2008; 156: 729–736.

[11] Okamoto K, Kimura A, Donishi T, Imbe H, Senba E, Tamai Y. Central serotonin 3 receptors play an important role in the modulation of nociceptive neural activity of trigeminal subnucleus caudalis and nocifensive orofacial behavior in rats with persistent temporomandibular joint inflammation. Neuroscience. 2005; 135: 569–581.

[12] Puri J, Bellinger LL, Kramer PR. Estrogen in cycling rats alters gene expression in the temporomandibular joint, trigeminal ganglia and trigeminal subnucleus caudalis/upper cervical cord junction. Journal of Cellular Physiology. 2011; 226: 3169–3180.

[13] Zhou Q, Imbe H, Dubner R, Ren K. Persistent Fos protein expression after orofacial deep or cutaneous tissue inflammation in rats: Implications for persistent orofacial pain. Journal of Comparative Neurology. 1999; 412: 276–291.

[14] Takeshita S, Hirata H, Bereiter DA. Intensity coding by TMJ-responsive neurons in superficial laminae of caudal medullary dorsal horn of the rat. Journal of Neurophysiology. 2001; 86: 2393–2404.

[15] Marfurt CF, Rajchert DM. Trigeminal primary afferent projections to “non-trigeminal” areas of the rat central nervous system. Journal of Comparative Neurology. 1991; 303: 489–511.

[16] Chibuzo GA, Cummings JF. The origins of the afferent fibers to the lingual muscles of the dog, a retrograde labelling study with horseradish peroxidase. Anatomical Record. 1981; 200: 95–101.

[17] Adachi K, Shimizu K, Hu JW, Suzuki I, Sakagami H, Koshikawa N, *et al*. Purinergic receptors are involved in tooth-pulp evoked nocifensive behavior and brainstem neuronal activity. Molecular Pain. 2010; 6: 59.

[18] Tanimoto T, Takeda M, Matsumoto S. Suppressive effect of vagal afferents on cervical dorsal horn neurons responding to tooth pulp electrical stimulation in the rat. Experimental Brain Research. 2002; 145: 468–479.

[19] Tanimoto T, Takeda M, Nishikawa T, Matsumoto S. The role of 5-hydroxytryptamine_3_ receptors in the vagal afferent activation-induced inhibition of the first cervical dorsal horn spinal neurons projected from tooth pulp in the rat. Journal of Pharmacology and Experimental Therapeutics. 2004; 311: 803–810.

[20] Marfurt CF, Turner DF. The central projections of tooth pulp afferent neurons in the rat as determined by the transganglionic transport of horseradish peroxidase. Journal of Comparative Neurology. 1984; 223: 535–547.

[21] Sabino MA, Honore P, Rogers SD, Mach DB, Luger NM, Mantyh PW. Tooth extraction-induced internalization of the substance P receptor in trigeminal nucleus and spinal cord neurons: imaging the neurochemistry of dental pain. Pain. 2002; 95: 175–186.

[22] Shimizu K, Asano M, Kitagawa J, Ogiso B, Ren K, Oki H, *et al*. Phosphorylation of extracellular signal-regulated kinase in medullary and upper cervical cord neurons following noxious tooth pulp stimulation. Brain Research. 2006; 1072: 99–109.

[23] Matsumoto S, Takeda M, Tanimoto T. Effects of electrical stimulation of the tooth pulp and phrenic nerve fibers on C-1 spinal neurons in the rat. Experimental Brain Research. 1999; 126: 351–358.

[24] Kato S, Papuashvili N, Okada YC. Identification and functional characterization of the trigeminal ventral cervical reflex pathway in the swine. Clinical Neurophysiology. 2003; 114: 263–271.

[25] Busch V, Jakob W, Juergens T, Schulte-Mattler W, Kaube H, May A. Functional connectivity between trigeminal and occipital nerves revealed by occipital nerve blockade and nociceptive blink reflexes. Cephalalgia. 2006; 26: 50–55.

[26] Chudler EH, Foote WE, Poletti CE. Topography of C1 nerve- and trigeminal-evoked potentials in the ventrobasal complex of the cat thalamus. Neuroscience Letters. 1991; 132: 33–36.

[27] Classey JD, Knight YE, Goadsby PJ. The NMDA receptor antagonist MK-801 reduces Fos-like immunoreactivity within the trigeminocervical complex following superior sagittal sinus stimulation in the cat. Brain Research. 2001; 907: 117–124.

[28] Noma N, Watanabe K, Sato Y, Imamura Y, Yamamoto Y, Ito R, *et al*. Botulinum neurotoxin type A alleviates mechanical hypersensitivity associated with infraorbital nerve constriction injury in rats. Neuroscience Letters. 2017; 637: 96–101.

[29] Nomura H, Ogawa A, Tashiro A, Morimoto T, Hu JW, Iwata K. Induction of Fos protein-like immunoreactivity in the trigeminal spinal nucleus caudalis and upper cervical cord following noxious and non-noxious mechanical stimulation of the whisker pad of the rat with an inferior alveolar nerve transection. Pain. 2002; 95: 225–238.

[30] Suzuki I, Kitagawa J, Noma N, Tsuboi Y, Kondo M, Honda K, *et al*. Attenuation of naloxone-induced Vc pERK hyper-expression following capsaicin stimulation of the face in aged rat. Neuroscience Letters. 2008; 442: 39–43.

[31] Kamimura R, Hossain MZ, Unno S, Ando H, Masuda Y, Takahashi K, *et al*. Inhibition of 2-arachydonoylgycerol degradation attenuates orofacial neuropathic pain in trigeminal nerve-injured mice. Journal of Oral Science. 2018; 60: 37–44.

[32] Honda K, Noma N, Shinoda M, Miyamoto M, Katagiri A, Kita D, *et al*. Involvement of peripheral ionotropic glutamate receptors in orofacial thermal hyperalgesia in rats. Molecular Pain. 2011; 7: 75.

[33] Hu JW, Sessle BJ, Raboisson P, Dallel R, Woda A. Stimulation of craniofacial muscle afferents induces prolonged facilitatory effects in trigeminal nociceptive brain-stem neurones. Pain. 1992; 48: 53–60.

[34] Kramer PR, Bellinger LL. Infusion of Gabrα6 siRNA into the trigeminal ganglia increased the myogenic orofacial nociceptive response of ovariectomized rats treated with 17β-estradiol. Neuroscience. 2014; 278: 144–153.

[35] Kubo A, Sugawara S, Iwata K, Yamaguchi S, Mizumura K. Masseter muscle contraction and cervical muscle sensitization by nerve growth factor cause mechanical hyperalgesia in masticatory muscle with activation of the trigemino-lateral parabrachial nucleus system in female rats. Headache. 2022; 62: 1365–1375.

[36] Kurose M, Imbe H, Nakatani Y, Hasegawa M, Fujii N, Takagi R, *et al.* Bilateral increases in ERK activation at the spinomedullary junction region by acute masseter muscle injury during temporomandibular joint inflammation in the rats. Experimental Brain Research. 2017; 235: 913–921.

[37] Okamoto K, Imbe H, Kimura A, Donishi T, Tamai Y, Senba E. Activation of central 5HT2A receptors reduces the craniofacial nociception of rats. Neuroscience. 2007; 147: 1090–1102.

[38] Luo P, Wong R, Dessem D. Projection of jaw-muscle spindle afferents to the caudal brainstem in rats demonstrated using intracellular biotinamide. Journal of Comparative Neurology. 1995; 358: 63–78.

[39] Nishimori T, Sera M, Suemune S, Yoshida A, Tsuru K, Tsuiki Y, *et al*. The distribution of muscle primary afferents from the masseter nerve to the trigeminal sensory nuclei. Brain Res. 1986; 372: 375–381.

[40] Shigenaga Y, Chen IC, Suemune S, Nishimori T, Nasution ID, Yoshida A, *et al*. Oral and facial representation within the medullary and upper cervical dorsal horns in the cat. Journal of Comparative Neurology. 1986; 243: 388–408.

[41] Chiaia NL, Hess PR, Hosoi M, Rhoades RW. Morphological characteristics of low-threshold primary afferents in the trigeminal subnuclei interpolaris and caudalis (the medullary dorsal horn) of the golden hamster. Journal of Comparative Neurology. 1987; 264: 527–546.

[42] Demartini C, Tassorelli C, Zanaboni AM, Tonsi G, Francesconi O, Nativi C, *et al.* The role of the transient receptor potential ankyrin type-1 (TRPA1) channel in migraine pain: evaluation in an animal model. Journal of Headache & Pain. 2017; 18: 94.

[43] Goadsby PJ, Zagami AS. Stimulation of the superior sagittal sinus increases metabolic activity and blood flow in certain regions of the brainstem and upper cervical spinal cord of the cat. Brain. 1991; 114: 1001–1011.

[44] Kaube H, Keay KA, Hoskin KL, Bandler R, Goadsby PJ. Expression of c-Fos-like immunoreactivity in the caudal medulla and upper cervical spinal cord following stimulation of the superior sagittal sinus in the cat. Brain Research. 1993; 629: 95–102.

[45] Honda K, Kitagawa J, Sessle BJ, Kondo M, Tsuboi Y, Yonehara Y, *et al*. Mechanisms involved in an increment of multimodal excitability of medullary and upper cervical dorsal horn neurons following cutaneous capsaicin treatment. Molecular Pain. 2008; 4: 59.

[46] Hu JW, Sun KQ, Vernon H, Sessle BJ. Craniofacial inputs to upper cervical dorsal horn: implications for somatosensory information processing. Brain Research. 2005; 1044: 93–106.

[47] Jacquin MF, Renehan WE, Mooney RD, Rhoades RW. Structure-function relationships in rat medullary and cervical dorsal horns. I. Trigeminal primary afferents. Journal of Neurophysiology. 1986; 55: 1153–1186.

[48] Jacquin MF, Rhoades RW. Central projections of the normal and ‘regenerate’ infraorbital nerve in adult rats subjected to neonatal unilateral infraorbital lesions: a transganglionic horseradish peroxidase study. Brain Research. 1983; 269: 137–144.

[49] Luz LL, Fernandes EC, Dora F, Lukoyanov NV, Szucs P, Safronov BV. Trigeminal Aδ- and C-afferent supply of lamina I neurons in the trigeminocervical complex. Pain. 2019; 160: 2612–2623.

[50] Lyubashina OA, Sokolov AY, Panteleev SS. Vagal afferent modulation of spinal trigeminal neuronal responses to dural electrical stimulation in rats. Neuroscience. 2012; 222: 29–37.

[51] Lyubashina OA, Panteleev SS, Sokolov AY. Inhibitory effect of high-frequency greater occipital nerve electrical stimulation on trigeminovascular nociceptive processing in rats. Journal of Neural Transmission. 2017; 124: 171–183.

[52] Margolis TP, LaVail JH, Setzer PY, Dawson CR. Selective spread of herpes simplex virus in the central nervous system after ocular inoculation. Journal of Virology. 1989; 63: 4756–4761.

[53] Morch CD, Hu JW, Arendt-Nielsen L, Sessle BJ. Convergence of cutaneous, musculoskeletal, dural and visceral afferents onto nociceptive neurons in the first cervical dorsal horn. European Journal of Neuroscience. 2007; 26: 142–154.

[54] Nakajima A, Tsuboi Y, Suzuki I, Honda K, Shinoda M, Kondo M, *et al.* PKCgamma in Vc and C1/C2 is involved in trigeminal neuropathic pain. Journal of Dental Research. 2011; 90: 777–781.

[55] Noma N, Tsuboi Y, Kondo M, Matsumoto M, Sessle BJ, Kitagawa J, *et al*. Organization of pERK-immunoreactive cells in trigeminal spinal nucleus caudalis and upper cervical cord following capsaicin injection into oral and craniofacial regions in rats. Journal of Comparative Neurology. 2008; 507: 1428–1440.

[56] Park J, Trinh VN, Sears-Kraxberger I, Li KW, Steward O, Luo ZD. Synaptic ultrastructure changes in trigeminocervical complex posttrigeminal nerve injury. Journal of Comparative Neurology. 2016; 524: 309–322.

[57] Panneton WM. Primary afferent projections from the upper respiratory tract in the muskrat. Journal of Comparative Neurology. 1991; 308: 51–65.

[58] Ogawa A, Ren K, Tsuboi Y, Morimoto T, Sato T, Iwata K. A new model of experimental parotitis in rats and its implication for trigeminal nociception. Experimental Brain Research. 2003; 152: 307–316.

[59] Ogawa A, Meng ID, Ren K, Imamura Y, Iwata K. Differential responses of rostral subnucleus caudalis and upper cervical dorsal horn neurons to mechanical and chemical stimulation of the parotid gland in rats. Brain Research. 2006; 1106: 123–133.

[60] Sato T, Kitagawa J, Ren K, Tanaka H, Tanabe A, Watanabe T, *et al*. Activation of trigeminal intranuclear pathway in rats with temporomandibular joint inflammation. Journal of Oral Science. 2005; 47: 65–69.

[61] Shibuta K, Suzuki I, Shinoda M, Tsuboi Y, Honda K, Shimizu N, *et al.* Organization of hyperactive microglial cells in trigeminal spinal subnucleus caudalis and upper cervical spinal cord associated with orofacial neuropathic pain. Brain Research. 2012; 1451: 74–86.

[62] Strassman AM, Mineta Y, Vos BP. Distribution of fos-like immunoreactivity in the medullary and upper cervical dorsal horn produced by stimulation of dural blood vessels in the rat. Journal of Neuroscience. 1994; 14: 3725–3735.

[63] Strassman AM, Vos BP. Somatotopic and laminar organization of fos-like immunoreactivity in the medullary and upper cervical dorsal horn induced by noxious facial stimulation in the rat. Journal of Comparative Neurology. 1993; 331: 495–516.

[64] Sugimoto T, Hara T, Shirai H, Abe T, Ichikawa H, Sato T. c-fos induction in the subnucleus caudalis following noxious mechanical stimulation of the oral mucous membrane. Experimental Neurology. 1994; 129: 251–256.

[65] Takemura M, Sugimoto T, Sakai A. Topographic organization of central terminal region of different sensory branches of the rat mandibular nerve. Experimental Neurology. 1987; 96: 540–557.

[66] Sessle BJ, Hu JW, Amano N, Zhong G. Convergence of cutaneous, tooth pulp, visceral, neck and muscle afferents onto nociceptive and non-nociceptive neurones in trigeminal subnucleus caudalis (medullary dorsal horn) and its implications for referred pain. Pain. 1986; 27: 219–235.

[67] Sugimoto T, Fujiyoshi Y, Xiao C, He YF, Ichikawa H. Central projection of calcitonin gene-related peptide (CGRP)- and substance P (SP)-immunoreactive trigeminal primary neurons in the rat. The Journal of Comparative Neurology. 1997; 378: 425–442.

[68] Westberg KG, Olsson KA. Integration in trigeminal premotor interneurones in the cat. 1. Functional characteristics of neurones in the subnucleus-gamma of the oral nucleus of the spinal trigeminal tract. Experimental Brain Research. 1991; 84: 102–114.

[69] Shigenaga Y, Okamoto T, Nishimori T, Suemune S, Nasution I, Chen I, *et al*. Oral and facial representation in the trigeminal principal and rostral spinal nuclei of the cat. Journal of Comparative Neurology. 1986; 244: 1–18.

[70] Shigenaga Y, Sera M, Nishimori T, Suemune S, Nishimura M, Yoshida A, *et al*. The central projection of masticatory afferent fibers to the trigeminal sensory nuclear complex and upper cervical spinal cord. Journal of Comparative Neurology. 1988; 268: 489–507.

[71] Tashiro A, Okamoto K, Bereiter DA. Chronic inflammation and estradiol interact through MAPK activation to affect TMJ nociceptive processing by trigeminal caudalis neurons. Neuroscience. 2009; 164: 1813–1820.

[72] Yasuda K, Furusawa K, Tanaka M, Yamaoka M. The distribution of afferent neurons in the trigeminal mesencephalic nucleus and the central projection of afferent fibers of the mylohyoid nerve in the rat. Somatosensory & Motor Research. 1995; 12: 309–315.

[73] Vos BP, Strassman AM. Fos expression in the medullary dorsal horn of the rat after chronic constriction injury to the infraorbital nerve. The Journal of Comparative Neurology. 1995; 357: 362–375.

[74] Young RF, Perryman KM. Pathways for orofacial pain sensation in the trigeminal brain-stem nuclear complex of the Macaque monkey. Journal of Neurosurgery. 1984; 61: 563–568.

[75] Kiyomoto M, Shinoda M, Okada-Ogawa A, Noma N, Shibuta K, Tsuboi Y, *et al*. Fractalkine signaling in microglia contributes to ectopic orofacial pain following trapezius muscle inflammation. Journal of Neuroscience. 2013; 33: 7667–7680.

[76] Kobayashi A, Shinoda M, Sessle BJ, Honda K, Imamura Y, Hitomi S, *et al.* Mechanisms involved in extraterritorial facial pain following cervical spinal nerve injury in rats. Molecular Pain. 2011; 7: 12.

[77] Kiyomoto M, Shinoda M, Honda K, Nakaya Y, Dezawa K, Katagiri A, *et al*. p38 phosphorylation in medullary microglia mediates ectopic orofacial inflammatory pain in rats. Molecular Pain. 2015; 11: 48.

[78] Miyamoto M, Tsuboi Y, Takamiya K, Huganir RL, Kondo M, Shinoda M, *et al.* Involvement of GluR2 and GluR3 subunit C-termini in the trigeminal spinal subnucleus caudalis and C1-C2 neurons in trigeminal neuropathic pain. Neuroscience Letters. 2011; 491: 8–12.

[79] Qu ZY, Liu L, Zhao LP, Xu XB, Li ZJ, Zhu YP, *et al*. Prophylactic Electroacupuncture on the upper cervical segments decreases neuronal discharges of the trigeminocervical complex in migraine-affected rats: an *in vivo* extracellular electrophysiological experiment. Journal of Pain Research. 2020; 13: 25–37.

[80] Li CX, Yang Q, Vemulapalli S, Waters RS. Forelimb amputation-induced reorganization in the cuneate nucleus (CN) is not reflected in large-scale reorganization in rat forepaw barrel subfield cortex (FBS). Brain Research. 2013; 1526: 26–43.

[81] Li KW, Yu YP, Zhou C, Kim DS, Lin B, Sharp K, *et al*. Calcium channel α2δ1 proteins mediate trigeminal neuropathic pain states associated with aberrant excitatory synaptogenesis. Journal of Biological Chemistry. 2014; 289: 7025–7037.

[82] Liu MG, Matsuura S, Shinoda M, Honda K, Suzuki I, Shibuta K, *et al*. Metabotropic glutamate receptor 5 contributes to inflammatory tongue pain via extracellular signal-regulated kinase signaling in the trigeminal spinal subnucleus caudalis and upper cervical spinal cord. Journal of Neuroinflammation. 2012; 9: 258.

[83] Zerari-Mailly F, Dauvergne C, Buisseret P, Buisseret-Delmas C. Localization of trigeminal, spinal, and reticular neurons involved in the rat blink reflex. Journal of Comparative Neurology. 2003; 467: 173–184.
