# Supplementary material for: Integration of nociceptive activity from orofacial, cranial and cervical regions in the trigeminocervical nucleus: a scoping review with clinical implications
Source: J Oral Facial Pain Headache. 2025 Sep 12;39(3):1–12. doi: 10.22514/jofph.2025.042 (PMC12531578; doi:10.22514/jofph.2025.042)
Supplement: Supplementary file 5 [file Supplementary-material-5.docx]

Supplementary material

Supplementary material 5. Overall Summary of connections between the cranial, orofacial and cervical regions through the TCN and directions.

Supplementary Table 1. Cervical to Orofacial/cranial (n = 5).

| Number of studies | Cervical to orofacial | Stimulus Cervical | Area of Response Orofacial | TCN Area  Vc/Vi | Spinal cord | Vo/Vi | Others |
| --- | --- | --- | --- | --- | --- | --- | --- |
| 5 | 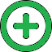 | trapezius muscle (3) [1–3]; Neck (2) [4, 5] | Face/Facial skin (5) | 5 | 0 | 5 | 2 |


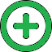
There is a connection. TCN: trigeminocervical nucleus.

Supplementary Table 2. Orofacial/cranial to cervical (n = 72).

| Number of studies | Orofacial to cervical | Stimulus Orofacial | Area of Response Cervical | TCN Area  Vc/Vi | Spinal cord | Vo/Vi | Others |
| --- | --- | --- | --- | --- | --- | --- | --- |
| 64 | 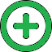 | TMJ (12) [6–21]; tooth pulp (7) [22–28] whisker pad (5) [29–33]; masseter muscle (4) [14, 34–36]; infraorbital nerve (5) [37–41]; parotid gland (2) [42, 43]; Forehead/Head [44, 45] Orofacial Region (unspecific) [37, 46–70] | Splenius and trapezius muscles; Brainstem/Cervical muscles; TCN caudalis, and upper cervical region | 52* | 17* | 9* | 26* |
| 2 | 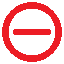 | TMJ [71]; not applicable (NA) [72] | TCN caudalis and upper cervical region; NA | 1 | 0 | 1 | 0 |
| 5 | 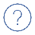 | Left vagal nerve [73]; TMJ [74, 75]; supraorbital nerve [76]; Trigeminal nerve [77] | C1/spinal cord; Trapezius muscle; C2 |  |  |  |  |

*Numbers do not add up since studies are considered in more than one column.


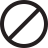
 The direction was not studied;
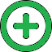
There is a connection;
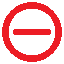
There is no connection
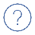
The results are not clear.

ION: infraorbital nerve; TMJ: temporomandibular joint; TCN: trigeminocervical nucleus; NA: Not applicable.

Supplementary Table 3. Studies investigating both directions (n = 7).

| Number of studies | Cervical to orofacial | Stimulus Cervical/Orofacial | Area of Response | TCN Area  Vc/Vi | Spinal cord | Vo/Vi | Others |
| --- | --- | --- | --- | --- | --- | --- | --- |
| 6 | 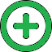 | C1/Infraorbital nerve [78, 79], C2/Infraorbital nerve [80], Trapezius muscle/Tongue [81], Cervical skin/NA [82], Cervical nerves/NA [83], NA/NA [78] | Face (3); Facial skin (2); Vibrissal pad; supraorbital nerve | 3 | 1 | 4 | 0 |


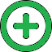
There is a connection. TCN: trigeminocervical nucleus. NA: not applicable.

References

[1] Kiyomoto M, Shinoda M, Honda K, Nakaya Y, Dezawa K, Katagiri A, *et al*. p38 phosphorylation in medullary microglia mediates ectopic orofacial inflammatory pain in rats. Molecular Pain. 2015; 11: 48.

[2] Kiyomoto M, Shinoda M, Okada-Ogawa A, Noma N, Shibuta K, Tsuboi Y, *et al*. Fractalkine signaling in microglia contributes to ectopic orofacial pain following trapezius muscle inflammation. Journal of Neuroscience. 2013; 33: 7667–7680.

[3] Liu MG, Matsuura S, Shinoda M, Honda K, Suzuki I, Shibuta K, *et al*. Metabotropic glutamate receptor 5 contributes to inflammatory tongue pain via extracellular signal-regulated kinase signaling in the trigeminal spinal subnucleus caudalis and upper cervical spinal cord. Journal of Neuroinflammation. 2012; 9: 258.

[4] Kobayashi A, Shinoda M, Sessle BJ, Honda K, Imamura Y, Hitomi S, *et al*. Mechanisms involved in extraterritorial facial pain following cervical spinal nerve injury in rats. Molecular Pain. 2011; 7: 12.

[5] Qu ZY, Liu L, Zhao LP, Xu XB, Li ZJ, Zhu YP, *et al*. Prophylactic electroacupuncture on the upper cervical segments decreases neuronal discharges of the trigeminocervical complex in migraine-affected rats: an *in vivo* extracellular electrophysiological experiment. Journal of Pain Research. 2020; 13: 25–37.

[6] Bereiter DA. Sex differences in brainstem neural activation after injury to the TMJ region. Cells Tissues Organs. 2001; 169: 226–237.

[7] Bereiter DA, Bereiter DF, Ramos M. Vagotomy prevents morphine-induced reduction in Fos-like immunoreactivity in trigeminal spinal nucleus produced after TMJ injury in a sex-dependent manner. Pain. 2002; 96: 205–213.

[8] Casatti CA, Frigo L, Bauer JA. Origin of sensory and autonomic innervation of the rat temporomandibular joint: a retrograde axonal tracing study with the fluorescent dye fast blue. Journal of Dental Research. 1999; 78: 776–783.

[9] Hathaway CB, Hu JW, Bereiter DA. Distribution of Fos-like immunoreactivity in the caudal brainstem of the rat following noxious chemical stimulation of the temporomandibular joint. Journal of Comparative Neurology. 1995; 356: 444–456.

[10] Tashiro A, Okamoto K, Bereiter DA. Chronic inflammation and estradiol interact through MAPK activation to affect TMJ nociceptive processing by trigeminal caudalis neurons. Neuroscience. 2009; 164: 1813–1820.

[11] Shigenaga Y, Sera M, Nishimori T, Suemune S, Nishimura M, Yoshida A, *et al*. The central projection of masticatory afferent fibers to the trigeminal sensory nuclear complex and upper cervical spinal cord. Journal of Comparative Neurology. 1988; 268: 489–507.

[12] Imbe H, Ren K. Orofacial deep and cutaneous tissue inflammation differentially upregulates preprodynorphin mRNA in the trigeminal and paratrigeminal nuclei of the rat. Brain Research Molecular Brain Research. 1999; 67: 87–97.

[13] Lam DK, Sessle BJ, Hu JW. Glutamate and capsaicin effects on trigeminal nociception II: activation and central sensitization in brainstem neurons with deep craniofacial afferent input. Brain Research. 2009; 1253: 48–59.

[14] Shigenaga Y, Chen IC, Suemune S, Nishimori T, Nasution ID, Yoshida A, *et al*. Oral and facial representation within the medullary and upper cervical dorsal horns in the cat. Journal of Comparative Neurology. 1986; 243: 388–408.

[15] Shigenaga Y, Okamoto T, Nishimori T, Suemune S, Nasution I, Chen I, *et al*. Oral and facial representation in the trigeminal principal and rostral spinal nuclei of the cat. Journal of Comparative Neurology. 1986; 244: 1–18.

[16] Okamoto K, Bereiter DF, Thompson R, Tashiro A, Bereiter DA. Estradiol replacement modifies c-fos expression at the spinomedullary junction evoked by temporomandibular joint stimulation in ovariectomized female rats. Neuroscience. 2008; 156: 729–736.

[17] Okamoto K, Kimura A, Donishi T, Imbe H, Senba E, Tamai Y. Central serotonin 3 receptors play an important role in the modulation of nociceptive neural activity of trigeminal subnucleus caudalis and nocifensive orofacial behavior in rats with persistent temporomandibular joint inflammation. Neuroscience. 2005; 135: 569–581.

[18] Puri J, Bellinger LL, Kramer PR. Estrogen in cycling rats alters gene expression in the temporomandibular joint, trigeminal ganglia and trigeminal subnucleus caudalis/upper cervical cord junction. Journal of Cellular Physiology. 2011; 226: 3169–3180.

[19] Zhou Q, Imbe H, Dubner R, Ren K. Persistent Fos protein expression after orofacial deep or cutaneous tissue inflammation in rats: implications for persistent orofacial pain. Journal of Comparative Neurology. 1999; 412: 276–291.

[20] Takeshita S, Hirata H, Bereiter DA. Intensity coding by TMJ-responsive neurons in superficial laminae of caudal medullary dorsal horn of the rat. Journal of Neurophysiology. 2001; 86: 2393–2404.

[21] Marfurt CF, Rajchert DM. Trigeminal primary afferent projections to “non-trigeminal” areas of the rat central nervous system. Journal of Comparative Neurology. 1991; 303: 489–511.

[22] Adachi K, Shimizu K, Hu JW, Suzuki I, Sakagami H, Koshikawa N, *et al*. Purinergic receptors are involved in tooth-pulp evoked nocifensive behavior and brainstem neuronal activity. Molecular Pain. 2010; 6: 59.

[23] Tanimoto T, Takeda M, Matsumoto S. Suppressive effect of vagal afferents on cervical dorsal horn neurons responding to tooth pulp electrical stimulation in the rat. Experimental Brain Research. 2002; 145: 468–479.

[24] Tanimoto T, Takeda M, Nishikawa T, Matsumoto S. The role of 5-hydroxytryptamine_3_ receptors in the vagal afferent activation-induced inhibition of the first cervical dorsal horn spinal neurons projected from tooth pulp in the rat. Journal of Pharmacology and Experimental Therapeutics. 2004; 311: 803–810.

[25] Marfurt CF, Turner DF. The central projections of tooth pulp afferent neurons in the rat as determined by the transganglionic transport of horseradish peroxidase. Journal of Comparative Neurology. 1984; 223: 535–547.

[26] Sabino MA, Honore P, Rogers SD, Mach DB, Luger NM, Mantyh PW. Tooth extraction-induced internalization of the substance P receptor in trigeminal nucleus and spinal cord neurons: imaging the neurochemistry of dental pain. Pain. 2002; 95: 175–186.

[27] Shimizu K, Asano M, Kitagawa J, Ogiso B, Ren K, Oki H, *et al*. Phosphorylation of extracellular signal-regulated kinase in medullary and upper cervical cord neurons following noxious tooth pulp stimulation. Brain Research. 2006; 1072: 99–109.

[28] Matsumoto S, Takeda M, Tanimoto T. Effects of electrical stimulation of the tooth pulp and phrenic nerve fibers on C-1 spinal neurons in the rat. Experimental Brain Research. 1999; 126: 351–358.

[29] Noma N, Watanabe K, Sato Y, Imamura Y, Yamamoto Y, Ito R, *et al*. Botulinum neurotoxin type A alleviates mechanical hypersensitivity associated with infraorbital nerve constriction injury in rats. Neuroscience Letters. 2017; 637: 96–101.

[30] Nomura H, Ogawa A, Tashiro A, Morimoto T, Hu JW, Iwata K. Induction of Fos protein-like immunoreactivity in the trigeminal spinal nucleus caudalis and upper cervical cord following noxious and non-noxious mechanical stimulation of the whisker pad of the rat with an inferior alveolar nerve transection. Pain. 2002; 95: 225–238.

[31] Suzuki I, Kitagawa J, Noma N, Tsuboi Y, Kondo M, Honda K, *et al*. Attenuation of naloxone-induced Vc pERK hyper-expression following capsaicin stimulation of the face in aged rat. Neuroscience Letters. 2008; 442: 39–43.

[32] Honda K, Noma N, Shinoda M, Miyamoto M, Katagiri A, Kita D, *et al*. Involvement of peripheral ionotropic glutamate receptors in orofacial thermal hyperalgesia in rats. Molecular Pain. 2011; 7: 75.

[33] Kamimura R, Hossain MZ, Unno S, Ando H, Masuda Y, Takahashi K, *et al*. Inhibition of 2-arachydonoylgycerol degradation attenuates orofacial neuropathic pain in trigeminal nerve-injured mice. Journal of Oral Science. 2018; 60: 37–44.

[34] Hu JW, Sessle BJ, Raboisson P, Dallel R, Woda A. Stimulation of craniofacial muscle afferents induces prolonged facilitatory effects in trigeminal nociceptive brain-stem neurones. Pain. 1992; 48: 53–60.

[35] Kramer PR, Bellinger LL. Infusion of Gabrα6 siRNA into the trigeminal ganglia increased the myogenic orofacial nociceptive response of ovariectomized rats treated with 17beta-estradiol. Neuroscience. 2014; 278: 144–153.

[36] Okamoto K, Imbe H, Kimura A, Donishi T, Tamai Y, Senba E. Activation of central 5HT2A receptors reduces the craniofacial nociception of rats. Neuroscience. 2007; 147: 1090–1102.

[37] Jacquin MF, Rhoades RW. Central projections of the normal and ‘regenerate’ infraorbital nerve in adult rats subjected to neonatal unilateral infraorbital lesions: a transganglionic horseradish peroxidase study. Brain Research. 1983; 269: 137–144.

[38] Nakajima A, Tsuboi Y, Suzuki I, Honda K, Shinoda M, Kondo M, *et al*. PKCgamma in Vc and C1/C2 is involved in trigeminal neuropathic pain. Journal of Dental Research. 2011; 90: 777–781.

[39] Noma N, Tsuboi Y, Kondo M, Matsumoto M, Sessle BJ, Kitagawa J, *et al*. Organization of pERK-immunoreactive cells in trigeminal spinal nucleus caudalis and upper cervical cord following capsaicin injection into oral and craniofacial regions in rats. Journal of Comparative Neurology. 2008; 507: 1428–1440.

[40] Park J, Trinh VN, Sears-Kraxberger I, Li KW, Steward O, Luo ZD. Synaptic ultrastructure changes in trigeminocervical complex posttrigeminal nerve injury. Journal of Comparative Neurology. 2016; 524: 309–322.

[41] Strassman AM, Vos BP. Somatotopic and laminar organization of fos-like immunoreactivity in the medullary and upper cervical dorsal horn induced by noxious facial stimulation in the rat. Journal of Comparative Neurology. 1993; 331: 495–516.

[42] Ogawa A, Meng ID, Ren K, Imamura Y, Iwata K. Differential responses of rostral subnucleus caudalis and upper cervical dorsal horn neurons to mechanical and chemical stimulation of the parotid gland in rats. Brain Research. 2006; 1106: 123–133.

[43] Ogawa A, Ren K, Tsuboi Y, Morimoto T, Sato T, Iwata K. A new model of experimental parotitis in rats and its implication for trigeminal nociception. Experimental Brain Research. 2003; 152: 307–316.

[44] Busch V, Jakob W, Juergens T, Schulte-Mattler W, Kaube H, May A. Functional connectivity between trigeminal and occipital nerves revealed by occipital nerve blockade and nociceptive blink reflexes. Cephalalgia. 2006; 26: 50–55.

[45] Classey JD, Knight YE, Goadsby PJ. The NMDA receptor antagonist MK-801 reduces Fos-like immunoreactivity within the trigeminocervical complex following superior sagittal sinus stimulation in the cat. Brain Research. 2001; 907: 117–124.

[46] Luo P, Wong R, Dessem D. Projection of jaw-muscle spindle afferents to the caudal brainstem in rats demonstrated using intraceiiuiar biotinamide. The Journal of Comparative Neurology. 1995; 358: 63–78.

[47] Nishimori T, Sera M, Suemune S, Yoshida A, Tsuru K, Tsuiki Y, *et al*. The distribution of muscle primary afferents from the masseter nerve to the trigeminal sensory nuclei. Brain Research. 1986; 372: 375–381.

[48] Chiaia NL, Hess PR, Hosoi M, Rhoades RW. Morphological characteristics of low-threshold primary afferents in the trigeminal subnuclei interpolaris and caudalis (the medullary dorsal horn) of the golden hamster. Journal of Comparative Neurology. 1987; 264: 527–546.

[49] Demartini C, Tassorelli C, Zanaboni AM, Tonsi G, Francesconi O, Nativi C, *et al*. The role of the transient receptor potential ankyrin type-1 (TRPA1) channel in migraine pain: evaluation in an animal model. Journal of Headache & Pain. 2017; 18: 94.

[50] Goadsby PJ, Zagami AS. Stimulation of the superior sagittal sinus increases met abolic activity and blood flow in certain regions of the brainstem and upper cervical spinal cord of the cat. Brain. 1991; 114: 1001–1011.

[51] Kaube H, Keay KA, Hoskin KL, Bandler R, Goadsby PJ. Expression of c-Fos-like immunoreactivity in the caudal medulla and upper cervical spinal cord following stimulation of the superior sagittal sinus in the cat. Brain Research. 1993; 629: 95–102.

[52] Honda K, Kitagawa J, Sessle BJ, Kondo M, Tsuboi Y, Yonehara Y, *et al*. Mechanisms involved in an increment of multimodal excitability of medullary and upper cervical dorsal horn neurons following cutaneous capsaicin treatment. Molecular Pain. 2008; 4: 59.

[53] Hu JW, Sun KQ, Vernon H, Sessle BJ. Craniofacial inputs to upper cervical dorsal horn: implications for somatosensory information processing. Brain Research. 2005; 1044: 93–106.

[54] Jacquin MF, Renehan WE, Mooney RD, Rhoades RW. Structure-function relationships in rat medullary and cervical dorsal horns. I. Trigeminal primary afferents. Journal of Neurophysiology. 1986; 55: 1153–1186.

[55] Luz LL, Fernandes EC, Dora F, Lukoyanov NV, Szucs P, Safronov BV. Trigeminal A delta- and C-afferent supply of lamina I neurons in the trigeminocervical complex. Pain. 2019; 160: 2612–2623.

[56] Margolis TP, LaVail JH, Setzer PY, Dawson CR. Selective spread of herpes simplex virus in the central nervous system after ocular inoculation. Journal of Virology. 1989; 63: 4756–4761.

[57] Panneton WM. Primary afferent projections from the upper respiratory tract in the muskrat. Journal of Comparative Neurology. 1991; 308: 51–65.

[58] Sato T, Kitagawa J, Ren K, Tanaka H, Tanabe A, Watanabe T, *et al*. Activation of trigeminal intranuclear pathway in rats with temporomandibular joint inflammation. Journal of Oral Science. 2005; 47: 65–69.

[59] Shibuta K, Suzuki I, Shinoda M, Tsuboi Y, Honda K, Shimizu N, *et al*. Organization of hyperactive microglial cells in trigeminal spinal subnucleus caudalis and upper cervical spinal cord associated with orofacial neuropathic pain. Brain Research. 2012; 1451: 74–86.

[60] Strassman AM, Mineta Y, Vos BP. Distribution of fos-like immunoreactivity in the medullary and upper cervical dorsal horn produced by stimulation of dural blood vessels in the rat. Journal of Neuroscience. 1994; 14: 3725–3735.

[61] Sugimoto T, Hara T, Shirai H, Abe T, Ichikawa H, Sato T. c-fos induction in the subnucleus caudalis following noxious mechanical stimulation of the oral mucous membrane. Experimental Neurology. 1994; 129: 251–256.

[62] Takemura M, Sugimoto T, Sakai A. Topographic organization of central terminal region of different sensory branches of the rat mandibular nerve. Experimental Neurology. 1987; 96: 540–557.

[63] Sugimoto T, Fujiyoshi Y, Xiao C, He YF, Ichikawa H. Central projection of calcitonin gene-related peptide (cgrp)- and substance P (SP)-immunoreactive trigeminal primary neurons in the rat. The Journal of Comparative Neurology. 1997; 378: 425–442.

[64] Morch CD, Hu JW, Arendt-Nielsen L, Sessle BJ. Convergence of cutaneous, musculoskeletal, dural and visceral afferents onto nociceptive neurons in the first cervical dorsal horn. European Journal of Neuroscience. 2007; 26: 142–154.

[65] Sessle BJ, Hu JW, Amano N, Zhong G. Convergence of cutaneous, tooth pulp, visceral, neck and muscle afferents onto nociceptive and non-nociceptive neurones in trigeminal subnucleus caudalis (medullary dorsal horn) and its implications for referred pain. Pain. 1986; 27: 219–235.

[66] Westberg KG, Olsson KA. Integration in trigeminal premotor interneurones in the cat. 1. Functional characteristics of neurones in the subnucleus-gamma of the oral nucleus of the spinal trigeminal tract. Experimental Brain Research. 1991; 84: 102–114.

[67] Chibuzo GA, Cummings JF. The origins of the afferent fibers to the lingual muscles of the dog, a retrograde labelling study with horseradish peroxidase. Anatomical Record. 1981; 200: 95–101.

[68] Vos BP, Strassman AM. Fos expression in the medullary dorsal horn of the rat after chronic constriction injury to the infraorbital nerve. The Journal of Comparative Neurology. 1995; 357: 362–375.

[69] Kubo A, Sugawara S, Iwata K, Yamaguchi S, Mizumura K. Masseter muscle contraction and cervical muscle sensitization by nerve growth factor cause mechanical hyperalgesia in masticatory muscle with activation of the trigemino-lateral parabrachial nucleus system in female rats. Headache. 2022; 62: 1365–1375.

[70] Kato S, Papuashvili N, Okada YC. Identification and functional characterization of the trigeminal ventral cervical reflex pathway in the swine. Clinical Neurophysiology. 2003; 114: 263–271.

[71] Kramer PR, Bellinger LL. Reduced GABAA receptor alpha6 expression in the trigeminal ganglion enhanced myofascial nociceptive response. Neuroscience. 2013; 245: 1–11.

[72] Lyubashina OA, Panteleev SS, Sokolov AY. Inhibitory effect of high-frequency greater occipital nerve electrical stimulation on trigeminovascular nociceptive processing in rats. Journal of Neural Transmission. 2017; 124: 171–183.

[73] Lyubashina OA, Sokolov AY, Panteleev SS. Vagal afferent modulation of spinal trigeminal neuronal responses to dural electrical stimulation in rats. Neuroscience. 2012; 222: 29–37.

[74] Du BL, Li JN, Guo HM, Li S, Liu B. The effect of functional mandibular shift on the muscle spindle systems in head-neck muscles and the related neurotransmitter histamine. Journal of Craniofacial Surgery. 2017; 28: 1628–1634.

[75] Lam DK, Sessle BJ, Hu JW. Surgical incision can alter capsaicin-induced central sensitization in rat brainstem nociceptive neurons. Neuroscience. 2008; 156: 737–747.

[76] Zerari-Mailly F, Dauvergne C, Buisseret P, Buisseret-Delmas C. Localization of trigeminal, spinal, and reticular neurons involved in the rat blink reflex. Journal of Comparative Neurology. 2003; 467: 173–184.

[77] Li KW, Kim DS, Zaucke F, Luo ZD. Trigeminal nerve injury induced thrombospondin-4 upregulation contributes to orofacial neuropathic pain states in a rat model. European Journal of Pain. 2014; 18: 489–495.

[78] Chudler EH, Foote WE, Poletti CE. Responses of cat C1 spinal cord dorsal and ventral horn neurons to noxious and non-noxious stimulation of the head and face. Brain Research. 1991; 555: 181–192.

[79] Li CX, Yang Q, Vemulapalli S, Waters RS. Forelimb amputation-induced reorganization in the cuneate nucleus (CN) is not reflected in large-scale reorganization in rat forepaw barrel subfield cortex (FBS). Brain Research. 2013; 1526: 26–43.

[80] Kurose M, Imbe H, Nakatani Y, Hasegawa M, Fujii N, Takagi R, *et al*. Bilateral increases in ERK activation at the spinomedullary junction region by acute masseter muscle injury during temporomandibular joint inflammation in the rats. Experimental Brain Research. 2017; 235: 913–921.

[81] Miyamoto M, Tsuboi Y, Takamiya K, Huganir RL, Kondo M, Shinoda M, *et al*. Involvement of GluR2 and GluR3 subunit C-termini in the trigeminal spinal subnucleus caudalis and C1-C2 neurons in trigeminal neuropathic pain. Neuroscience Letters. 2011; 491: 8–12.

[82] Yasuda K, Furusawa K, Tanaka M, Yamaoka M. The distribution of afferent neurons in the trigeminal mesencephalic nucleus and the central projection of afferent fibers of the mylohyoid nerve in the rat. Somatosensory & Motor Research. 1995; 12: 309–315.

[83] Young RF, Perryman KM. Pathways for orofacial pain sensation in the trigeminal brain-stem nuclear complex of the Macaque monkey. Journal of Neurosurgery. 1984; 61: 563–568.
